# One‐shot ^13^C^15^N‐metabolic flux analysis for simultaneous quantification of carbon and nitrogen flux

**DOI:** 10.15252/msb.202211099

**Published:** 2023-01-27

**Authors:** Khushboo Borah Slater, Martin Beyß, Ye Xu, Jim Barber, Catia Costa, Jane Newcombe, Axel Theorell, Melanie J Bailey, Dany J V Beste, Johnjoe McFadden, Katharina Nöh

**Affiliations:** ^1^ Faculty of Health and Medical Sciences University of Surrey Guildford UK; ^2^ Forschungszentrum Jülich GmbH, Institute of Bio‐ and Geosciences, IBG‐1: Biotechnology Jülich Germany; ^3^ Computational Systems Biotechnology RWTH Aachen University Aachen Germany; ^4^ Faculty of Engineering and Physical Sciences University of Surrey Guildford UK; ^5^ Present address: Computational Systems Biology ETH Zürich Basel Switzerland

**Keywords:** Bayesian metabolic flux analysis, carbon metabolism, isotope labeling, *Mycobacterium tuberculosis*, nitrogen metabolism, Metabolism, Methods & Resources, Microbiology, Virology & Host Pathogen Interaction

## Abstract

Metabolic flux is the final output of cellular regulation and has been extensively studied for carbon but much less is known about nitrogen, which is another important building block for living organisms. For the tuberculosis pathogen, this is particularly important in informing the development of effective drugs targeting the pathogen's metabolism. Here we performed ^13^C^15^N dual isotopic labeling of *Mycobacterium bovis* BCG steady state cultures, quantified intracellular carbon and nitrogen fluxes and inferred reaction bidirectionalities. This was achieved by model scope extension and refinement, implemented in a multi‐atom transition model, within the statistical framework of Bayesian model averaging (BMA). Using BMA‐based ^13^C^15^N‐metabolic flux analysis, we jointly resolve carbon and nitrogen fluxes quantitatively. We provide the first nitrogen flux distributions for amino acid and nucleotide biosynthesis in mycobacteria and establish glutamate as the central node for nitrogen metabolism. We improved resolution of the notoriously elusive anaplerotic node in central carbon metabolism and revealed possible operation modes. Our study provides a powerful and statistically rigorous platform to simultaneously infer carbon and nitrogen metabolism in any biological system.

## Graphical abstract

Bayesian ^13^C^15^N‐MFA is an approach for simultaneous quantification of intracellular carbon (C) and nitrogen (N) metabolic fluxes in any living system. It is applied to *Mycobacterium bovis* BCG to measure the first CN flux map, highlighting glutamate as the central node for nitrogen metabolism.

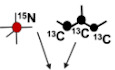

## Introduction

In recent decades, a great deal of progress has been made in unraveling the complexity of intracellular metabolism in microbial, animal, and plant cells by measuring metabolic fluxes through the reactions that constitute central metabolism. The state‐of‐the‐art technique is ^13^C‐Metabolic Flux Analysis (MFA) in which cells, at metabolic steady‐state, are fed a mixture of ^12^C and ^13^C‐labeled substrates that are incorporated into the central carbon (C) metabolism to yield stable end products, such as the proteinogenic amino acids. The method infers *in vivo* metabolic reaction rates (fluxes) by using a system‐wide biochemical reaction model that tracks C atom rearrangements throughout the metabolic pathways and by fitting these fluxes to the emerging labeling patterns (typically isotopically ^12^C and ^13^C labeled fractional enrichments measured by mass spectrometry (MS) or nuclear magnetic resonance (NMR); Wiechert, 2001; Nielsen, 2003; Zamboni *et al*, 2009; Niedenführ *et al*, 2015). ^13^C‐MFA resolves the activity of biochemical reactions through computational modeling which can differentiate between parallel pathways and determine bidirectional fluxes (mass exchange of reactions that proceed forwards and backwards at the same time; Sonntag *et al*, 1993; Wiechert & de Graaf, 1997).

Besides central C metabolism, nitrogen (N) metabolism plays a key role, not only in amino acid and nucleotide metabolism, but also in the synthesis of many cofactors (Chubukov *et al*, 2014). In many microbes including the pathogenic *Mycobacterium tuberculosis*, nitrate acts as a terminal oxygen acceptor in addition to molecular oxygen during hypoxic respiration (Tan *et al*, 2010). Although N fixation and assimilation play a key role in medical research, agriculture, and biotechnology, quantitative insights into N metabolism are currently limited. Consequently, only a few drugs have been developed that target N metabolism (Kurmi & Haigis, 2020). The progress in quantifying N metabolism has been challenging, mostly because there is limited information derived from the isotopic labeling profiles of N versus C atoms. The equivalent of ^13^C‐MFA, namely ^15^N‐MFA therefore needs to involve time‐resolved labeling data and the measurement of intracellular intermediate metabolite concentrations (pool sizes) to deploy the isotopically non‐stationary (INST) MFA framework (Nöh & Wiechert, 2011; Wiechert & Nöh, 2013). This technique was utilized to study ammonium assimilation in *Corynebacterium glutamicum* by quantifying central N fluxes *in vivo* (Tesch *et al*, 1999).

**Figure 1:**
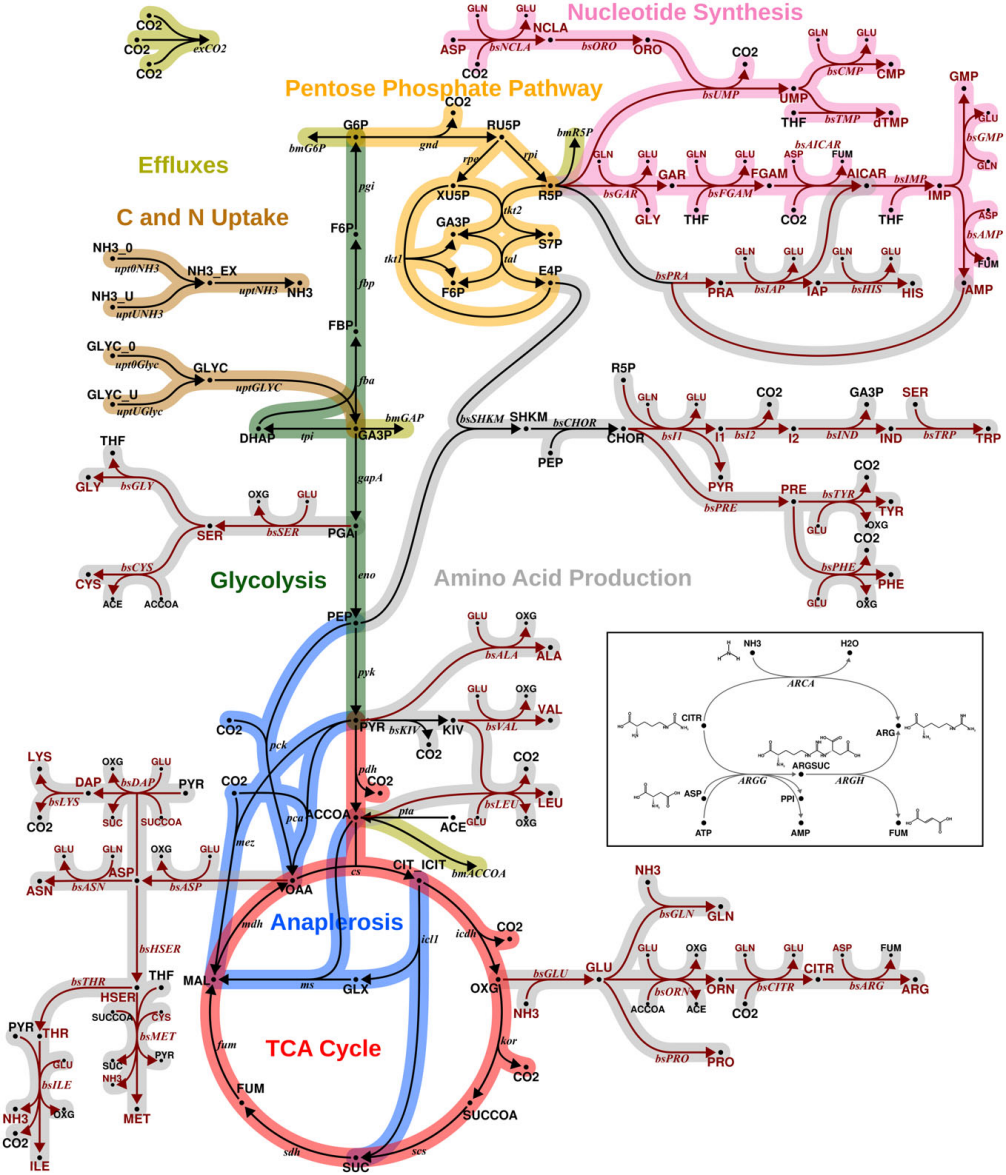
Metabolic network showing carbon and nitrogen metabolism Pathways of carbon and nitrogen metabolism include glycolysis (EMP), pentose phosphate pathway (PPP), tricarboxylic acid (TCA) cycle, and anaplerotic reactions (ANA). Nitrogen source ammonium; carbon source glycerol. Reactions and metabolites involving nitrogen are shown in red. The inset (Appendix Fig S1 enlarged version) shows the last bifurcated step of the arginine biosynthesis, according to the genome‐scale metabolic model sMTB2.0 (López‐Agudelo *et al*, 2020). Citrulline is aminated either by free nitrogen to form arginine (arginine deiminase, *ARCA*), or aspartate is acting as nitrogen donor and arginine is formed via a two‐step reaction with the intermediate argininosuccinate (argininosuccinate synthase (*ARGG*) and argininosuccinate lyase (*ARGH*)). Because the carbon backbone is the same for both branches, ^13^C labeling alone is not able to resolve the fluxes of either of these pathways.

C and N metabolism are interdependent in all organisms (Goel *et al*, 2016; Naliwajski & Skłodowska, 2018). For instance, the tricarboxylic acid cycle (TCA), glycolysis, and pentose phosphate pathway (PPP), which are C based, synthesize amino acids and nucleotides. Biosynthesis of amino acids primarily involves addition of N to the C backbone, requiring reductants and energy, generated primarily from C metabolism (Fig 1). To understand C and N co‐assimilation, a systems‐based analysis is required which includes both central C and N metabolism. The information about ^13^C labeling enrichments of the intermediates in amino acid biosynthesis alone is limited, because such measurements cannot resolve alternative pathways in which no C scrambling occurs, as in the case of arginine biosynthesis pathway (Fig 1, Appendix Fig S1). The key for quantification of C and N fluxes simultaneously is to administer ^13^C and ^15^N isotopic tracers. Using ^13^C_6_^15^N_4_ labeled arginine as the tracer, this co‐labeling strategy was implemented to study arginine metabolism in *Kluyveromyces lactis* (Romagnoli *et al*, 2014); this study showed that ^13^C incorporation into the amino acids proceeded at a slower timescale than the ^15^N‐label. The time‐deconvolution of C and N label incorporation allowed approximation of C and N fluxes using a “staggered” INST ^13^C‐MFA and ^15^N‐MFA approach. However, the efficacy of this approach critically depends on the validity of the underlying timescale separation, which is induced by the efficacious choice of C‐ and N‐labeling sources. Here we have established an approach that simultaneously quantifies C and N metabolic fluxes independently of the co‐labeling strategy applied. Our ^13^C^15^N‐MFA platform specifically tracks C and N atom interconversions throughout the entire metabolic network, without the need to acquire the pool sizes of metabolic intermediates or the deconvolution of ^13^C and ^15^N isotopologues using time‐deconvolution or specialized analytical measurement platforms. We demonstrate the capability of this platform by quantifying intracellular C and N fluxes of the vaccine strain of mycobacteria *Mycobacterium bovis* BCG, as a model for one of the world's most important pathogens, *Mycobacterium tuberculosis* (Mtb) that causes tuberculosis (TB).

Tuberculosis is one of the leading causes of human mortality from a single infectious agent that kills over a million people every year (World Health Organization, 2020). Drug resistance is a major problem affecting TB therapy (Palomino & Martin, 2014; Kurz *et al*, 2016), so new drugs are urgently needed. Measurement of N metabolic fluxes has the potential to identify novel anti‐TB drug targets, but the current progress is hampered by the limitation of tools and technology to measure N along with C fluxes *in vivo*. We previously developed ^13^C‐flux spectral analysis (FSA) and ^15^N‐flux spectral ratio analysis (FSRA) for identifying the probable spectrum of C and N substrates in Mtb *ex vivo* (Beste *et al*, 2013; Borah *et al*, 2019). Using FSRA, we found aspartate, glutamate, and glutamine to be the primary nitrogen sources for intracellular Mtb (Borah *et al*, 2019). Although ^13^C‐FSA and ^15^N‐FSRA provided qualitative conclusions about C and N sources, the available measurements did not allow for flux quantification. Multiple studies have successfully measured C fluxes in Mtb growing as batch cultures (de Carvalho *et al*, 2010; Borah *et al*, 2021). We also applied ^13^C‐MFA to quantify intracellular C fluxes of Mtb and BCG during slow and fast growth in a chemostat (Beste *et al*, 2011). Here, using C and N isotopic co‐labeling, metabolic modeling, and Bayesian statistics we resolved the central C and N co‐metabolism with an increased resolution.

The measurement of N metabolic fluxes, simultaneously with the C fluxes in a principled, system‐wide manner has not been attempted in Mtb or in any organism. The simultaneous measurement of C and N fluxes requires constructing an enlarged metabolic reaction model that describes both central C and N metabolism along with multi‐atom transitions. As a result, the number of unknown flux parameters to be inferred from the experimental measurements, increases significantly. The increase in dimensionality stems primarily from the reaction steps for CN co‐assimilation (mainly reactions catalyzed by transaminases) as these reactions must be modeled bidirectional to adequately describe the co‐labeling enrichments (Wiechert & de Graaf, 1997). In this situation, the ensuing model flexibility renders the standard best‐fit approach, commonly used in ^13^C‐MFA, prone to overfitting (Zamboni *et al*, 2009). This problem is exacerbated when measurements cannot distinguish between labeling contributions stemming from ^13^C and ^15^N tracers, such as data obtained from single quadrupole MS instruments with insufficient mass resolution. To overcome the impediments of current single‐model ^13^C‐MFA approaches, and to provide reliable flux uncertainty estimates under these circumstances, we used a statistically rigorous multi‐model inference approach (Theorell & Nöh, 2020), which we here generalize to ^13^C^15^N‐MFA. Applying Bayesian multi‐model ^13^C^15^N‐MFA to analyze co‐labeling data sets enabled us to measure intracellular metabolic fluxes for the central C and N metabolism in *M. bovis* BCG under steady‐state conditions.

## Results

### Roadmap for Bayesian multi‐model ^13^C^15^N‐metabolic flux analysis

**Figure 2:**
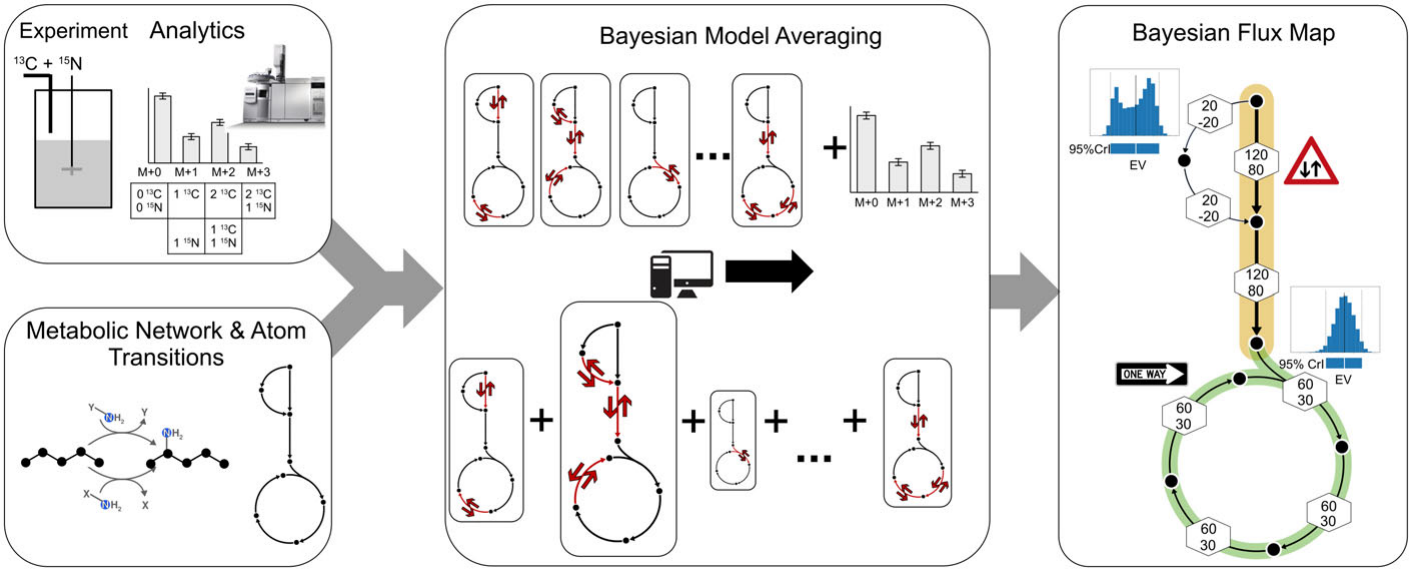
General workflow of ^13^C^15^N‐MFA Labeling data are collected from a ^13^C and ^15^N isotope co‐labeling experiment, performed for a continuous culture in a chemostat setting to achieve metabolic (pseudo) steady state conditions. Cells are harvested at isotopic steady state for the analysis of intracellular metabolites using mass spectrometry along with natural abundance correction. A CN metabolic model is constructed and together with extracellular (uptake, secretion) rates and the labeling data, C and N fluxes are inferred. To this end, a multi‐model inference strategy using Bayesian Model Averaging (BMA) is executed. Here, any specific combination of uni‐ and bidirectional reactions constitutes a model, giving rise to combinatorically many possible model variants. BMA is a statistical procedure to draw inferences from the set of model variants by weighting individual model inferences based on their likelihood to explain the labeling data. The result is the Bayesian flux map that shows the resulting expected values of net fluxes, resulting from marginal posterior probability distributions, along with the probabilities of the reversible reactions to operate uni‐ or bidirectionally.

The ^13^C^15^N‐MFA co‐labeling general workflow is summarized in Fig 2. Cultivation experiments are performed under metabolic (pseudo) steady state conditions, in a C or N limited chemostat. Steady state cultures are switched to media containing ^13^C‐ and ^15^N‐labeled substrates, and samples are drawn after an isotopic steady state labeling is achieved for both C and N. The samples are then analyzed by MS, providing mass isotopomer distributions (MIDs; Nilsson & Jain, 2016). In terms of mass shifts, low‐resolution gas‐chromatography (GC–MS) and liquid‐chromatography mass spectrometry (LC–MS) are often not sufficiently sensitive to distinguish between ^13^C and ^15^N isotopomers, resulting in convoluted univariate (^13^C^15^N) MIDs (Kappelmann *et al*, 2017). For example, in lysine with six C (#C=6) and two N (#N=2) atoms, #C+#N+1= 9 univariate MIDs exist. More advanced analytical platforms such as high‐resolution LC–MS (orbitrap; Nilsson & Jain, 2016), FT‐ICR‐MS (Blank *et al*, 2012), multi‐reflection time‐of‐flight (ToF) MS or tailored derivatization approaches combined with two‐stage LC–MS (Kappelmann *et al*, 2019) can distinguish between ^13^C and ^15^N isotopomers, providing multivariate MIDs. In the lysine example, there are $\left(\#C+1\right)\times \left(\#N+1\right)=21$ multivariate mass isotopomers. Ultra‐high‐resolution orbitrap and FT‐ICR analysers allow resolving the full spectrum. In practice, however, those analytical platforms operate at a trade‐off between resolving power and acquisition speed. Also, low intracellular metabolite concentrations often limit the precise measurements of multivariate MIDs. Therefore, we opted to measure univariate MIDs from the co‐labeling experiment using a single‐quadrupole GC–MS system, which is a robust and often used analytical device for MID analysis (Zamboni *et al*, 2009; Sundqvist *et al*, 2022). Although we focus on MS as mainstream analytics, our workflow is transferable and equally applicable to other analytical platforms such as for NMR delivering heteronuclear NMR moieties (Borkum *et al*, 2017), or a combination of MS and NMR measurements.

Mass isotopomer distributions corrected for natural abundance, along with the extracellular rates and biomass proportions, are incorporated into a metabolic network model that precisely specifies the transition of C and N atoms throughout the intracellular reactions. Tracking N in addition to C requires not only an extension of C mappings by N mappings and the addition of the reactions of nitrogen metabolism, but also requires a refined formulation of biosynthesis reactions that are usually lumped in ^13^C‐MFA (an example is shown in Appendix Fig S1). These extensions enable inference of C and N fluxes from the co‐labeling data. In addition to the transition network, information on the mass exchange between intermediates of these biosynthetic reactions, that is, whether the mass flow through these reversible reactions is unidirectional or bidirectional is required (Wiechert & de Graaf, 1997). For instance, transaminases are suspected to operate at near thermodynamic equilibrium, but quantitative evidence regarding their activity in most biological systems including Mtb are largely missing (Grotkjær *et al*, 2004). As such all transaminase catalyzed biochemical reactions carrying CN fluxes should be considered potentially bidirectional (Wiechert, 2007), implying that they are characterized by two flux parameters, a net and a (typically not well determinable) labeling exchange flux, instead of a unidirectional reaction, which is described by a net flux only. Consequently, this introduces additional model flexibility that challenges the identification of flux parameters in the CN model, because it renders the model susceptible to overfitting. A general solution for dealing with model under‐determinacy in a statistically rigorous manner, while making as few assumptions as possible about the model formulation, has been proposed within the statistical framework of Bayesian Model Averaging (BMA; Hoeting *et al*, 1999). Technically, when applied to ^13^C^15^N‐MFA, BMA determines the probability distributions of net fluxes $\left(v\right)$ given the data $\left(D\right)$, in the Bayesian paradigm expressed as $p\left(v,|,D\right)$, by averaging the flux posterior probabilities over all possible models ($M_{i},i=1,\ldots N$), weighted by the model probability in view of the data $p\left(M_{i}|D\right)$:

(1) $$p\left(v|D\right)=\sum_{i=1}^{N}p\left(v_{M_{i}}|M_{i},D\right)∙p\left(M_{i}|D\right)$$

Here, models $M_{i}$ are structural variants that differ in their bidirectionality setting and, hence, number of flux parameters ($v_{M_{i}}$). Equation (Equation 1) is solved computationally by using a recently developed tailored Markov chain Monte Carlo (MCMC) approach (Theorell & Nöh, 2020; see Materials and Methods for details). This results in so‐called posterior probability distributions for the net fluxes, as well as the probabilities of reversible reactions being uni‐ or bidirectional. The marginal posterior probability distributions provide credible intervals (CrI) and expected values (EV) for the net fluxes. In addition, two‐dimensional marginal posterior probability distributions give insights into possibly non‐linear net flux correlations. Finally, the flux estimation outcome, derived under consideration of data and model formulation uncertainty, is visually summarized in a Bayesian flux map (Fig 2). It should be noted that all traditional flux maps, including those in our previous work (Beste *et al*, 2011), instead consider data uncertainty only and report maximum likelihood‐based point estimates and confidence intervals, whereas flux correlations are exclusive to the Bayesian framework.

### BMA‐based ^13^C^15^N‐MFA validates and refines carbon fluxes in mycobacteria and reveals new insights into the anaplerotic node

We applied the ^13^C^15^N‐MFA workflow to the mycobacterial model system *M. bovis* BCG. The experimental conditions were comparable to those described in Beste *et al* (2011). *M. bovis* BCG was cultivated in continuous culture with glycerol and ammonium chloride as sole C and N sources respectively at a dilution rate of 0.03 h^−1^ (Tables 1 and EV1, Appendix Fig S2A–C). Cultures were grown with 12.5% [^13^C_3_]‐glycerol (GLYC) and 20% [^15^N_1_]‐ammonium chloride (NH_4_Cl) until an isotopic steady state was reached, as confirmed by GC–MS of amino acids (Appendix Fig S2C). Harvested samples were analyzed using GC–MS, providing univariate MID measurements (MIDs that do not distinguish between ^13^C and ^15^N isotopomers) of 15 proteinogenic amino acids.

**Table 1:** Chemostat growth parameters of *Mycobacterium bovis* BCG continuous cultures. The measurements were done for chemostat cultures at metabolic and isotopic steady state. Measurements are mean ± SD from two independent chemostat cultures each with three to four technical replicates.

	Specific consumption/production rate
Glycerol	0.81 ± 0.28 mmol g biomass^−1^ h^−1^
NH_4_Cl	0.84 ± 0.11 mmol g biomass^−1^ h^−1^
Dry weight	1.41 ± 0.12 g l^−1^
Dilution rate (fixed)	0.03 h^−1^

**Figure 3:**
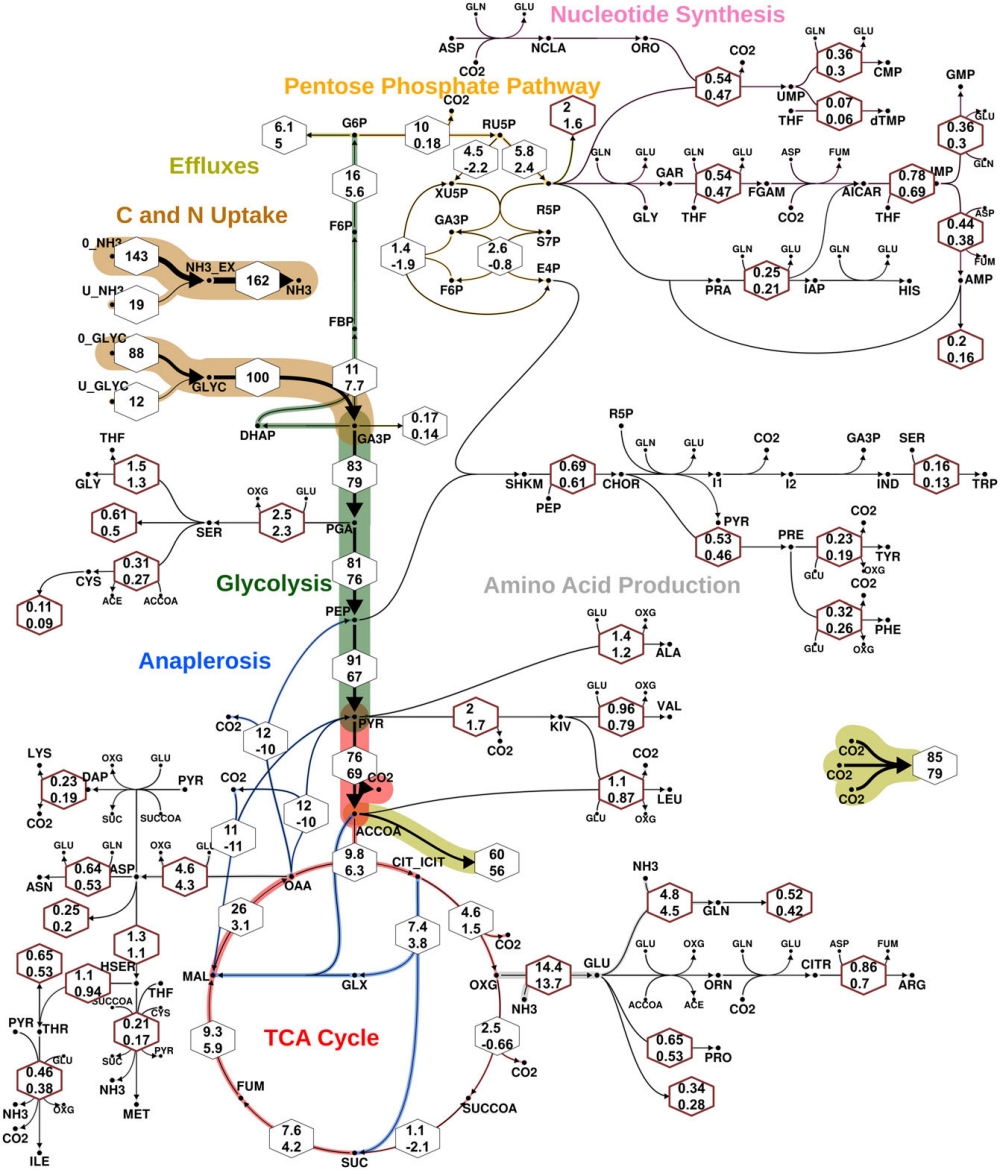
Bayesian flux map for BCG growing at 0.03 h^−1^ inferred with ^13^C^15^N‐MFA The line strengths code for the expected values of the net flux marginal posterior probability distributions (Appendix Fig S3). Their associated 95% credible intervals (CrIs) are given in hexagons, where thin black and thick dark red borders indicate reactions that involve carbon‐ or nitrogen‐only and mixed carbon and nitrogen transfer, respectively. Values are given relative to the glycerol uptake flux (set to 100). The associated absolute net flux CrIs are provided in Fig 4. The nominal reaction direction, indicated by the arrowhead, is given according to a positive EV. Color indicates pathway association of the reactions. Associated probabilities of reversible reactions being bidirectional are found in Appendix Fig S7.

**Figure 4:**
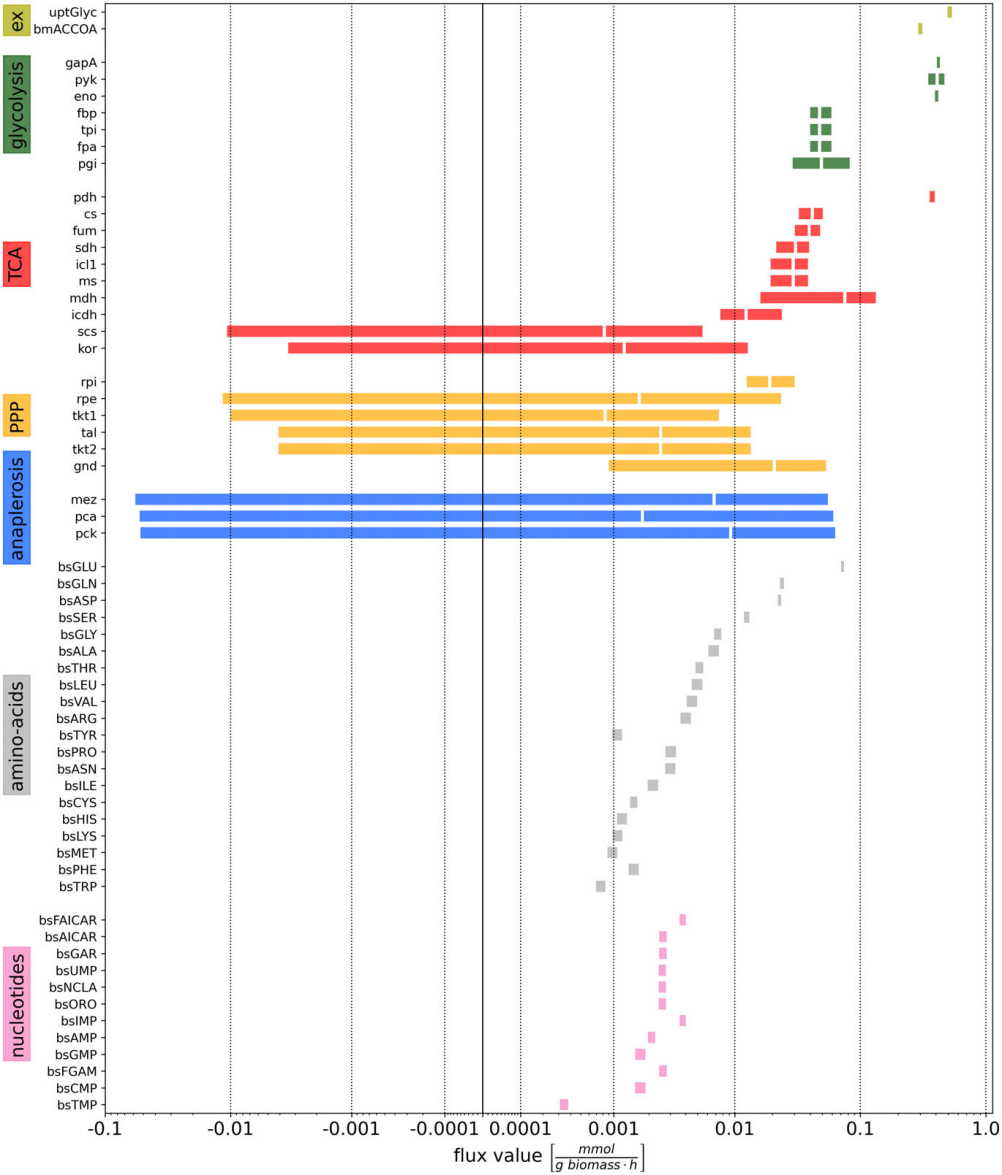
Credible intervals and expected values of absolute fluxes for BCG growing at 0.03 h^−1^ inferred with ^13^C^15^N‐MFA Colors indicate pathways and colored bars specify 95% credible intervals (CrI) for net fluxes, with inscribed white line indicating the expected value (in case of very narrow CrIs are not displayed). Notice that the flux values are bi‐symmetrically log‐transformed (Beau & Webber, 2013), which visually stretches CrIs of fluxes near 0. Although the anaplerotic fluxes *mez*, *pca* and *pck* have CrIs ranging from −0.06 mmol g biomass^−1^ h^−1^ to 0.06 mmol g biomass^−1^ h^−1^, they are strongly correlated as shown in Fig 5A–C.Source data are available online for this figure.

The resulting Bayesian flux map is shown in Fig 3 with net fluxes relative to the glycerol (GLYC) uptake rate, while the CrIs of the absolute fluxes are given in Fig 4 (see also Appendix Fig S3 for the marginal flux posterior probability distributions). The primary C metabolic route is directed from GLYC over lower glycolysis towards lipid and fatty acid synthesis (via the acetyl‐CoA drain flux). While glycolytic fluxes were the highest, the TCA cycle, PPP, and anaplerotic fluxes are significantly lower. The fluxes through the decarboxylating arm of the TCA cycle, oxoglutarate ferredoxin oxidoreductase (*kor*) and succinyl‐CoA synthetase (*scs*) reactions are reduced. Rv2454c‐Rv2455c encoding *kor* and Rv0951‐Rv0952 encoding *scs* in Mtb participates in the decarboxylating steps of the TCA cycle, which are bypassed using glyoxylate shunt, instantiating the GAS pathway (Beste *et al*, 2011). The GAS pathway utilizes the glyoxylate shunt and anaplerotic reaction for oxidation of pyruvate. The operation of this pathway has been measured using our current BMA analysis, consistent with the results derived previously using ^13^C‐MFA (Beste *et al*, 2011). The net C fluxes through the upper glycolysis, the TCA and anaplerosis for BCG at a growth rate of 0.03 h^−1^ measured using Bayesian ^13^C^15^N‐MFA are qualitatively very similar to those derived by traditional ^13^C‐MFA in our previous study (Beste *et al*, 2011; Appendix Fig S4).

There are, however, also differences in the flux maps of central C metabolism. Our previous flux map showed cyclic fluxes around fructose 6‐phosphate (F6P) node, involving the fluxes *pgi*, *gnd*, *tkt2*, *tal*, and *tkt1*. This cycle is also confirmed by the present ^13^C^15^N‐MFA study, but with fluxes lower than previously reported. Notice, however, that the previously reported fluxes of the PPP represent best‐fit values that were fixed in the statistical analysis due to their non‐identifiability, and thus lacking uncertainty estimates. Accentuated differences are found for the *pdh* flux in lower glycolysis. We attribute this discrepancy to the differences in the experimental setup between the two studies: in this study, tyloxapol was used as dispersant in the medium as replacement for tween‐80 or oleic acid, which was a medium component in our previous study, and is known to be a carbon source for mycobacteria (Pietersen *et al*, 2020). By comparing the two flux analyses using tween or tyloxapol in the medium, we concluded that only discrepancies in lower glycolytic fluxes, but no “global” effects on central carbon metabolism were found under the investigated conditions.

As in our former ^13^C‐MFA analysis (22), net fluxes of the anaplerotic reactions, pyruvate carboxylase (*pca*), PEP carboxykinase (*pck*), and malic enzyme (*mez*), could not be resolved as seen from their large CrIs in Fig 4. This is a consequence of the cyclic network topology of the anaplerotic node (Kappelmann *et al*, 2016). In our previous analysis *pca* and *mez* were aggregated by lumping oxaloacetate (OAA) and malate (MAL). In this study, the three anaplerotic reactions were modeled in detail without imposing any assumption on their reaction directionalities or bidirectionalities.

**Figure 5:**
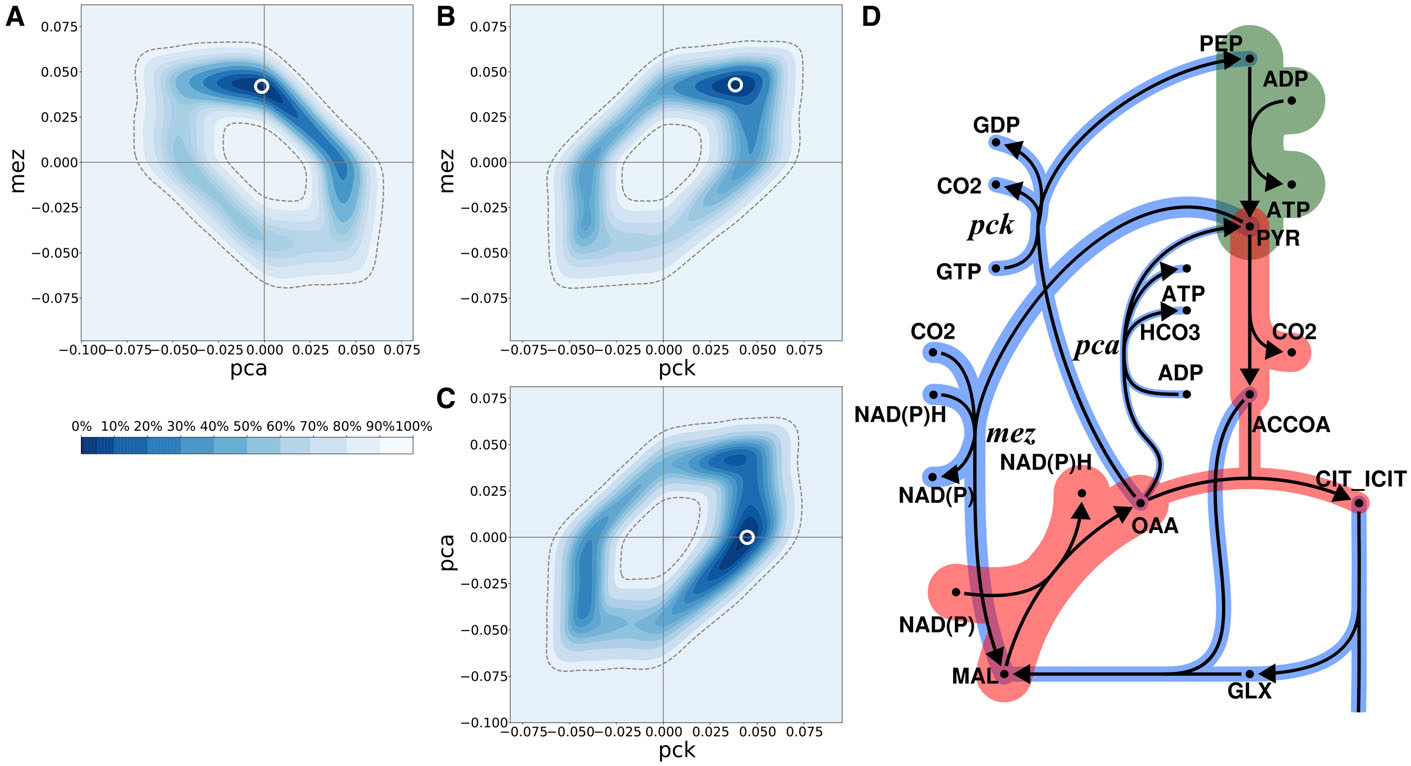
Marginal posterior probabilities and operation modes of the anaplerotic node A–C
Joint marginal flux posterior distributions for the three anaplerotic reactions pyruvate carboxylase (*pca*), PEP carboxykinase (*pck*), and malic enzyme (*mez*). Darker (lighter) colors indicate regions of higher (lower) flux probability given the labeling data. The dashed lines indicate the 95% credible region, horizontal/vertical lines indicate zero fluxes. For *mez* vs *pca*, *mez* vs *pck*, and *pca* vs *pck* fluxes ring‐like shapes emerge, revealing complex correlations between the fluxes. Apparently, the reaction pairs are unlikely to both carry zero flux. The symbol “o” marks the most likely flux values given the ^13^C^15^N data set.D
The metabolic network of the anaplerotic node, and the most likely fluxes labeled by “o” in A, B, C. Starting from phosphoenolpyruvic acid, carbon flows via *pyk* and *mez* to malate, which is then transformed via *mdh* and *pck* back to phosphoenolpyruvic acid. See Appendix Fig S5 for an extended version of the figure, and the interpretation of further, possible flux constellations.

Despite limited information in the co‐labeling data for identification of the directionalities of the anaplerotic reactions, we were able to refine the resolution of the flux map and limit the absolute flux values to a range of ±0.06 mmol g biomass^−1^ h^−1^ (approx. 10% of glycerol uptake). However, the two‐dimensional (2D) marginal posterior probability distributions shown in Fig 5, and in the extended version Appendix Fig S5, show that the information contained in the ^13^C^15^N data set effectively narrows down the joint space of possible values considerably further to concise, ring‐like regions within the flux space. With these inferences, many flux constellations are ruled out. For instance, given the data set at hand, it is very unlikely that the reaction pairs *pca* and *pck*, *pca* and *mez*, or *pck* and *mez*, carry zero fluxes. This insight, which was not implemented in the flux model *a priori*, is indeed experimentally supported: the mentioned anaplerotic reactions catalyze gluconeogenic utilization of carbons to replenish the TCA cycle and operation of these fluxes has been demonstrated to be important for the survival of Mtb (Basu *et al*, 2018). In the previous analysis in Beste *et al* (2011), we were unable to derive such precise information due to model simplifications enforced by flux non‐identifiabilities (Beste *et al*, 2011).

From the 2D marginal posterior flux probability distributions in Fig 5A–C we further see that the flux pairs *pck/mez* and *pck/pca* are largely positively correlated, meaning that a larger value of one flux implies a larger value of the other. In contrast, *pca* and *mez* are largely negatively correlated. Thus, at least one of the three anaplerotic reactions is operating in gluconeogenetic direction. In a larger context, Appendix Fig S5 shows that *mdh* and *pyk* are highly correlated with *mez* and *pck*, respectively.

Beyond such qualitative assessment of the flux inferences, Fig 5 shows the most likely flux operation modes, that is, flux constellations that are located in the highest probability region. Here, we discuss the most likely flux mode, indicated by a circle (o) in Fig 5A–C. In this mode, *mez* is 0.05 mmol g biomass^−1^ h^−1^ and *pca* is net positive, while the *pck* net flux is zero. Together, this operation mode, shown in Fig 5D, poses a futile cycle: starting from phosphoenolpyruvic acid, carbon flows via *pyk* and *mez* to form malate, which is then transformed gluconeogenetically via *mdh* and *pck* back to phosphoenolpyruvic acid. Alternatively, the slightly less likely, flux operation modes for the anaplerotic node are detailed in Appendix Fig S5.

In conclusion, the C flux profile for BCG growing at a faster growth rate (0.03 h^−1^) inferred here by BMA‐based ^13^C^15^N‐MFA represents an independent replication of the previous ^13^C‐MFA‐derived C fluxes. The C flux maps derived from the two labeling approaches and the two MFA platforms are comparable, while our current Bayesian approach imposes fewer modeling assumptions, provides more reliable flux uncertainties, and delivers an increased flux resolution, in particular for the previously non‐inferable anaplerotic node in BCG. The results from our Bayesian analysis provide explanations of the analyzed data set, which narrow down the range of likely fluxes (Fig 4) to concise possible functional anaplerotic flux modes (Fig 5).

### ^13^C^15^N‐MFA quantifies C and N‐fluxes

In addition to the C fluxes discussed in the previous section, ^13^C^15^N‐MFA together with the extended scope of the network allowed us, for the first time, to quantify C and N net fluxes in *M. bovis* BCG from a co‐labeling data set, that is, reaction fluxes in the amino acid and nucleotide biosynthetic pathways. These fluxes are shown in Fig 3 (hexagons with thick red borders) and Fig 4 in relative and absolute numbers. The largest biosynthetic flux involving C and N is *bsGLU* (glutamate dehydrogenase), with an expected value of 0.072 mmol g biomass^−1^ h^−1^ (95% CrI: 0.071–0.074 mmol g biomass^−1^ h^−1^), followed by *bsGLN* (glutamine synthetase), *bsASP* (aspartate transaminase) and *bsSER* (serine deaminase). The fluxes of the remaining amino acid and nucleotide synthesis including adenosine monophosphate (AMP), guanosine monophosphate (GMP), cytidine monophosphate (CMP), inosine monophosphate (IMP) and uridine monophosphate (UMP), are one order of magnitude lower, with values rendering the proportion to which they contribute to biomass formation (Fig 4).

When interpreting fluxes determined using ^13^C^15^N‐MFA (Fig 4) or comparing them with any previously reported flux values, it is important to realize that fluxes always must be interpreted in relation to the actual material flows they represent. For example, it has been previously reported (Beste *et al*, 2011), that alanine aminotransferase (*bsALA*) is the largest biosynthetic net carbon flux, being one order of magnitude larger than glutamate dehydrogenase (*bsGLU*). In this study, we found that *bsALA* (95% CrI: 0.006–0.007 mmol g biomass^−1^ h^−1^) is one order of magnitude *lower* than *bsGLU* (95% CrI: 0.071–0.074 mmol g biomass^−1^ h^−1^). This apparent discrepancy is explained by the nitrogen flux that is not within the scope of ^13^C‐MFA. More precisely, conventional ^13^C‐MFA can only give a lower bound on the nitrogen requirement of an amino acid by summing up the (C) fluxes of reactions that incorporate nitrogen from the donor. For example, adding the fluxes to alanine (ALA), asparagine (ASN), glutamate, glutamine (GLN), leucine (LEU), lysine (LYS), phenylalanine (PHE), serine (SER), tyrosine (TYR), tryptophan (TRP), valine (VAL), ornithine (ORN), arginine (ARG), and the contributions to nucleotide synthesis, gives a value of 0.061 mmol g biomass^−1^ h^−1^, which underestimates the *bsGLU* net CN flux expected value of 0.071 mmol g biomass^−1^ h^−1^ derived by ^13^C^15^N‐MFA in this study.

**Figure 6:**
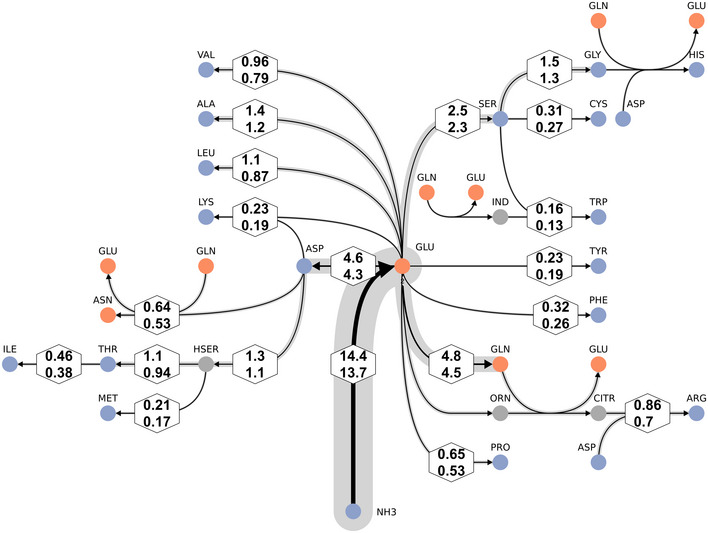
Bayesian N‐flux map for BCG growing at 0.03 h^−1^ inferred with ^13^C^15^N‐MFA GLU‐centric view with focus on amino acids. The line strengths code for the expected values of the net flux posterior probability distributions. Lower and upper limits of their associated 95% credible intervals (CrIs) are given in hexagons. To maintain compatibility with Fig 3, values are given relative to the glycerol uptake flux (set to 100). Glutamate (GLU) is the primary nitrogen donor for most amino acids, but also glutamine (GLN), aspartate (ASP), and serine (SER) are important nitrogen hubs.

**Figure 7:**
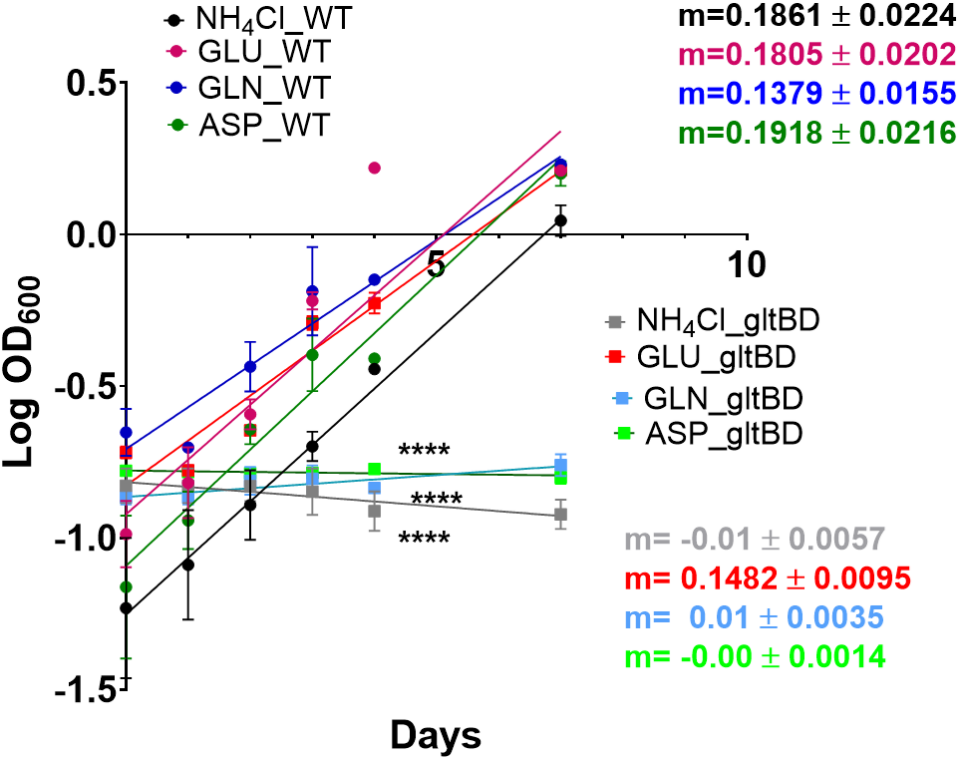
Growth of wild type and gltBD mutant BCG strain on minimal medium containing glycerol, GLU, ASP, GLN and NH_4_Cl A positive slope m > 0 indicates exponential growth. A negative slope (m < 0) indicates no exponential growth. Values are mean ± s.e.m (*n* = 3 independent biological replicates). * indicates statistically significant deviation of the slope from 0; ****P < 0.00001. Source data are available online for this figure.

**Figure 8:**
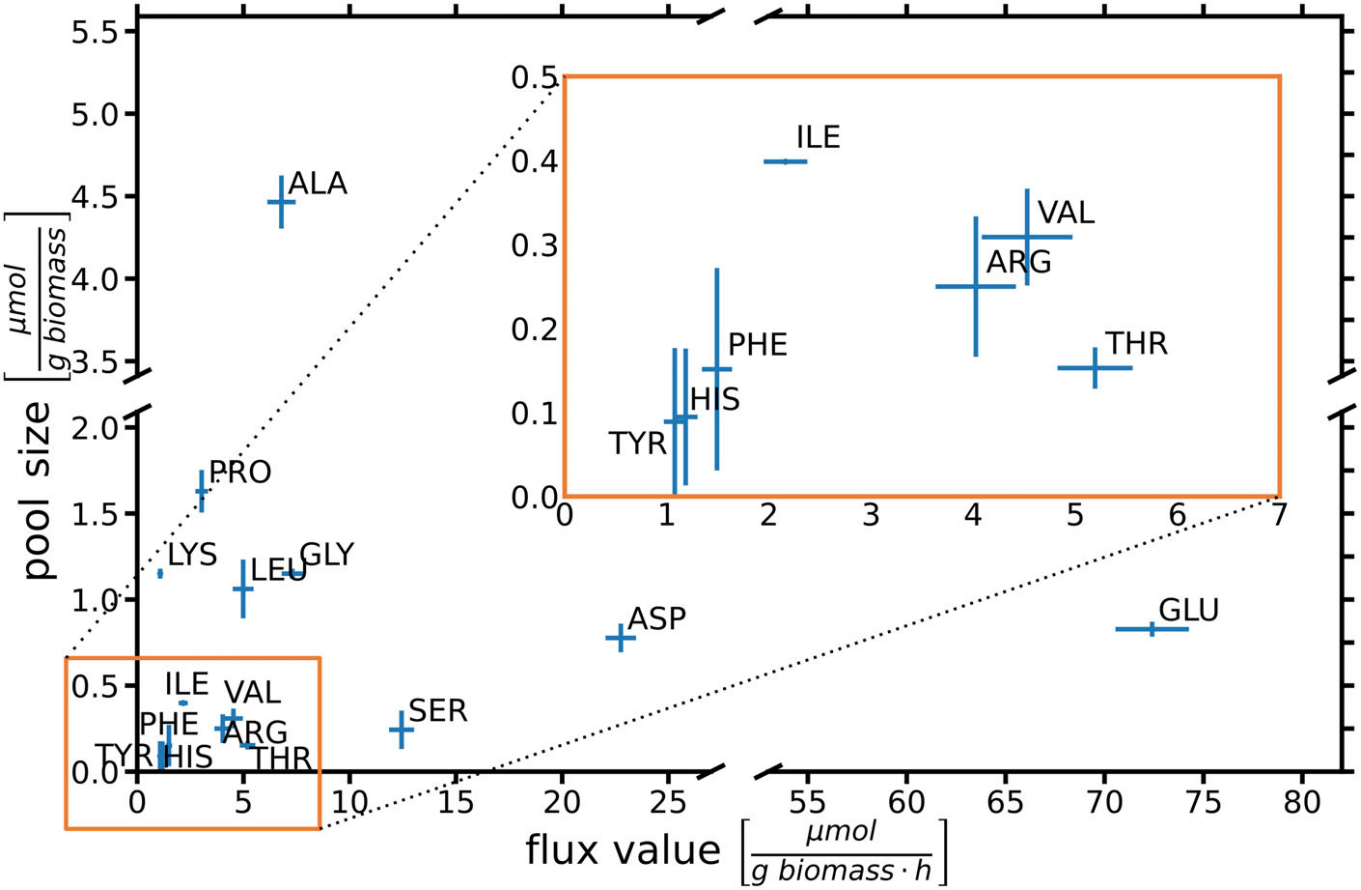
Pool sizes vs. biosynthetic CN fluxes of protein‐derived amino acids Values are mean ± S.D. (*n* = 12 measurements; 4 biological replicates, 3 technical replicate each) for pool size measurements and 95% CrIs for flux values. Source data are available online for this figure.

The strength of ^13^C^15^N‐MFA is that it provides quantitative flux measurements for N metabolism, along with the C fluxes. The N flux map shown in Fig 6 highlights the central role of glutamate (GLU) as an N donor. To a lesser extent, glutamine (GLN) and aspartate (ASP) are also N donors, which explains their significantly higher CN flux as compared to the C fluxes previously reported (Beste *et al*, 2011). Glutamate donates its N to other amino acids through various transamination reactions. The centrality of this node for N assimilation was experimentally confirmed by examining substrate utilization of a glutamate auxotroph of *M. bovis* BCG with a transposon mutation in gltBD, a gene encoding glutamine oxoglutarate aminotransferase (GOGAT) that catalyzes the synthesis of GLU from OXG and GLN (Viljoen *et al*, 2013). Whereas the wild type *M. bovis* BCG strain could grow with GLYC as sole C and NH_3_, ASP, GLU and GLN as sole N sources (slope m > 0), the gltBD mutant was able to grow only on glutamate as the N source (Fig 7). The CN‐fluxes for GLU and ASP‐derived amino acids including ASN, threonine (THR), isoleucine (ILE), LYS, methionine (MET), ARG and PRO and phosphoglyceric acid (PGA)/phosphoenlypuruvate (PEP) derived ALA, SER, cysteine (CYS), VAL and leucine (LEU) are higher than the PPP‐derived amino acids (Fig 4). Interestingly, ALA had the largest pool size amongst the protein‐derived amino acids, but the alanine aminotransferase flux was by far not the highest. Also, for the other amino acids, no direct relationship between pool sizes and fluxes is apparent, highlighting that pool size and fluxes are complementary measures of metabolism (Fig 8; Appendix Fig S6; Buescher *et al*, 2015; Wang *et al*, 2020; Wiechert & Nöh, 2021).

### Bayesian multi‐model ^13^C^15^N‐MFA uncovers reversibility of glycine biosynthesis and unidirectionality of leucine and isoleucine biosynthesis

GLU and GLN serve as the main amino donors for the synthesis of other amino acids in BCG (Figs 4 and 6). To meet the demands for protein, RNA and DNA synthesis, the N net flux is principally directed towards amination. However, the reversibility of the enzymes responsible for catalyzing transaminases provide cells with the ability to adapt rapidly to environmental conditions such as changes in N availability. Indeed, fine‐tuning the activities of transaminases to modulate C flux has been demonstrated in the CHO eukaryotic cell line (Wahrheit *et al*, 2014). This means that while the biosynthetic net reaction flux is directed towards amination, a simultaneous forward and backward (bidirectional) flux is possibly occurring *in vivo*.

Due to the lack of evidence about the reversibility of mycobacterial transaminases, we set the transaminase reactions in the model as potentially bidirectional to capture the flexibility of the amination network and to avoid the risk of any bias that would arise from setting a transamination reaction erroneously unidirectional. All transamination reactions were modeled as bidirectional reaction steps, resulting in a flux estimation problem with 86 degrees of freedom (71 fluxes and 15 measurement group scales) to be recovered from 144 measurements (109 univariate MIDs and 34 rate and biomass efflux measurements). Here, BMA was used to minimize overfitting (see methods and protocols). This technique explores the space of all possible combinations of uni‐ and bidirectional reactions (each combination codes for an alternate model) and weights each model variant by its ability to explain the labeling data (see also Fig 2). In contrast to the conventionally used single‐model approaches, BMA‐based ^13^C^15^N‐MFA enables not only the rigorous statistical inference of net fluxes, and thereby reaction directionality, but also determines reaction bidirectionality (Theorell & Nöh, 2020). The information contained in the given labeling data did not allow all bidirectional reactions of biosynthesis to be classified as either bi‐ or unidirectional (Appendix Fig S7) with distinct exceptions: glycine hydroxymethyltransferase (*bsGLY*) that catalyzes glycine (GLY) biosynthesis was determined to be bidirectional with 100 ± 0% probability. In contrast, serine deaminase, isoleucine, and leucine transaminase (*bsSER*, *bsILE* and *bsLEU*) were found to be unidirectional with high probability (98.6 ± 0.3% probability, 99.8 ± 0.05%, and 100 ± 0% probability, respectively).

## Discussion

The interplay between C and N assimilation and dissimilation is required to sustain cell metabolism and function. To date, the knowledge about C and N co‐assimilation and these fluxes within a cell remains limited. For measuring metabolic fluxes, isotope tracing studies in combination with computational modeling is a gold standard. Recently, multiple stable isotopic tracers have been used to measure C and N enrichments and to derive insights into N assimilation in eukaryotic systems including yeast, plant, and human cancer cells (Blank *et al*, 2012; Nilsson & Jain, 2016). Two pioneering studies determined metabolic fluxes of the GS‐GOGAT pathway in *C. glutamicum* by INST ^15^N‐MFA (Tesch *et al*, 1999) and the CN fluxes for arginine metabolism in *K. lactis* (Romagnoli *et al*, 2014). Both these studies had limitations; the former was limited to measuring N fluxes in a small sub‐network, and the latter ^13^C^15^N‐flux analysis relied on a tailored labeling strategy that exploited the fact that the ^13^C tracer was incorporated faster into metabolites than the ^15^N tracer.

Here we generalized the well‐established methodology of ^13^C‐MFA towards ^13^C^15^N‐MFA for deriving system‐wide C and N fluxes from a combination of isotopic co‐labeling experiments and comprehensive multi‐atom transition modeling. The generalization is paired with the use of BMA, which provided rigorous quantification of metabolic fluxes with low‐ and medium‐resolution MS data by measuring intracellular metabolic fluxes through the central C and N network of *M. bovis* BCG. The application of BMA‐based ^13^C^15^N‐MFA instead of the conventional single‐model approach was motivated by three factors:

- The Bayesian framework allows approximating the full posterior probability distributions of the net fluxes from which accurate nonlinear credible intervals (1D) and credibility regions (2D) are derived.
- The multi‐model approach captures flux parameter and model formulation uncertainty, thereby providing consolidated flux uncertainty quantification.
- BMA‐based ^13^C^15^N‐MFA reliably evaluates low‐demand co‐labeling data, without the need of settling for potentially biasing model reductions.

These key advantages of BMA‐based ^13^C^15^N‐MFA, together with the extension of scope and refinement in terms of the C and N metabolic network, enable us to estimate C and N fluxes in one consolidated approach and provide reliable estimates for the uncertainties in the parameters. This enabled the rigorous statistical assessment of all reactions, even those with unknown bidirectionality and cyclic network structures that were previously unresolvable.

Our previous work using ^13^C‐MFA demonstrated conserved carbon flux distributions between BCG and Mtb during growth on glycerol demonstrating metabolic consistency between the two mycobacterial systems (Beste *et al*, 2011). Therefore, our BMA‐based C and N fluxes in BCG are relevant to studying the pathogenic Mtb. We and others have demonstrated that the TB pathogen co‐metabolizes multiple host nutrients during infection (de Carvalho *et al*, 2010; Beste *et al*, 2013; Gouzy *et al*, 2013; Bottai *et al*, 2014; Borah *et al*, 2019). Co‐catabolism of C sources by mycobacteria and the associated metabolic regulations have been previously demonstrated by multiple studies using transcriptional, metabolomics and genetic approaches (Pandey & Sassetti, 2008; de Carvalho *et al*, 2010; Rhee *et al*, 2011; Eoh & Rhee, 2014; Bi *et al*, 2017; Rizvi *et al*, 2019; Serafini *et al*, 2019, 42–47). Although multiple studies have explored the C and N metabolism of Mtb *in vitro* and within *ex vivo* and *in vivo* animal models, the information about C and N fluxes and the key metabolic steps that could be targeted for drug development remain limited. Here we used [^13^C_3_]‐glycerol and [^15^N_1_]‐ammonium chloride dual isotopic labeling of steady state BCG cultures to measure intracellular C and N fluxes. We provide the first comprehensive intracellular C and N flux distributions in a biological system. A comparison with our previously published ^13^C‐MFA in Mtb and *M. bovis* BCG (Beste *et al*, 2011) showed a broad agreement for the net fluxes of central C metabolism. C fluxes through glycolysis and PPP including phosphoglucose isomerase (*pgi*), fructose bisphosphatase (*fbp*), aldolase (*fba*), glucose‐6‐phosphate dehydrogenase/glucolactonase (*gnd*), transketolase (*tkt1*, *tkt2*) and transaldolase (*tal*) were not quantified accurately with our previous ^13^C‐MFA and recent ^13^C^15^N‐MFA study because PPP reactions lack of sufficient labeling information from proteinogenic amino acids. Incorporating labeling information from glycogen, ribose moiety of DNA, glucosamine moiety from peptidoglycan and lipopolysaccharides, such as suggested by Kohlstedt and Wittmann (2019) is a promising approach to improve flux precision in this area of metabolism. The fluxes of the three anaplerotic enzymes pyruvate carboxylase (*pca*), PEP carboxy kinase (*pck*), and malic enzyme (*mez*), which play an important role in metabolism connecting catabolism and anabolism with energy generation, are notoriously difficult to determine in general (Basu *et al*, 2018). Nonetheless, remarkably the information contained in the low demand CN co‐labeling data was sufficient, using BMA‐based ^13^C^15^N‐MFA, to narrow down the anaplerotic net fluxes, to reveal precise net flux correlations, and to uncover conclusive modes possibly active operating in mycobacteria. The Bayesian flux map is consistent with our previous results showing non‐identifiability of individual anaplerotic fluxes (Beste *et al*, 2011); however, the co‐labeling data in this study inform about distinct likely flux couplings of *pca*, *pck*, and *mez*. From our analysis we conclude that at least one of the three reactions is operating in the gluconeogenic direction, and it is unlikely that two or more of the associated fluxes are zero at the same time.

We also measured fluxes to amino acid and nucleotide (purine and pyrimidine) biosynthesis, providing novel information, which cannot be deduced from our former ^13^C‐MFA. We previously identified ASP, GLU and GLN as primary C and N sources for Mtb in human host macrophages (Beste *et al*, 2013; Borah *et al*, 2019). Here we quantified the fluxes for the biosynthesis of these amino acids. We identified glutamate biosynthesis *bsGLU* as the primary node for CN flux. This is consistent with the finding that ASP, GLN, and NH_4_^+^ as sole N sources in Roisin's minimal medium failed to rescue the growth of a *M. bovis* BCG mutant lacking functional *glt*BD gene and thereby lacking *de novo* glutamate synthesis (Viljoen *et al*, 2013; Gallant *et al*, 2016). GLU is a well‐established N source for *in vitro* and intracellular growth of mycobacteria. GLU metabolism is also crucial in mycobacteria to resist acidic and nitric oxide stress inside macrophages (Viljoen *et al*, 2013; Gallant *et al*, 2016) and is therefore a prime metabolic and regulatory node. Furthermore, we were able to quantify bidirectionality probabilities. Specifically, glycine hydroxymethyltransferase (*bsGLY*) was found to be bidirectional, whereas serine deaminase (*bsSER*) and isoleucine and leucine transaminase (*bsILE* and *bsLEU*) were determined to be unidirectional. C and N metabolic profiling has been attempted using isotopic labeling by other studies. Blank *et al* (2012) investigated simultaneous C and N incorporation in *Saccharomyces cerevisiae* administering two different isotopic substrates ^13^C‐glucose and ^15^N‐alanine and measured dual label incorporation in amino acids using FT‐ICR‐MS. Our study demonstrates that conventional GC–MS, which is the traditional workhorse for isotopomer analysis of amino acids, in combination with dual labeling experiments is useful for robust and reproducible flux quantification by BMA‐based ^13^C^15^N‐MFA. Our CN metabolic network is currently limited to amino acid and nucleotide biosynthesis, but there are further scopes for extension of this model through addition of biocomponents such as co‐factors NADH and NADPH, and lipids that requires CN assimilation and dissimilation.

In summary, we have developed Bayesian ^13^C^15^N‐MFA, a powerful tool for simultaneous quantification of intracellular C and N metabolic fluxes in a living system. We applied it to *M. bovis* BCG. BMA‐based ^13^C^15^N‐MFA identified glutamate as the central node of nitrogen metabolism, revealed the most likely operational modes of the anaplerotic fluxes, and resolved the uni/bidirectionalities of glycine, serine, isoleucine, and leucine biosynthesis. Our ^13^C^15^N‐MFA workflow described here is applicable to any CN isotopic co‐labeling experiment, and the computational platform developed in this work allows analyses of low demand, low‐ and medium‐resolution MS data to provide rigorous, consolidated quantification of C and N metabolic fluxes in any biological system.

## Materials and Methods

### Reagents and Tools table

**Table:** 

Reagent/resource	Reference or source	Identifier or catalog
**Bacterial strains**		
*Mycobacterium bovis* BCG Pasteur	American Type Culture Collection	ATCC 35748
*M. bovis BCG gltBD transposon mutant*	VIB – Vlaams Instituut voor Biotechnologie	(*BCG_3922c TnInsertion‐8654*)
**Reagents**		
Middlebrook 7H11	Merck Life Science	M0428‐500G
Middlebrook 7H9	Merck Life Science	M0178‐500G
OADC	Becton Dickenson	212351 (4312351)
Glycerol	Merck Life Science	G7893‐1 L
Tyloxapol	Merck Life Science	T8761‐50G
Brain heart infusion agar	Merck Life Science	70138‐500G
Rosins minimal media	Beste *et al* (2011)	N/A
[^13^C_3_] glycerol, 99% purity	CK Isotopes	CLM‐1510‐5
[^15^N_1_] NH_4_Cl, 98% atom purity	Merck Life Science	299251
2 liter bioreactor	Electrolab	Fermac 310/60
Peristaltic pump	Rainin Rabbit Plus	N/A
Gas analyzer	Electrolab	Fermac 368
Glycerol assay kit	Merck Life Science	MAK117
Ammonia assay kit	Merck Life Science	MAK310
BCA assay kit	Merck Life Science	71285‐M
Ziehl neelsen stain	Merck Life Science	1.09215
**Mass spectrometry equipment**		
tert‐butyldimethyl silyl chloride (TBDMSCl)	Merck Life Science	00942‐10ML
N‐Methyl‐N‐(trimethylsilyl)trifluoroacetamide, MSTFA	Merck Life Science	M‐132
GC–MS 7890‐5795	Agilent	Borah *et al* (2019)
VF‐5 ms 30 m × 0.25 mm × 0.25 μm + 10 m EZ‐Guard	Agilent	CP9013
Dionex UltiMate system (HPLC)	Thermo Fisher Scientific	3000 RSLC
C18 and ZIC‐pHILIC column (150 mm × 4.6 mm, 5 μm column)	Merck Sequant	150461
Thermo Orbitrap Q Exactive Plus	Thermo Fisher Scientific	N/A
**Software**		
Chemstation	Agilent	N/A
GraphPad Prism 8.0	GraphPad software	N/A
Omix v.2.0.7	Omix Visualization GmbH & Co.KG, Lennestadt/Germany	N/A
13CFLUX2 v2.2	Weitzel *et al* (2013)	N/A
HOPS v2.0.0	Jadebeck *et al* (2021)	N/A

### Methods and Protocols

#### Media preparation

Middlebrook 7H11 agar and Middlebrook 7H9 broth containing 5% (vol/vol) oleic acid‐albumin‐dextrose‐catalase enrichment medium supplement (OADC) and 0.5% (vol/vol) glycerol were used to grow cultures from frozen stocks and for counting the numbers of culturable bacteria in chemostat samples. Brain heart infusion agar was used to assess culture purity. For cultivation of *M. bovis* BCG in the bioreactor, roisins minimal medium with composition‐ KH_2_PO_4_, 1 g l^−1^; Na_2_HPO_4_, 2.5 g l^−1^; NH_4_Cl, 5.9 g l^−1^; K_2_SO_4_, 2 g l^−1^; ZnCl_2_, 0.08 mg l^−1^; FeCl_3_, 0.4 mg l^−1^; CuCl_2_, 0.02 mg l^−1^; MnCl_2_, 0.02 mg l^−1^; Na_2_B_4_O_7_, 0.02 mg l^−1^; NH_4_MoO_4_, 0.02 mg l^−1^; MgCl_2_, 0.0476 g l^−1^; CaCl_2_, 0.055 g l^−1^; Tyloxapol, 01% (v/v); Glycerol, 0.5% (v/v).

#### Isotopic labeling of *M. bovis* BCG batch cultures

We performed single‐ and co‐labeling (with two isotopic substrates) of *M. bovis* BCG batch cultures with (a) [^13^C_3_] glycerol (12.5%) + unlabeled NH_4_Cl, (b) unlabeled glycerol + [^15^N_1_] NH_4_Cl (20%), and (c) [^13^C_3_] glycerol (12.5%) + [^15^N_1_] NH_4_Cl (20%) substrates. Samples for mass isotopomer analysis were harvested at approximately same time points in the late exponential growth phase. Mass isotopomers were corrected for natural abundance of unlabeled atoms. Univariate C and N, as well as multivariate CN mass isotopomer data of selected proteinogenic amino acids are shown in Appendix Fig S8.

#### *M. bovis* chemostat cultures in the bioreactor

*Mycobacterium bovis* BCG strain was cultured in a 2 liter bioreactor under growth conditions (Table EV1). Cultures were grown as batch for 7 days. Continuous cultures were grown under chemostat conditions at a growth rate of 0.03 h^−1^ maintained by the media flow rate (Beste *et al*, 2011). Media was pumped into the chemostat using a peristaltic pump. Cultures were grown for 3–4 volume changes in the unlabeled media to assure a metabolic steady‐state before introducing isotopically labeled media. [^13^C_3_] glycerol (12.5%) and [^15^N_1_] NH_4_Cl (20%) were the carbon and nitrogen isotopically labeled substrates in the media. Isotopic stationary state was assessed by measuring % label in the proteinogenic amino acids of cultures drawn at different times during label feed (Appendix Fig S2C).

#### Chemostat measurements and culture analyses

Cultures were monitored every day to check for contamination by plating on BHI agar media and ziehl neelsen stain. Cultures from chemostat were regularly sampled for measuring OD and colony forming units (Beste *et al*, 2011). Carbon‐di‐oxide production from the cultures was monitored using Gas analyzer. The supernatant was collected, filtered using 0.22 μm unit filters and used for substrate consumption and product excretion analyses. To measure the glycerol uptake, the amounts of glycerol in the supernatant and fresh medium was measured using glycerol assay kit by a coupled enzyme assay involving glycerol kinase and glycerol phosphate oxidase, resulting in a colorimetric (570 nm) product, proportional to the glycerol present. To measure NH_4_Cl uptake, the amounts of NH_4_Cl were measured using ammonia assay kit by reaction of ammonia present in the samples involving L‐glutamate dehydrogenase activity. Dry weight of the cells was measured by centrifuging cultures, drying the cell pellet using freeze dryer and weighing the cells. The dried pellet was used for protein analysis using Bicinchoninic Acid Kit for Protein Determination.

#### Metabolite extraction

Labeled chemostat cultures were quenched using methanol:chloroform:water (2:1:2) extraction. Briefly, cultures were filtered using membrane Filter, 0.22 μm pore size, filter apparatus and the filter was immersed into methanol:chloroform, mixed, incubated on ice for 30 min and water was added, followed by centrifuging at room temperature for triphasic metabolite separation. The upper phase was collected separately and dried and used for mass spectrometry analysis. The lower and intermediate phase were mixed into one phase by addition of one more volume of methanol and chloroform and centrifuged for 30 min at room temperature. Supernatant was discarded, the pellet was hydrolyzed in 6 M hydrochloric acid for 24 h at 100°C and the hydrolysate was dried for mass spectrometry analysis.

#### Mass spectrometry analysis of amino acids

Dried upper phase was derivatized using N‐Methyl‐N‐(trimethylsilyl)trifluoroacetamide, MSTFA and dried hydrolysate were derivatized using tert‐Butyldimethylsilyl chloride (TBDMSCl) were analyzed using GC–MS (7890‐5795 system; Borah *et al*, 2019). Mass spectra were baseline corrected using MetAlign and mass isotopomer distribution (MID) data were extracted using the chemstation software. Identification of metabolites was done using NIST databases, literatures, and qualifier masses. Average ^13^C^15^N fractional abundances were calculated from two independent chemostat cultivations (three‐ or four technical replicates each) and quantitation of metabolite pool sizes was done using calibration curves (Borah *et al*, 2019). Further confirmation of ^13^C and ^15^N dual labeling in the amino acids were done using LC–MS orbitrap (Appendix Fig S9), and, in addition, ^13^C, ^15^N and ^13^C^15^N batch labeling experiments (Appendix Fig S8). Briefly, hydrophilic interaction liquid chromatography (HILIC) was carried out on a Dionex UltiMate 3000 RSLC system using a C18 and ZIC‐pHILIC column (150 mm × 4.6 mm, 5 μm column). The column was maintained at 30°C and samples were eluted with a linear gradient (20 mM ammonium carbonate in water, A and acetonitrile, B) over 26 min at a flow rate of 0.3 ml/min. The injection volume was 10 μl and samples were maintained at 4°C prior to injection. For the MS analysis, a Thermo Orbitrap Q Exactive Plus was operated in polarity switching mode and the MS settings were used with resolution 70000, AGC 106, m/z range 70–1,400, sheath gas 40, Auxiliary gas 5, sweep gas 1, probe temperature 150°C and capillary temperature 275°C. For positive mode ionization: source voltage +4.5 kV, capillary voltage +50 V, tube voltage +70 kV, skimmer voltage +20 V. For negative mode ionization: source voltage‐3.5 kV, capillary voltage‐50 V, tube voltage‐70 V, skimmer voltage‐20 V. The data shown in Appendix Fig S9 is a mass spectrum showing the multivariate ^13^C and ^15^N species identification for alanine.

#### ^13^C^15^N‐Metabolic flux analysis

Metabolic network model: The metabolic model *M. bovis* BCG used for the analysis was constructed using the network editor Omix v.2.0.7 (Omix Visualization GmbH & Co. KG, Lennestadt/Germany; Droste *et al*, 2013), according to the protocol described in Nöh *et al* (2015), based on the GSMN‐TB genome‐scale model of *M. tuberculosis* (Beste *et al*, 2007). The constructed model includes reactions of glycolysis, the PPP, the TCA cycle, anaplerosis, nucleotide and amino acid biosynthesis (Fig 1). Uptake reactions were considered for GLYC and ammonium chloride (NH_4_Cl). All biosynthesis pathway fluxes relevant for growth of *M. bovis* are modeled as effluxes, whose values represent their share in the biomass composition (Beste *et al*, 2005). Reactions were classified to be unidirectional (labeling exchange flux = 0), bidirectional (labeling exchange flux > 0) or unknown, that is, potentially bidirectional (labeling exchange flux ≥ 0). Here, all reactions are considered potentially bidirectional, unless evidence was available that the reactions operate close or far from thermodynamic equilibrium under *in vivo* conditions (Table EV2). In particular, transaminases are modeled potentially bidirectional. Each potentially bidirectional reaction is given a 50:50 probability to be unidirectional or bidirectional, giving rise to combinatorially many structurally different model variants. The probabilistic view enables inference of the probability for a reaction being uni‐ or bidirectional from the given data. Each metabolic reaction was supplemented with carbon and nitrogen atom transitions, following the InChI atom enumeration scheme (Heller *et al*, 2013). Carbon symmetries of succinate (SUCC), fumarate (FUM), and diaminopimelic acid (DAP) were accounted for by the formulation appropriate label scrambling reactions. In total, the *M. bovis* BCG model consists of 248 metabolites (121 balanced intracellular and 127 unbalanced extracellular pools) and 184 metabolic reactions (149 unidirectional, 35 bidirectional). The most comprehensive model in the model set has 71 independent flux parameters (36 net and 35 exchange fluxes). The corresponding CN atom transition network model is formulated in the standardized document format for isotope‐based MFA, FluxML v3 (Beyß *et al*, 2019), and is also found in Table EV2.

Measurement models: In total 30 biomass effluxes were considered as measurements that were either obtained from biomass hydrolysates or calculated from intermediates (Beste *et al*, 2011) and supplied with Gaussian error of 5%. Uptake rates for glycerol and NH_4_Cl were fixed for the analysis. Labeling measurements of 15 amino acids, were corrected for the effect of natural abundant isotopes (Millard *et al*, 2012), adding up to 109 univariate MIDs (Table EV3). The associated measurement errors were derived from up to 8 replicates (two independent chemostats, each with two‐four replicate measurements). The complete measurement specification is given in the FluxML model file (Table EV1).

Labeling system & simulation: The ^13^C‐MFA high‐performance simulator 13CFLUX2 v2.2 (Weitzel *et al*, 2013) was extended to simulate ^13^C^15^N isotopologues. Briefly, the essential cumomer framework (Weitzel *et al*, 2007) was generalized from single‐atom to multi‐atom species labeling systems. The resulting balance equations consist of a set of sparse linear equation systems that were solved sequentially with on‐the‐fly algebraic simplification guaranteeing numerical stability, accuracy, and efficiency. The resulting reduced co‐labeling system has a state‐space dimension of 471 (a reduction of a factor of 2,563 compared to the full co‐labeling system).

Flux inference with Bayesian Model Averaging: Instead of conventional optimization‐based single‐model flux inference, in this work metabolic fluxes were estimated using a Bayesian multi‐model approach. More precisely, net fluxes and reaction bidirectionalities were inferred simultaneously by employing BMA, implemented using a tailored MCMC approach (Theorell & Nöh, 2020). Herein, 13CFLUX2 was used for likelihood computation. For speed, sampling algorithms implemented in the C++ library HOPS v2.0.0 (Jadebeck *et al*, 2021) were employed after suitable preprocessing using Polyround v0.1.8 (Theorell *et al*, 2022). The code to reproduce the analysis is available at https://github.com/JuBiotech/Supplement‐to_Borah‐et‐al.‐MSB‐2023 (https://doi.org/10.5281/zenodo.7506282). Parallel tempering with dynamic temperature selection was applied to sample from potentially multi‐modal distributions. In total, 10 parallel chains were run from independent starting points, where per chain 15 × 10^6^ forward simulations were performed. For each chain, the first 5 × 10^6^ samples were discarded (burn‐in). Proper convergence of the MCMC runs was diagnosed by measuring the Potential Scale Reduction Factor (PSRF; Gelman & Rubin, 1992) on a subset of samples, where all but every 2,000^th^ sample was disregarded (thinning). Computations were run on a workstation with dual Intel(R) Xeon(R) Gold CPU (61300 @ 2.8 GHz). PSRF, mixing plots in parameter as well as model spaces are provided in Table EV4, Appendix Figs S10 and S11.

Statistical evaluation: From the MCMC results, posterior probability distributions $p\left(v|D\right)$ for the net fluxes v were derived, according to (Equation 1). Formally, the posterior probability of a model $M_{i}$ out of the model set $\left\{M_{i}|\;i=1,\ldots ,N\right\}$ is given by

(2) $$p\left(M_{i}|D\right)=\frac{p\left(D|M_{i}\right)∙p\left(M_{i}\right)}{\sum_{k=1}^{N}p\left(D|M_{k}\right)∙p\left(M_{k}\right)}$$

where $p\left(M_{i}\right)$, the prior knowledge about model $M_{i}$, is considered equal for all models. $p\left(D|M_{i}\right)$ represents the high dimensional marginalization integral over all possible fluxes the model $M_{i}$ can take. 95% CrIs were determined for each net flux (i.e., the range that contains the flux with a probability of 95% in view of the data), discarding the upper and lower 2.5% of the values. In addition, as point estimator, the expected value for the net flux is reported. The marginal distributions for the net fluxes are provided in Appendix Fig S3. For each reversible reaction, the posterior probability of the reaction being bidirectional was determined, according to (Equation 2), as ratio of models sampled with the reaction set bidirectional divided by the total number of sampled models (Appendix Fig S7). The 2D credible regions were determined using the *plot_kde* method of *arviz* (Kumar *et al*, 2019).

Prediction of labeling data: A calibrated ^13^C^15^N model is useful to predict labeling data that could have been observed in isotopic labeling experiments. The Bayesian formulation facilitates the construction of posterior predictive mass isotopomers, where the inferred net fluxes v are provided by the posterior distribution $p\left(v|D\right)$. To this end, in total 50,000 representative samples for the fluxes were taken from t $p\left(v|D\right)$ and mass isotopomer data mimicking two single (12% [^13^C_3_] glycerol +0% [^15^N_1_] ammonium chloride, 0% [^13^C_3_] glycerol +12% [^15^N_1_] ammonium chloride), and the co‐labeling (12% [^13^C_3_] glycerol +12% [^15^N_1_] ammonium chloride) experiments were simulated. Results are shown in Appendix Fig S12. ^15^N_1_ ammonium chloride labeling strategy is uninformative about the fluxes, as it is solely determined by the applied N labeling percentage. For the co‐labeling experiment with dual species ^13^C and ^15^N labeling, simulations and experimental data are comparable, implying that inferences are plausible with respect to the fluxes.

#### Statistical analysis

Students *t*‐test, analysis of variance (ANOVA), and linear regression analyses were performed in GraphPad Prism 8.0.

## Author contributions

**Khushboo Borah Slater:** Formal analysis; funding acquisition; investigation; visualization; methodology; writing – original draft; writing – review and editing. **Martin Beyß:** Software; formal analysis; investigation; visualization; methodology; writing – original draft; writing – review and editing. **Ye Xu:** Writing – review and editing. **Jim Barber:** Writing – review and editing. **Catia Costa:** Writing – review and editing. **Jane Newcombe:** Writing – review and editing. **Axel Theorell:** Software; writing – review and editing. **Melanie J Bailey:** Resources; funding acquisition; writing – review and editing. **Dany J V Beste:** Writing – review and editing. **Johnjoe McFadden:** Conceptualization; resources; supervision; funding acquisition; investigation; writing – original draft; writing – review and editing. **Katharina Nöh:** Conceptualization; resources; software; supervision; investigation; methodology; writing – original draft; writing – review and editing.

## Disclosure and competing interests statement

The authors declare that they have no conflict of interest.

## Supporting information



Appendix

Table EV1

Table EV2

Table EV3

Table EV4

Dataset EV1

Source Data for Figure 4

Source Data for Figure 7

Source Data for Figure 8

## Data Availability

^13^C‐^15^N atom transition model and ^13^C‐^15^N mass spectrometry dataset from the chemostat experiments are provided in Tables EV2 and EV3. Random seeds and scripts for reproduction of BMA‐based flux inferences are available at https://github.com/JuBiotech/Supplement‐to_Borah‐et‐al.‐MSB‐2023 and are also archived on Zenodo (https://doi.org/10.5281/zenodo.7506282). This repository contains: compiled BMA extension to 13CFLUX2 (ver >= 2.3), script with the used parameters for x3cflux‐bma (as used in the study), random seeds for the MCMC chains (for reproduction of results), FluxML model file of M. bovis BCG containing reactions, CN transitions, constraints, and data.
