# Supplementary material for: One‐shot 13C15N‐metabolic flux analysis for simultaneous quantification of carbon and nitrogen flux
Source: Mol Syst Biol. 2023 Jan 27;19(3):e11099. doi: 10.15252/msb.202211099 (PMC9996240; doi:10.15252/msb.202211099)
Supplement: Supplementary file 1 — Appendix [file MSB-19-e11099-s001.pdf]

Appendix for

One-shot $^{13}C^{15}N$-metabolic flux analysis for simultaneous quantification of carbon and nitrogen flux

Authors

Khushboo Borah Slater[1,¶], Martin Beyß[2,3,¶], Ye Xu[1], Jim Barber[1], Catia Costa[4], Jane Newcombe[1], Axel Theorell[2,5], Melanie J Bailey[4], Dany JV Beste[1], Johnjoe McFadden[1,¶,*] Katharina Nöh[2, ¶,*]

Affiliations

[1] Faculty of Health and Medical Sciences, University of Surrey, UK, GU2 7XH

[2] Forschungszentrum Jülich GmbH, Institute of Bio- and Geosciences, IBG-1: Biotechnology, 52425 Jülich, Germany

[3] RWTH Aachen University, Computational Systems Biotechnology, 52062 Aachen, Germany

[4] Faculty of Engineering and Physical Sciences, University of Surrey, UK, GU2 7XH

[5] Current address: Computational Systems Biology, ETH Zürich, 4058 Basel, Switzerland

[¶] These authors contributed equally to this work

*Correspondance email : j.mcfadden@surrey.ac.uk; k.noeh@fz-juelich.de

Table of content

Appendix Figure S1: Page 2

Appendix Figure S2: page 3

Appendix Figure S3: Page 4

Appendix Figure S4: page 5

Appendix Figure S5: Page 6

Appendix Figure S6: page 7

Appendix Figure S7: Page 8

Appendix Figure S8: page 9

Appendix Figure S9: Page 10

Appendix Figure S10: page 11

Appendix Figure S11: page 12

Appendix Figure S12: page 13

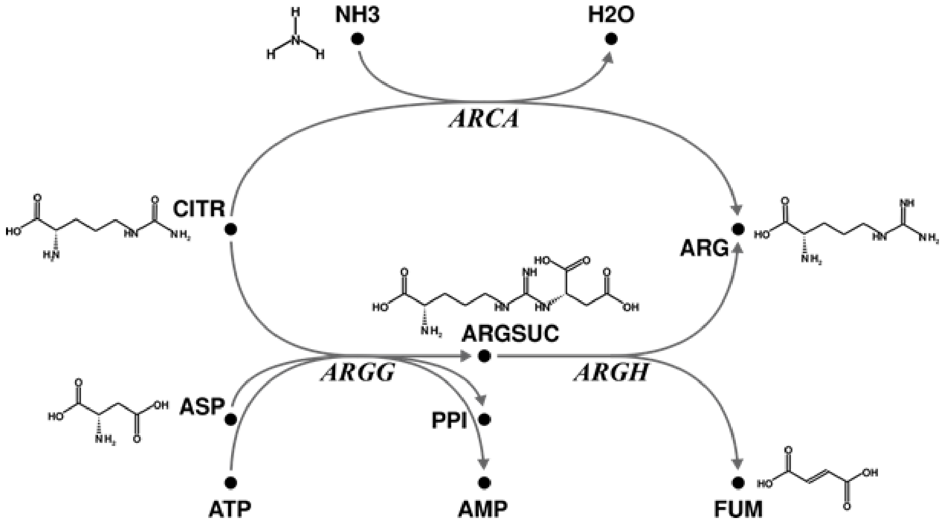

Appendix Figure S1. **Metabolic network showing carbon and nitrogen metabolism.** Pathways show the last bifurcated step of the arginine biosynthesis, according to the genome-scale metabolic model sMTB2.0 (López-Agudelo *et al*, 2020). Citrulline is aminated either by free nitrogen to form arginine (arginine deiminase, ARCA), or aspartate is acting as nitrogen donor and arginine is formed via a two-step reaction with the intermediate argininosuccinate (argininosuccinate synthase (ARGG) and argininosuccinate lyase (ARGH)). Because the carbon backbone is the same for both branches, 13C labelling alone is not able to resolve the fluxes of either of these pathways.

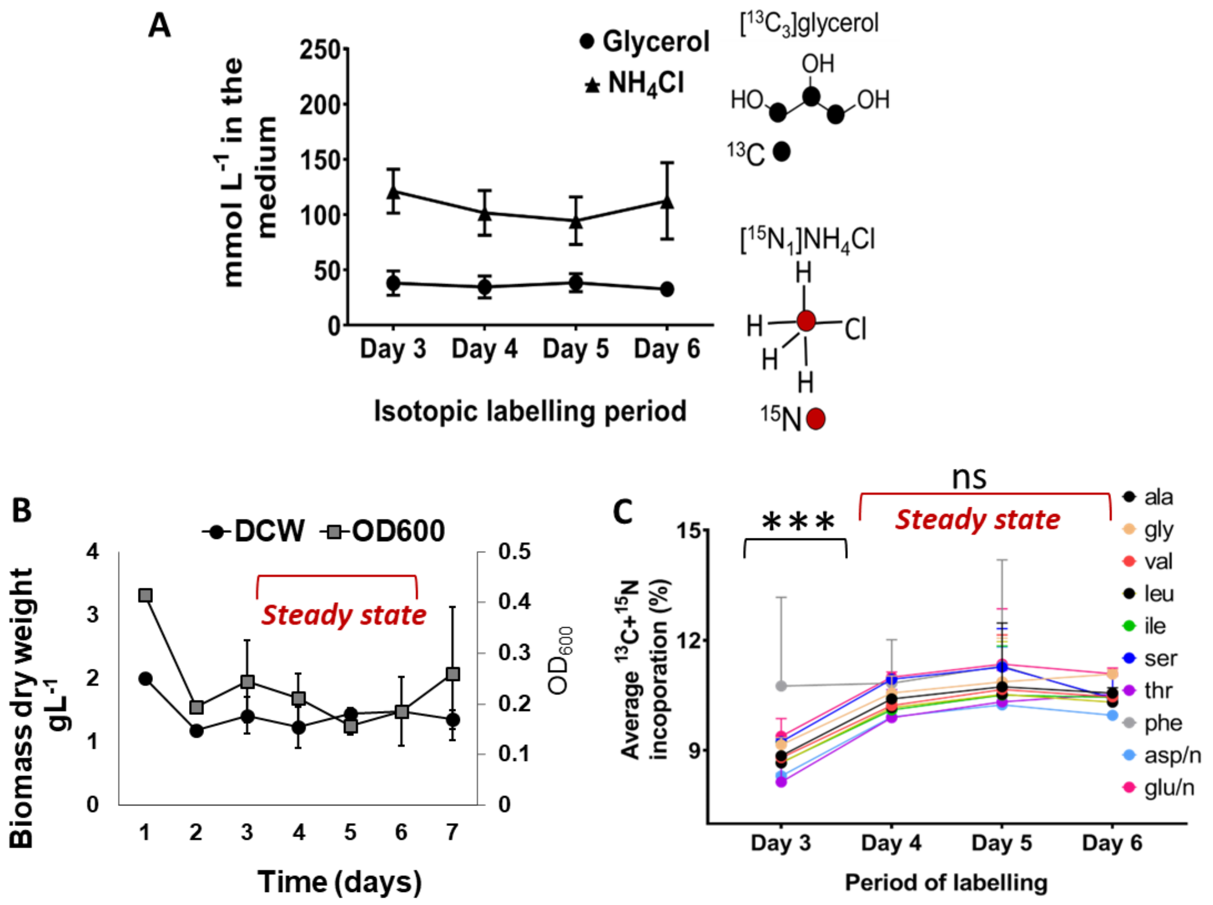

Appendix Figure S2. **$^{13}C^{15}N$ co-labelling of BCG in a chemostat**.
(A) Substrate concentration during the steady state growth of BCG.
(B) Metabolic steady state. Cultures were grown in [$^{13}C_3$]glycerol and [$^{15}N_1$]NH_4Cl isotopic substrates at a growth rate of $0.03h^{-1}$. Cultures were harvested for dry cell weight and OD_{600} measurement over the labelling feed period of 7 days.
(C) Isotopic steady state. Average ^{13}C+^{15}N incorporation (%) in 10 amino acids was measured using GC-MS and was compared between the combinations of (Day 3, Day 4), (Day 4, Day 5), (Day 5, Day 6) using unpaired, parametric two-tailed t test with Welch's correction; ***, $p < 0.0005$; ns, not significant. Multiple t-tests comparing the CN % in each amino acids over four day labelling period showed significantly different measurements of ala (alanine), gly (glycine), ser (serine), thr (threonine), valine (val), leu (leucine), isoleucine (ile), asp/n (aspartate/asparagine), glu/n (glutamate/glutamine) and phe (phenylalanine) in day 3 vs. Day 4. Measurements in Day 3 were significantly different to Day 4, indicating non-steady state isotopic labelling period. Measurements from Day 4 through to Day 6 were no significantly different, indicting steady-state isotopic labelling.
<u>Data information</u>: Values are mean ± s.d. of two independent chemostat cultures and 3-8 replicate measurements.

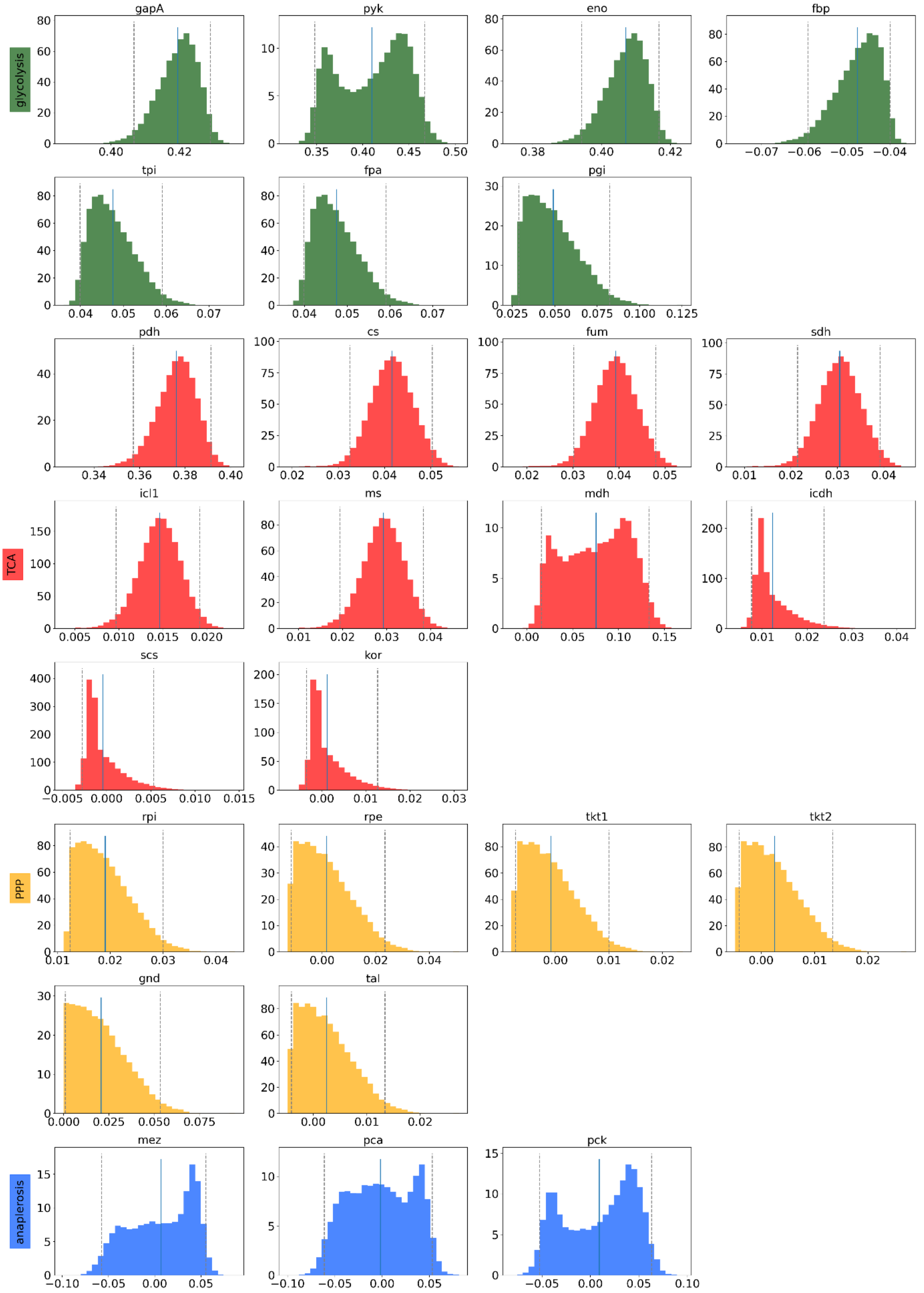

Appendix Figure S3. **Marginal posterior probability density distributions of intracellular net fluxes**.

Dashed lines indicate upper and lower bounds of the 95% credible intervals, and the vertical solid line the expected value. Flux values are given in mmol g biomass^{-1} h^{-1}. Colors are chosen according to the pathway colorings in Figures 1, 3 and 4 in the main text.

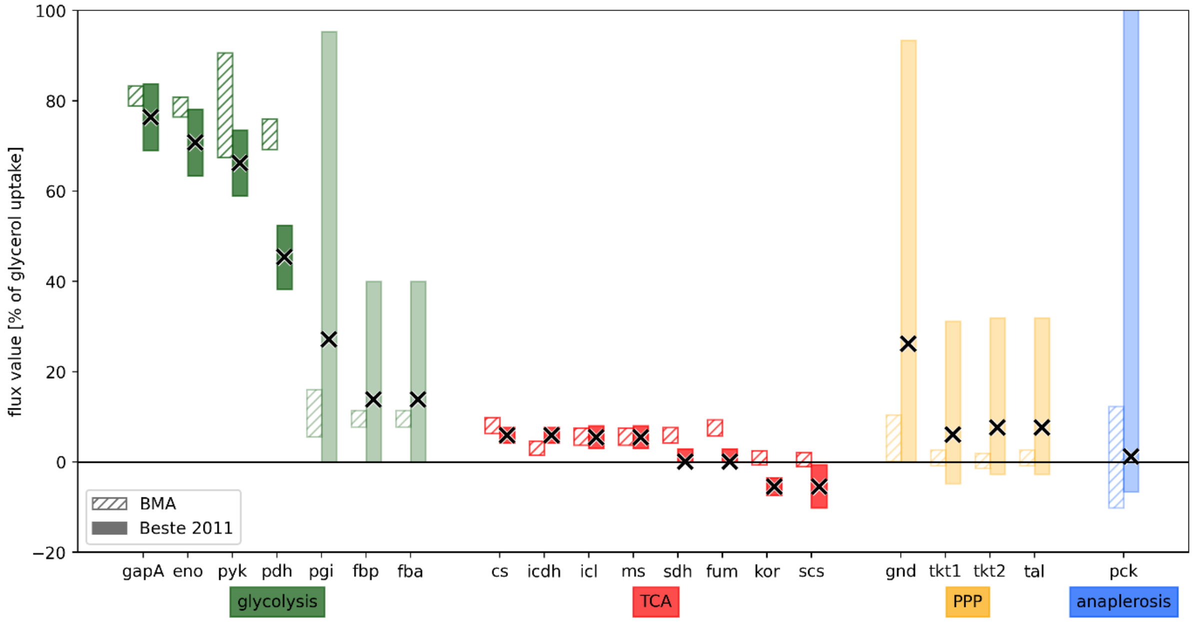

Appendix Figure S4. **Comparison of net carbon fluxes derived with $^{13}C^{15}N$-MFA (BMA, this work) and ^{13}C-MFA (Beste *et al.*, 2011)**.

95% credible intervals (CrI) derived using $^{13}C^{15}N$-MFA of central carbon fluxes are shown in shaded bars. Best-fit carbon fluxes derived using ^{13}C-MFA (indicated by the symbol "x") and their associated 95% confidence intervals (CoI, Fisherian standard deviations) are shown in solid bars. Notice that in the study of Beste et al. (2011) several fluxes were either fixed to their best-fit value prior statistical analysis, therewith lacking CoIs, or were lumped, making a direct comparison impossible. For the previously fixed fluxes the stochiometric bounds are shown in lighter shade (*pgi*, *fpb*, *fba*, *gnd*, *tkt1*, *tkt2*, *tal*, *pck*). Colors are chosen according to the pathway colorings in Figures 1, 3, and 4 in the main text. Differences in profiles occur in fluxes of lower glycolysis (*gapA*, *eno*, *pyk*, *pdh*). Differences in fluxes of *pdh* are explained by the uptake of oleic acid in Beste *et al*. (2011).

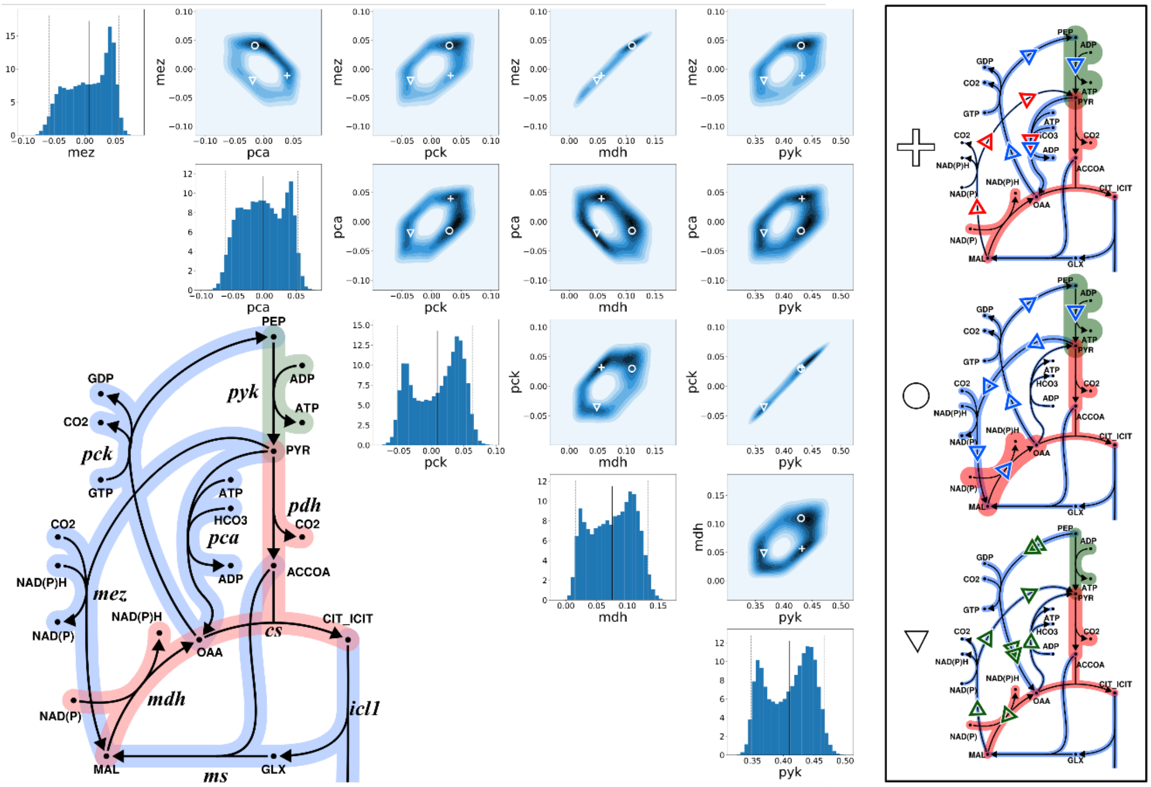

Appendix Figure S5. **Marginal 1D and 2D posterior probabilities and modes of fluxes of the anaplerotic node.**

Metabolic network of the anaplerotic node. Diagonal entries represent 1D posterior probability distributions, off- diagonal entries represent pairwise 2D posterior probability distributions. The 2D posterior distributions can be grouped into three categories: (1) strong linear correlation for *mez* vs *mdh* and *pck* vs *pyk*; (2) a dual positive coupling for the flux pairs *mez* vs *pck*, *mez* vs *pyk*, *pca* vs *pck*, *pca* vs *pyk*, *pck* vs *mdh* and *mdh* vs *pyk*); and (3) a dual negative coupling for *mez* vs *pca* and *pca* vs *mdh*. Fluxes are given in mmol g biomass^{-1} h^{-1}. To better understand the complex coupling of the anaplerotic fluxes, we picked three different likely operational modes, indicated by the symbols (+, □, □). The corresponding flux maps are shown on the right. Roughly, case "+"consists of two parts: the blue arrows show a futile cycle involving *pyk*, *pca* and *pck*, whereas the red arrows show a bypass of *mdh* via *mez* (against the nominal direction) and *pca*. Case "□" represents a futile cycle consisting of *pyk*, *mez*, *mdh* and *pck* as indicated by the blue arrows. Finally, case "□" as indicated by the green arrows consists of two parts, both bypassing *pyk*, either by *pca* and *pck* (both against their nominal direction), or via *pca*, *mdh* and *mez* (all against their nominal direction).

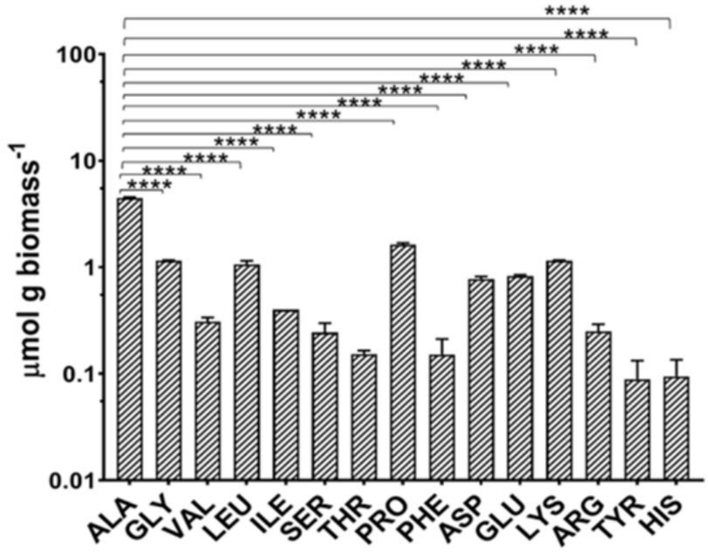

Appendix Figure S6. **Pool sizes of protein-derived amino acids in BCG chemostat cultures**. Values are ± s.d. (n=12 measurements; 4 biological replicates, 3 technical replicate each). Statistical differences were identified using one way ANOVA and tukey's multiple comparisons test with $\alpha \leq 0.05$; ****, $p \leq 0.0001$.

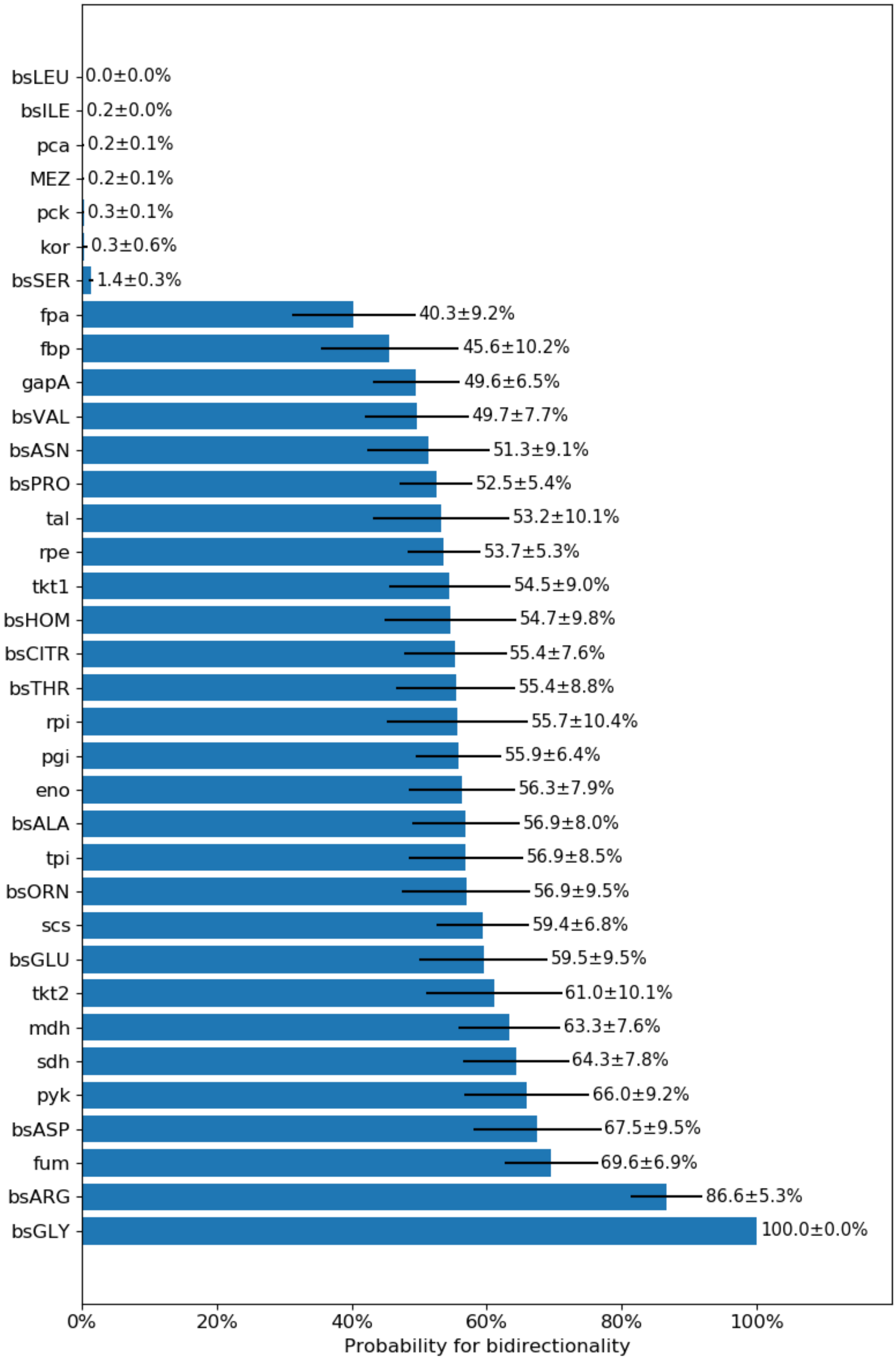

Appendix Figure S7. **Probability of reactions to be bidirectional**.
Bars represent the mean value of 10 independent chains. Standard deviations were derived from 10 replicate chains.

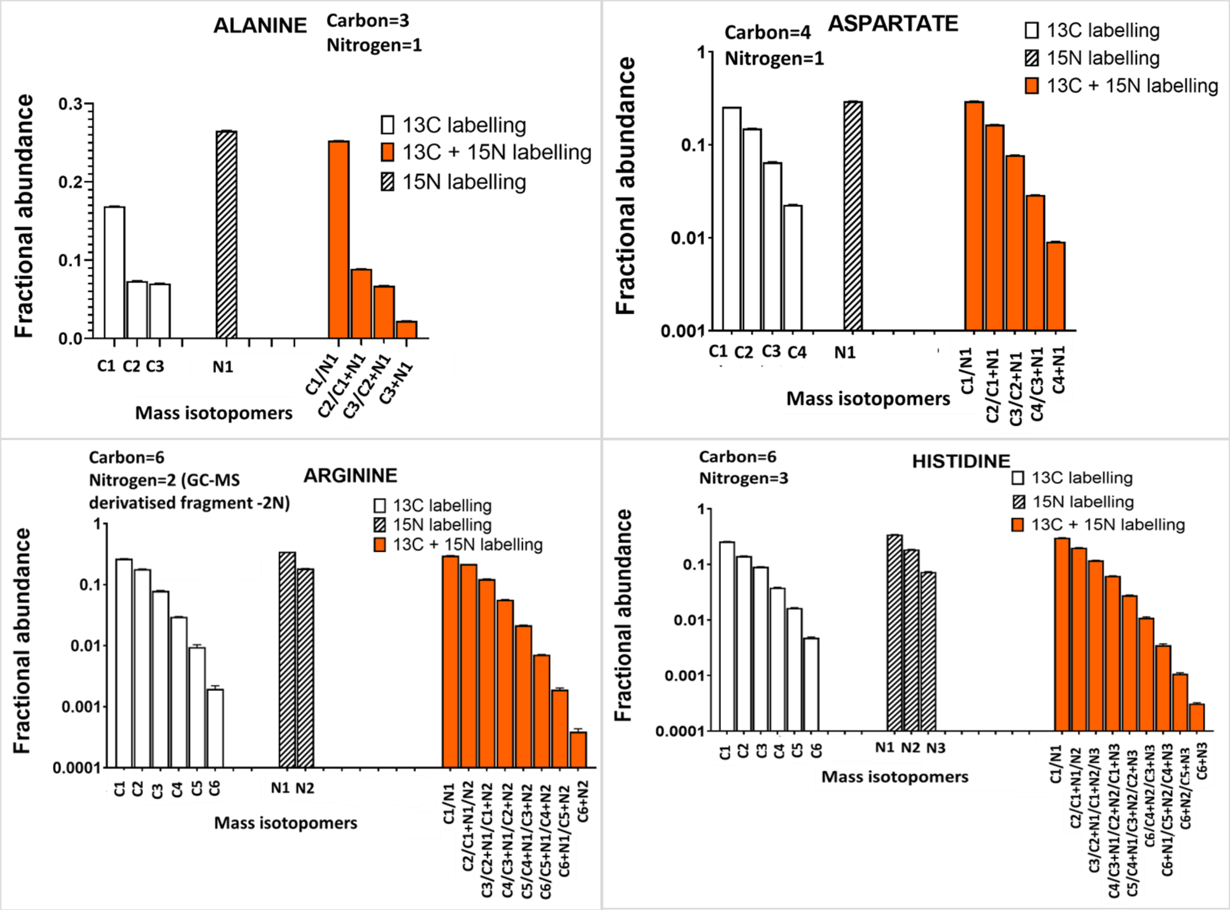

Appendix Figure S8. **Mass isotopomer distributions for amino acids alanine, serine, arginine, and histidine obtained from ^{13}C-glycerol (condition 1), 15N-ammonium chloride (condition 2) and ^{13}C-glycerol + ^{15}N-ammonium chloride (condition 3) labelling**. Datasets show the comparisons between the three labelling conditions. ^{13}C-glycerol (condition 1) labelling experiment provides only Carbon (C) data. ^{15}N-ammonium chloride (condition 2) labelling experiment provides only nitrogen (N) data. ^{13}C-glycerol + ^{15}N-ammonium chloride (condition 3) labelling experiment provides both C and N (C + N) data. C1, C2, C3…C6 are carbon isotopomers; N1, N2, N3 are nitrogen isotopomers and C1+N1…..C6+N1, C6+N2, C6+N3 are carbon + nitrogen isotopomers. Values are mean ± S.D. (n=3).

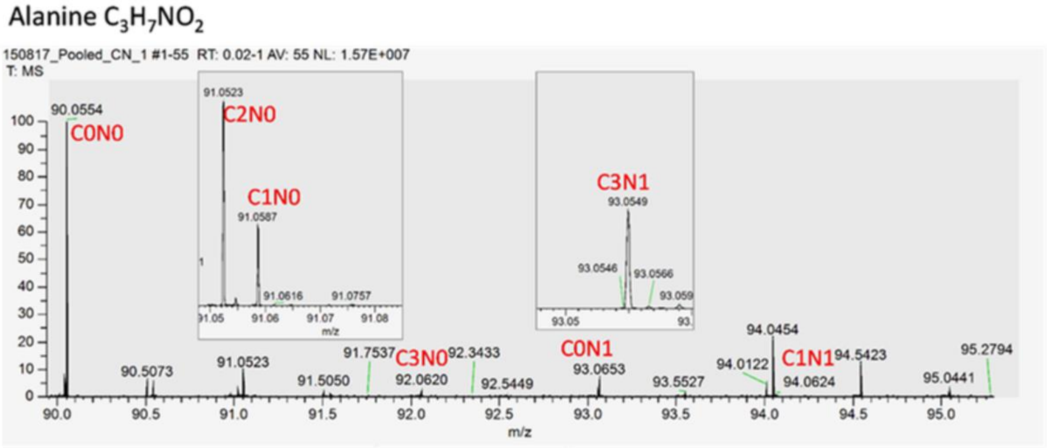

Appendix Figure S9. **$^{13}C^{15}N$ labelling of alanine in *M. bovis* BCG chemostat cultures measured using orbitrap MS**.

Seven mass isotopomers marked in the spectrum shows ^{13}C and ^{15}N incorporation in alanine. Mass isotopomers m/z 90.0554: C0N0 (unlabelled carbon and nitrogen); m/z 91.0587: C1N0 (one ^{13}C and unlabelled nitrogen); m/z 91.0523: C2N0 (two ^{13}C and unlabelled nitrogen); m/z 92.060: C3N0 (three ^{13}C and unlabelled nitrogen); m/z 93.0653: C0N1 (unlabelled carbon and one ^{15}N); m/z 94.0624: C1N1 (one ^{13}C and one ^{15}N); m/z 93.0549: C3N1 (three ^{13}C and one ^{15}N). Values are ± s.d. (n=3-4 measurements each from two independent chemostats).

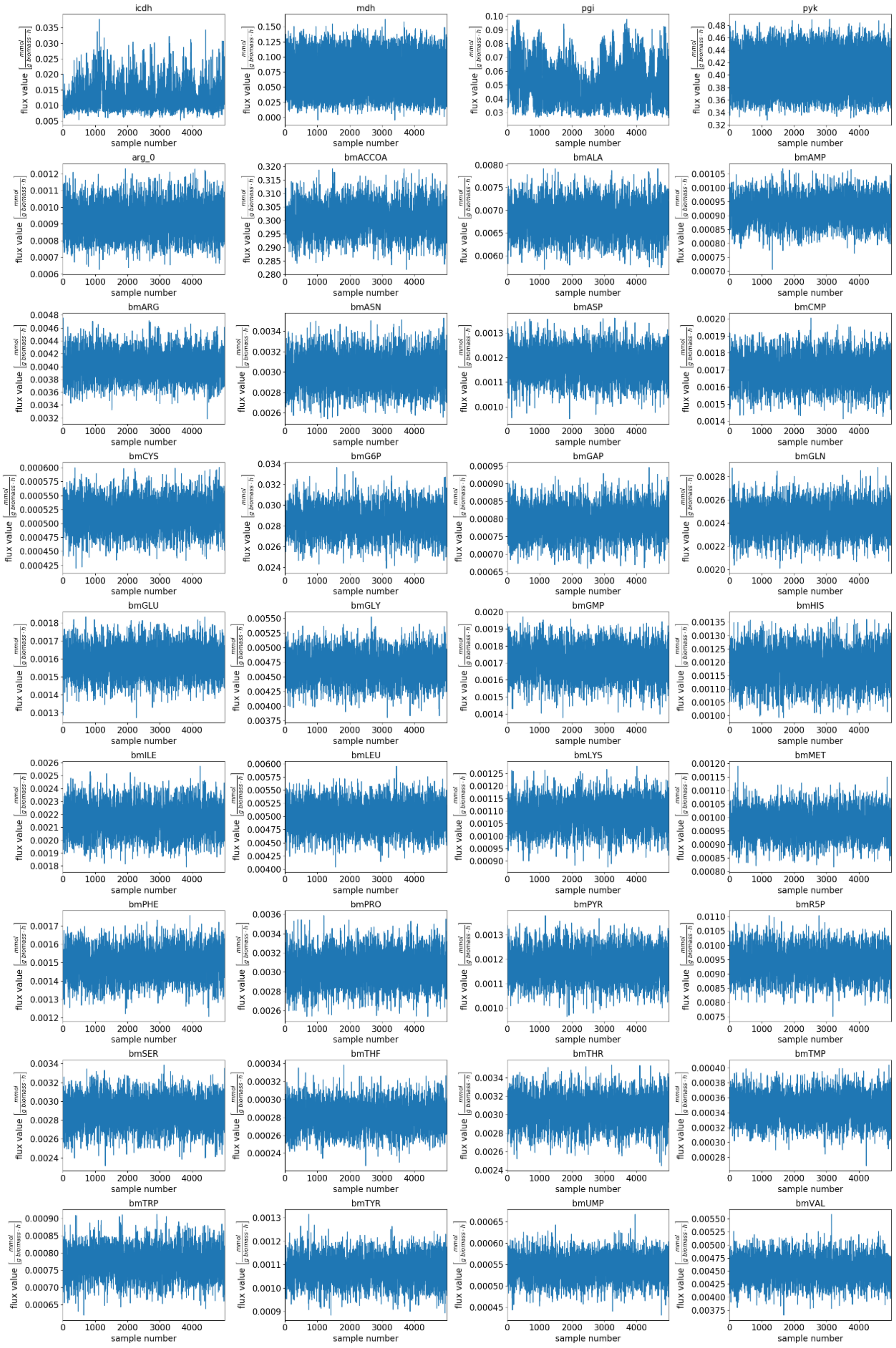

Appendix Figure S10. **Mixing plots of the free net fluxes (one representative replicate)**. The MCMC algorithm is run for $1.5.10^7$ iterations. The first $5{\cdot}10^6$ samples were treated as burn-in and were, therefore, discarded. Of the following 10^7 samples, each 2,000th sample was saved (thinning). This leads to 5,000 samples per MCMC replicate. The trace plots indicate proper mixing of the Markov chain in the flux space.

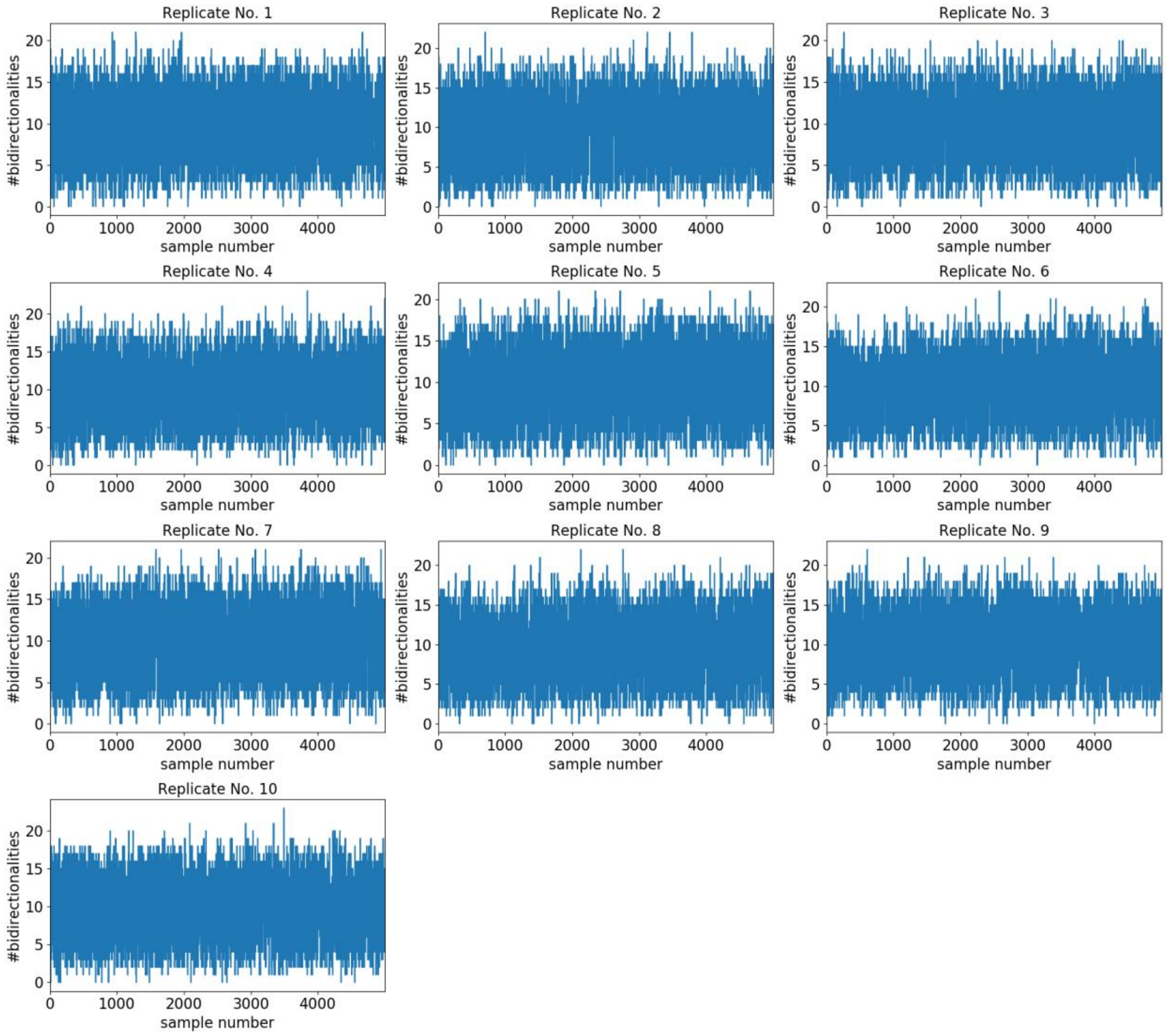

Appendix Figure S11. **Mixing plots in model space (all replicates)**.
To certify that the replicates did not get stuck in different regions of model space, all replicates are shown. Per replicate, the number of bidirectionalities was quantified. Each subplot represents one independent MCMC run. The plots show that the sampler mixes well in the model space and that the mixing is very reproducible for all 10 replicate chains.

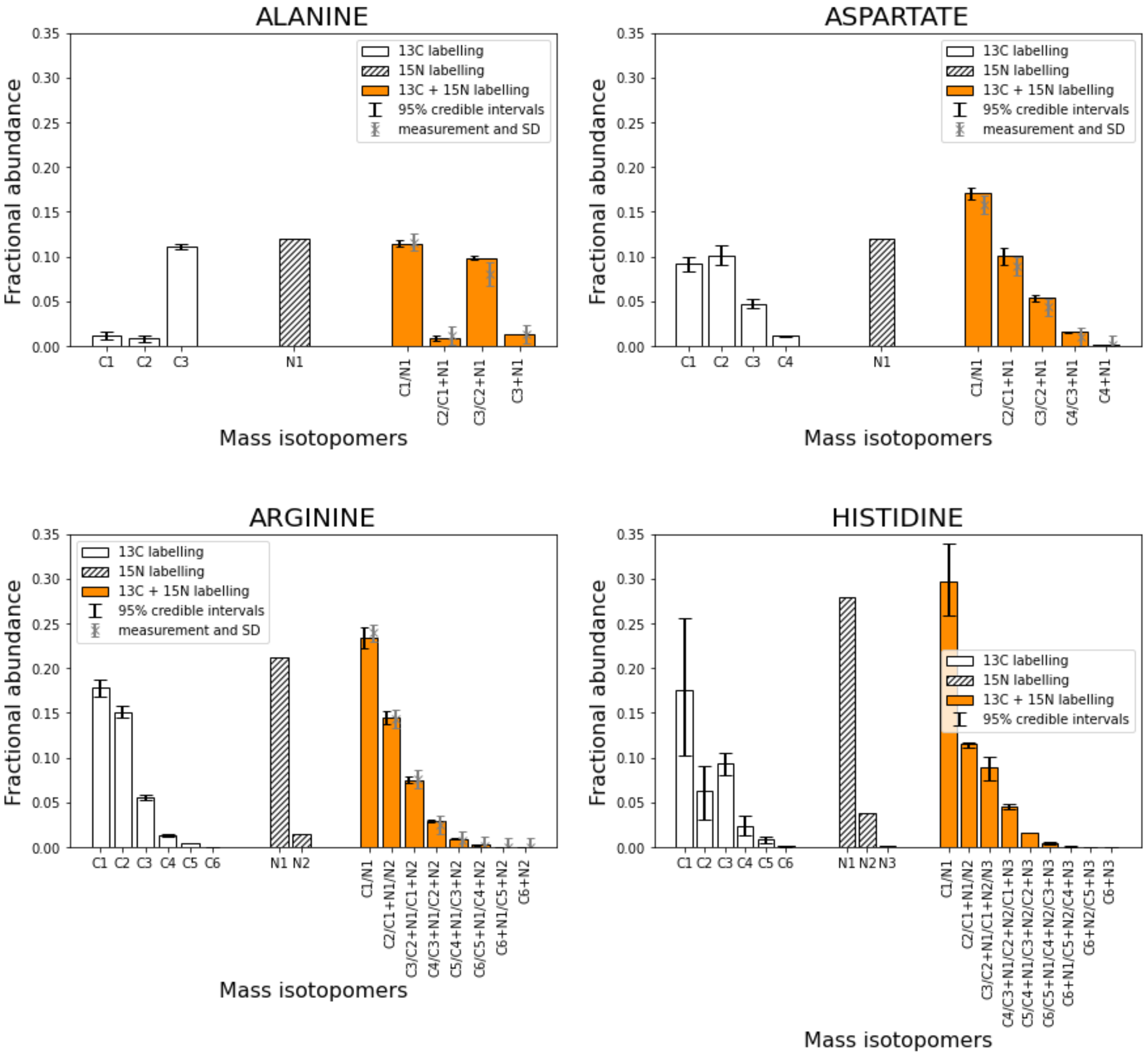

Appendix Figure S12. **Simulated mass isotopomer distributions**.

Mass isotopomers were simulated for amino acids alanine, serine, arginine and histidine obtained by sampling posterior distributions obtained from BMA-based $^{13}C^{15}N$-MFA at metabolic and isotopic stationarity for conditions (a) 12% [$^{13}C_3$] glycerol + 0% [$^{15}N_1$] ammonium chloride, (b) 0% [$^{13}C_3$] glycerol + 12% [$^{15}N_1$] ammonium chloride, and (c) 12% [$^{13}C_3$] glycerol + 12% [$^{15}N_1$] ammonium chloride. The value given represents the expected fractional enrichment, the error bars represent the 95% credible intervals. For the co-labelling strategy, observed data are given in addition.
