# Supplementary material for: One‐shot 13C15N‐metabolic flux analysis for simultaneous quantification of carbon and nitrogen flux
Source: Mol Syst Biol. 2023 Jan 27;19(3):e11099. doi: 10.15252/msb.202211099 (PMC9996240; doi:10.15252/msb.202211099)
Supplement: Supplementary file 10 [file MSB-19-e11099-s005.pdf]

molecular systems biology

One-shot 13C15N-metabolic flux analysis for simultaneous quantification of carbon and nitrogen flux

Khushboo Borah, Martin Beyß, Ye Xu, Jim Barber, Catia Costa, Jane Newcombe, Axel Theorell, Melanie Bailey, Dany Beste, Johnjoe McFadden, and Katharina Nöh
DOI: 10.15252/msb.202211099

Corresponding author(s): Johnjoe McFadden (j.mcfadden@surrey.ac.uk) , Katharina Nöh (k.noeh@fz-juelich.de)

Review Timeline:

Submission Date:	28th Apr 22
Editorial Decision:	31st May 22
Revision Received:	27th Aug 22
Editorial Decision:	7th Oct 22
Revision Received:	14th Nov 22
Editorial Decision:	13th Dec 22
Revision Received:	6th Jan 23
Accepted:	13th Jan 23

Editor: Maria Polychronidou

Transaction Report:

(Note: With the exception of the correction of typographical or spelling errors that could be a source of ambiguity, letters and reports are not edited. Depending on transfer agreements, referee reports obtained elsewhere may or may not be included in this compilation. Referee reports are anonymous unless the Referee chooses to sign their reports.)

1st Editorial Decision **31st May 2022**

Thank you again for submitting your work to Molecular Systems Biology. We have now heard back from the three reviewers who agreed to evaluate your study. As you will see below, the reviewers think that the presented methodology seems potentially interesting. They do however list a series of concerns, which we would ask you to address in a revision.

Without repeating all the comments listed below, some of the most important points are the following:

- Reviewers #1 and #3 point out that additional analyses should be performed to better support the superiority and advantages of the presented approach compared to alternative approaches.

- Reviewer #3 mentions that the new biological insights remain rather limited. They do mention however that there are opportunities to improve this by following up on specific findings (e.g. the finding that at least one of the anaplerotic reactions runs in reverse).

All issues raised by the reviewers need to be satisfactorily addressed. Please contact me in case you would like to discuss in further detail any of the issues raised, I would be happy to schedule a call. As you may already know, our editorial policy allows in principle a single round of major revision, so it is essential to provide responses to the reviewers' comments that are as complete as possible.

On a more editorial level, we would ask you to address the following points:

Reviewer #1:

In this manuscript, the authors developed a Bayesian multi-model flux analysis approach and applied it to quantifying both carbon and nitrogen fluxes in Mtb. Given the low demand on experimental input (e.g., non-separated N and C labeling and labeling data of a small number of metabolites) and the informative flux results, this approach is a potentially valuable tool for the flux community. However, to be really convinced of its value, I'd like to see more information on how the approach is superior to the traditional 13C-MFA approach.

One way to demonstrate the better performance of the multi-model approach is to directly compare it with the traditional single-model approach. Run both of them on the same dataset and compare the flux results. This can be done either for 13C-labeled data or 13C- and 15N- labeled data. The single model can encompass all the degrees of freedom (e.g., reaction reversibility) in the multi-model approach.

It'd also helpful to dissect the multi-model procedure and provide more explanation on why it is necessary to consider multiple models. For example, it'd be useful to show and discuss the distribution of p(Mi/D).

Reviewer #2:

The paper by Borah et al reports on a ne-shot 13C15N-metabolic flux analysis for simultaneous 2 quantification of carbon and nitrogen flux.

The study uses dual 13C and 15N labelling to resolve N and C fluxes in mycobacteria.

The paper is very well written and presented, is interesting and I have very few comments to add. I am not qualified to comment on the Baeysian model used and so have not reviewed this part of the manuscript. I think this manuscript should be published.

Line 215 - would be useful to expand on gene names and add in their function.

Line 240 - why is it unlikely both carry zero flux - can you expand on this?

Line 315 - can you comment on what is known about directionality of transaminases in other organisms and why you chose to model bidirectional reaction steps. How would this differ if not bidirectional?

Discussion - experiments carried out in BCG, can authors comment on how applicable findings are to Mtb in terms of pathways and metabolism.

Methods - what temperature was chemostat maintained at?

Fig 1 - insert with chemical structures looks low resolution and very small.

Reviewer #3:

The study presents a quantitative flux analysis of Mycobacterium metabolism with concomitant 13C and 15N labelling. The focus is on technical aspects: extension of atom mapping models to account for both C and N isotopologues. Fluxes were determined with the Bayesian Model Averaging framework described by Thorsell in 2020. The advantages of the co-labelling approach are highlighted by comparing to the flux estimates published in 2011 by the same labs using exclusively 13C-tracing.

The study is technically solid, but more work is needed to demonstrate the benefits of co-labelling over alternative approaches (more below).
In terms of new biology, the novelty is marginal. The reason is that authors limited themselves to highlighting differences between result sets. However, there is room for discussing biological findings and showing that the relevance of co-labelling goes beyond numerical considerations.

Major points:
- Some net fluxes obtained with BMA and 13C15N are substantially different from those obtained with 13C and best-fit MFA (Figure S3). The expectation would be that the best fit given in Beste 2011 is much closer to the credible range obtained by BMA. Why? Is it because of overfitting? Please elaborate.
- On a similar note: when comparing the 13C analysis (Beste 2011) and with the new 13C15N analysis, it is unclear whether the differences are caused by the use of 15N, BMA, or both. Please clarify. I invite authors to estimate fluxes from the Beste 2011 data with BMA to have a clear comparison.
- A big claim is that 15N allows resolving more fluxes, i.e. biosynthetic fluxes to N-containing compounds such as amino acids and nucleotides (for example, on line 386-388). Nucleotides are even claimed to be quantified for the first time (abstract). This is a misrepresentation. All biosynthetic fluxes that diverge from central metabolism are implicitly estimated by growth rate and biomass requirements. I am sure they were calculated also in Beste 2011, Borah 2020, and many other studies before - even though they were not reported. Please be more precise with the wording. To make any statement about the benefits of 15N, one would need to compare the estimates obtained with either 15N13C, 13C, or plain FBA.
- The use of BMA for flux analysis remains controversial. The argument about overfitting in the larger model is academic. Most importantly, the assumption that p(Mi) is equal for all models Mi is against what we know from biochemistry and thermodynamics. Hence, even though BMA is statistically more rigorous, here it rests on assumptions that seem biologically wrong. Thus, the BMA seems biased by neglecting evident prior knowledge. Is it any better than assuming that all reactions are reversible?
- How does one-shot 15N13C compare against parallel experiments with 15N and 13C? There is a long history of doing parallel labelling experiments, and they have the advantage that 13C and 15N labeling patterns can also be distinguished on low-resolution instruments. Given the increased information, flux estimates should be even better. Please elaborate.
- The discussion of biological findings should be improved. First, the conclusion that at least one of the anaplerotic reactions runs in reverse has significant consequences. I am surprised that the authors are passing on the opportunity. Is it cycling thermodynamically feasible? Would a reversed anaplerotic reaction be able to support gluconeogenesis? This seems a novel finding across microorganisms.
- Second, the conclusion that oleic acid affects lower glycolysis is merely speculative. What would be the mechanism? Beta-oxidation to AcCoA? Allosteric? Please prove it or tone down the statement.
- Third, the new analysis reveals that the estimates provided by Beste 2011 were not correct. As this also the case for TCA cycle fluxes (sdh, fum, kor) and the pyruvate node, it would be sensible to elaborate whether the original conclusion about the relevance of the isocitrate lyase for pyruvate dissimilation is still valid.

Minor points:
- I could not follow the argument on high resolution vs low resolution MS. The authors used low resolution instruments, which seems to be enough in this case. However, they state "that FT instruments operate at a tradeoff between resolving power and acquisition accuracy" (lines 146-147). I disagree: there is a tradeoff between resolution and speed, but not on the accuracy of isotopic ratios. Please clarify or provide compelling references.
- The concept of "carbon-nitrogen flux" is confusing. The term is used to indicate something that differs from carbon and nitrogen fluxes (for example, in the abstract), even though it is just a combination of the two. Please avoid and rephrase.
- Line 379: Given the uncertainty shown in Figure 4 and S4, it doesn't seem correct to claim that "13C15N-MFA provides an effective tool to constrain the anaplerotic net fluxes".
- The method section on "Mass spectrometry analysis of amino acids" contains information on collecting high-resolution data with an Orbitrap, but nothing is mentioned in the main text. It also references a figure S7, which is wrongly labelled. Remove everything or embed it properly.
- A gltBD mutant is mentioned in the methods, but it wasn't used in the study.
- The biomass requirements should be reported somewhere.

Dear Maria,

Thank you for providing us with the peer review of our manuscript. We value the editorial and reviewers' comments which have immensely helped us to improve our manuscript. We have addressed each of the reviewers and the editorial comments in details. We have done new analyses, generated new figures and revised the text to improve our manuscript. Please find below the detailed response to the three reviewers' comments. We hope that you will be able to consider our revisions and publish our manuscript with Molecular Systems Biology.

Response to Reviewer comments: MSB-2022-11099, One-shot $^{13}C^{15}N$-metabolic flux analysis for simultaneous quantification of carbon and nitrogen flux

Reviewer #1:

In this manuscript, the authors developed a Bayesian multi-model flux analysis approach and applied it to quantifying both carbon and nitrogen fluxes in Mtb. Given the low demand on experimental input (e.g., non-separated N and C labeling and labeling data of a small number of metabolites) and the informative flux results, this approach is a potentially valuable tool for the flux community. However, to be really convinced of its value, I'd like to see more information on how the approach is superior to the traditional 13C-MFA approach.

Response: We thank the reviewer for the excellent summary that precisely captures the essence of our paper. Before answering the questions, please allow us to make a general comment on why we decided to not apply traditional ^{13}C-MFA in this work.

The goal of ^{13}C-MFA – we use the term, as it is common in the community, independent of the tracer, such as ^{15}N, or tracer combination, such as $^{13}C^{15}N$ – is to quantify as many metabolic fluxes as possible in a living organism. Quantification here means to estimate the fluxes including their uncertainty from a given set of noisy data using metabolic modelling.

Our multi-model approach tackles two limitations of the traditional single-model ^{13}C-MFA approach:

(1) In all but trivial cases, some model parameters (fluxes) remain indeterminable from the data. To deal with these identifiability issues, modellers employ (often local) sensitivity and identifiability analyses followed by implementing model reductions, such as parameter fixations. Model reductions, however, impose potential biases to the flux inferences. Unfortunately, in the current ^{13}C MFA practice neither the reduction workflows are documented, nor the flux estimates are discussed in the light of the introduced assumptions.

(2) Single-model ^{13}C-MFA assumes the correct model formulation used for flux inference to be known. However, in all but well-studied cases, there is some model uncertainty. When the goal is to learn about the model structure (and the underlying biological process), traditional single-model approaches

cannot be used due to the combinatorically many model candidates ensuing from the pathway structure and flux non-identifiability.

The core advantage of our multi-model approach is that it enables us to deal with both, model uncertainty *and* parameter non-identifiability. This alleviates the need to discuss the results in the light of simplifying assumptions and to defend model reductions.

In our work, the biological question at hand, i.e., studying carbon and nitrogen metabolism in Mtb, enforced us to extend the scope of the model towards nitrogen metabolism. As this extension induced uncertainty about the model formulation, we decided to apply a multi-model approach to adequately account for and deal with this uncertainty. We revised the introduction to improve clarity, why we opted for the application of BMA-based $^{13}C^{15}N$ to quantify CN fluxes in *M. bovis* BCG (L124-135).

Changes made in the revised abstract:

Lines 29-40. Here we performed $^{13}C^{15}N$ dual isotopic labelling of mycobacterial steady state cultures and quantified intracellular carbon and nitrogen (CN) fluxes and inferred their reaction bidirectionalities. Studying carbon along with nitrogen metabolism was achieved by model scope extension and refinement, implemented in a multi-atom transition model. Within the statistical framework of Bayesian model averaging, we were able to resolve CN fluxes quantitatively under model uncertainty, despite non-separable $^{13}C^{15}N$ labelling data. We quantified CN fluxes for amino acid and nucleotide biosynthesis, confirmed glutamate as the central node in mycobacteria and improved the flux resolution of the anaplerotic node, revealing novel insights into possible anaplerotic operation modes. Our study describes a powerful, low demand platform to measure carbon and nitrogen metabolism in any biological system with statistical rigor.

Reviewer comment 1.1: One way to demonstrate the better performance of the multi-model approach is to directly compare it with the traditional single-model approach. Run both of them on the same dataset and compare the flux results. This can be done either for 13C-labeled data or 13C- and 15N- labeled data. The single model can encompass all the degrees of freedom (e.g., reaction reversibility) in the multi-model approach.

Response 1.1: We appreciate this comment and apologize if our manuscript gives the impression that we solve an old problem with a new method. However, as we explained above, the switch in methodology was motivated by the need to deal with model uncertainty emerging from the biological question.

In a previous work (10.1093/bioinformatics/btz500), we described the computational machinery that allowed us to address ^{13}C-MFA problems with BMA in a computationally efficient manner. In that work, we compared the traditional single-model ^{13}C-MFA approach with the multi-model ^{13}C-MFA approach and highlighted the strength of the latter, in the fashion suggested by the reviewer. This comparison was performed using a known (simulated) ground truth data set for *E. coli.*

Technically, a comparison of multi- and single-model ^{13}C-MFA approaches for the same data is not straightforward. The outcome of the single-model approach depends on a fixed model formulation. In particular, when we use an overly complex model, the inferences may be affected by overfitting (to many degrees of freedom compared to the data at hand); and when we reduce the complexity, we introduce possibly biasing assumptions during the process. We argue that this potential pitfall of the single-model approach can only be tested under defined synthetic conditions, as done in 10.1093/bioinformatics/btz500.

The multi-model approach avoids both, the need for laborious model reduction and the risk of overfitting. Indeed, in Bayesian statistics the concept of non-identifiability is meaningless (10.1002/bit.26379). Instead, our Bayesian multi-model approach pinpoints, which modelling assumptions are supported by the data. For instance, the $^{13}C^{15}N$ data studied in our work qualifies 7 reactions unidirectional, and 1 reaction bidirectional. Thus, a reduction by 7 (labelling exchange) flux parameters (setting them to zero) is supported by the data and will not introduce a bias.

Summarizing, we use the multi-model approach since it is the state-of-the-art statistical method when working with underdetermined inference problems. The alternate option, using a single model and employ (local) identifiability analysis to reduce the model, implies that several assumptions have to be made that cannot be tested experimentally. Within the multi-model approach, such assumptions are not needed, which highlights the conceptual superiority of our approach. We extended the discussion by listing the distinguishing features of our approach (see also Response 1.2 below).

Reviewer comment 1.2: It'd also helpful to dissect the multi-model procedure and provide more explanation on why it is necessary to consider multiple models. For example, it'd be useful to show and discuss the distribution of p(Mi/D).

Response 1.2: We thank the reviewer for this supportive comment.

We hope that Response 1.1 clarified the reasoning why the multi-model approach was necessary. These arguments are also brought forward in the main text (Lines 90-96; and Lines 115-132).

The opportunity to derive model probabilities is a powerful feature of multi-model approaches. In our case, we have to deal with combinatorially many models Mi, where each model has a unique combination of uni/bidirectionalities. With 35 potentially bidirectional reactions, we have to deal with 2^{35} (more than 3e10) models. However, discussing $p(M_i|D)$ for indiviudal models M_i is clearly not practical. Instead, we discuss the probabilities of individual reactions to be bidirectional (Figure S6). The comments encouraged us to make the main motivations and distinguishing features more prominently in the text.

Changes made in the revised manuscript:

Line 395-407:

- The Bayesian framework allows approximating the full posterior probability distributions of the net fluxes from which accurate nonlinear credible intervals (1D) and credibility regions (2D) are derived.
- The multi-model approach captures flux parameter and model formulation uncertainty, thereby providing consolidated flux uncertainty quantification.
- BMA-based $^{13}C^{15}N$-MFA reliably evaluates low-demand co-labelling data, without the need of settling for potentially biasing model reductions.

These key advantages of BMA-based $^{13}C^{15}N$-MFA, together with the extension of scope and refinement in terms of the C and N metabolic network, enable us to estimate C and N fluxes in one consolidated approach and provide reliable estimates for the uncertainties in the parameters.

Further revised changes are included in Lines 176-178, 355-358, 365-367.

Reviewer #2:

The paper by Borah et al reports on a one-shot 13C15N-metabolic flux analysis for simultaneous 2 quantification of carbon and nitrogen flux.

The study uses dual 13C and 15N labelling to resolve N and C fluxes in mycobacteria.

The paper is very well written and presented, is interesting and I have very few comments to add. I am not qualified to comment on the Baeysian model used and so have not reviewed this part of the manuscript. I think this manuscript should be published.

Reviewer 2 comment: Line 215 - would be useful to expand on gene names and add in their function.

Response 2.1: We thank this reviewer for the very positive comments, and we have made the following changes in the revised manuscript (see also Response 3.8).

Changes made in the revised manuscript:

Line 218-223.: Rv2454c-Rv2455c encoding kor and Rv0951-Rv0952 encoding scs in Mtb comprise the decarboxylating steps in the TCA cycle, which are bypassed using glyoxylate shunt, instantiating the GAS pathway (Beste et al, 2011). The GAS pathway utilizes the glyoxylate shunt and anaplerotic reaction for oxidation of pyruvate. The operation of this pathway has been measured using our current BMA analysis, consistent with the results derived previously using ^{13}C-MFA (Beste et al, 2011).

Reviewer 2 comment: Line 240 - why is it unlikely both carry zero flux - can you expand on this?

Response 2.2: Our Bayesian analysis equips each flux with a probability in view of the data (Figure EV2). Visualizing these numbers for pairs of anaplerotic fluxes gives characteristic 2D "donut" patterns, shown in Figure 5. Here, taking *pck/mez* as an example, the constellation that these two fluxes are simultaneously zero, is low ((0,0) is located within the "hole" in the donut, well outside of 95% credible region indicated by the dashed line). The same holds for the flux pairs *pck/pca and mez/pca*. This explains why it is very unlikely that both fluxes of these three pairs carry zero flux. Notice that this information cannot be taken from the 1D credibility intervals in Figure 4.

This insight, which was solely inferred from the investigated data set and not implemented in the metabolic model, is in line with biological knowledge for Mtb. For example that anaplerotic reactions catalyses gluconeogenic utilization of carbons to replenish the TCA cycle (e.g. doi: 10.1074/jbc.RA118.001839).

With the revision, we improved the readability of Figure 5 by including 0 grid lines and 95% credible regions; extended explanations are provided to make the plots

more accessible. To give a better intuition for the donut and its interpretation, we configured a 3D plot for *pck/pca/mez* for the reviewer. Please see 3DCrl attached file which is for the reviewer's assessment purposes.

Changes made in the revised manuscript:

Lines 250-290: Despite limited information in the co-labelling data for identification of the directionalities of the anaplerotic reactions, we were able to refine the resolution of the flux map and limit the absolute flux values to a range of ± 0.06 mmol g biomass-1 h-1 (approx. 10% of glycerol uptake). However, the two-dimensional (2D) marginal posterior probability distributions shown in Fig 5, and in the extended version Fig EV4, show that the information contained in the 13C15N data set effectively narrows down the joint space of possible values considerably further to concise, ring-like regions within the flux space. With these inferences, many flux constellations are ruled out. For instance, given the data set at hand, it is very unlikely that the reaction pairs pca and pck, pca and mez, or pck and mez, carry zero fluxes. This insight, which was not implemented in the flux model a priori, is indeed experimentally supported: the mentioned anaplerotic reactions catalyse gluconeogenic utilization of carbons to replenish the TCA cycle and operation of these fluxes has been demonstrated to be important for the survival of Mtb (Basu et al, 2018). In the previous analysis in Beste et al. (Beste et al, 2011), we were unable to derive such precise information due to model simplifications enforced by flux non-identifiabilities (Beste et al, 2011).

From the 2D marginal posterior flux probability distributions in Fig 5 we further see that the flux pairs pck/mez and pck/pca are largely positively correlated, meaning that a larger value of one flux implies a larger value of the other. In contrast, pca and mez are largely negatively correlated. Thus, at least one of the three anaplerotic reactions is operating in gluconeogenetic direction. In a larger context, Fig S4 shows that mdh and pyk are highly correlated with mez and pck, respectively.

Beyond such qualitative assessment of the flux inferences, Fig 5 shows the most likely flux operation modes, i.e. flux constellations that are located in the highest probability region. Here, we discuss the most likely flux mode, indicated by a circle (o) in Fig 5 A, B, and C. In this mode, mez is 0.05 mmol g biomass-1 h-1) and pca is net positive, while the pck net flux is zero. Together, this operation mode, shown in Fig 5D, poses a futile cycle: starting from phosphoenolpyruvic acid, carbon flows via pyk and mez to form malate, which is then transformed gluconeogenetically via mdh and pck back to phosphoenolpyruvic acid. Alternatively, the less likely, flux operation modes for the anaplerotic node are detailed in Fig EV4.

In conclusion, the C flux profile for BCG growing at a faster growth rate (0.03 h-1) inferred here by BMA-based 13C15N-MFA represents an independent replication of the previous 13C-MFA-derived C fluxes. The C flux maps derived from the two

labelling approaches and the two MFA platforms are comparable, while our current Bayesian approach imposes fewer modelling assumptions, provides more reliable flux uncertainties, and delivers an increased flux resolution, in particular for the previously non-inferable anaplerotic node in BCG. The results from our Bayesian analysis provide explanations of the analysed data set, which narrow down the range of likely fluxes (Fig 4) to concise possible functional anaplerotic flux modes (Fig 5).

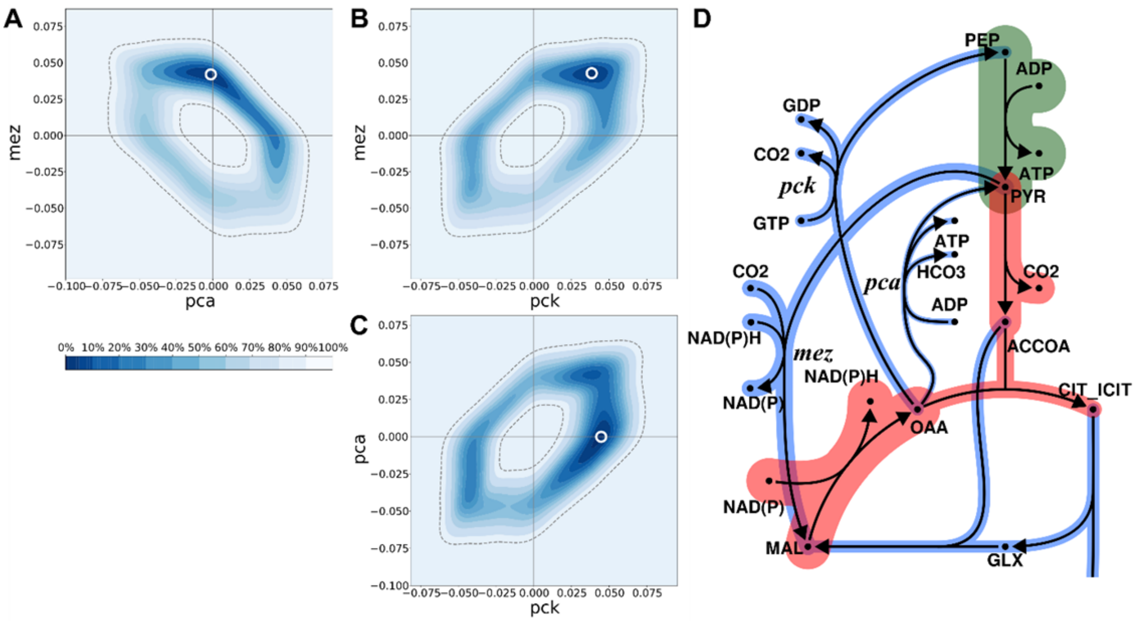

Figure 5. Marginal posterior probabilities and operation modes of the anaplerotic node. (**A,B,C**): Joint marginal flux posterior distributions for the three anaplerotic reactions pyruvate carboxylase (*pca*), PEP carboxykinase (*pck*), and malic enzyme (*mez*). Darker (lighter) colors indicate regions of higher (lower) flux probability given the labelling data. The dashed lines indicate the 95% 2D credibility region, horizontal/vertical lines indicate zero fluxes. For *mez* vs *pca*, *mez* vs *pck*, and *pca* vs *pck* fluxes ring-like shapes emerge, revealing complex correlations between the fluxes. Apparently, the reaction pairs are unlikely to both carry zero flux. The symbol “o” marks the most likely flux values given the $^{13}C^{15}N$ data set. (**D**): The metabolic network of the anaplerotic node, and the most likely fluxes labelled by “o” in A, B, C. Starting from phosphoenolpyruvic acid, carbon flows via *pyk* and *mez* to malate, which is then transformed via *mdh* and *pck* back to phosphoenolpyruvic acid. See supplementary Fig. S4 for an extended version of this figure, and interpretation of further possible flux constellations.

Further revised changes are included lines 438-441.

Reviewer 2 comment: Line 315 - can you comment on what is known about directionality of transaminases in other organisms and why you chose to model bidirectional reaction steps. How would this differ if not bidirectional?

Response 2.3: We have to carefully distinguish between reaction directionalities (predominant direction of net flux) and bidirectionalities (simultaneous operation of forward and backward flux).

In our experimental setup, unsurprisingly, the directionalities of the transaminases are directed from central carbon metabolism towards the amino acids, which are essential for growth and biomass production.

Bidirectionatlities, on the other hand, are required to model fractional labelling enrichments: Whether reactions carry only the forward (unidirectional) or forward and backward (bidirectional) flux determines much of the labelling patterns. If reactions are modelled unidirectional that are actually bidirectional, this will introduce a bias, which may lead to wrong flux inferences. This is what we explain in Lines 335-346. Generally, all reactions operating, or are suspected to operate, close to thermodynamic equilibrium are therefore considered bidirectional in this study. For a more in depth, explanation how we treat bidirectionalities, please see our Response 3.5.

Transaminases are highly promiscuous enzymes that have been shown to operate flexibly in living organisms (Grotkjær et al, 2004, Wahrheit et al, 2014). For example, a recent paper explored the amination network in *E. coli* lacking glutamate synthesis (10.1101/2022.01.25.477661v1.full). The auxotroph was rescued by multiple transaminases including aspartate transaminase (*AspC*) as a major one. It is likely that transaminases operate near thermodynamic equilibrium, in order to serve such rescuing functionality if needed by the cells.

Mycobacterial transaminases in particular are not well studied. We therefore set transaminase reactions in the model to bidirectional to capture this flexibility of the amination network and to remove any bias that would arise from setting a transamination reaction as unidirectional. This model uncertainty leads to a high number of additional flux parameters, and motivated the decision to use the multi-model approach, rather than using traditional single-model ^{13}C-MFA technique.

Changes made in the revised manuscript:

Lines 356-359: Due to the lack of evidence about the reversibility of mycobacterial transaminases, we set the transaminase reactions in the model as potentially bidirectional to capture the flexibility of the amination network and to avoid the risk of any bias that would arise from setting a transamination reaction erroneously unidirectional.

Reviewer 2 comment: Discussion - experiments carried out in BCG, can authors comment on how applicable findings are to Mtb in terms of pathways and metabolism.

Response 2.4: We have made the following changes in the revised manuscript:

We have explained the use of BCG as the showcase for our Bayesian analysis as it is the relevant model for Tuberculosis in Lines 94-96. BCG and Mtb share >99% sequence similarity.

We have included an explanation in our discussion to highlight the relevance of our BMA-based analysis in BCG and application to Mtb.

Changes made in the revised manuscript:

Lines 411-414.: Our previous work using ^{13}C-MFA demonstrated conserved carbon flux distributions between BCG and Mtb during on glycerol demonstrating metabolic homogeneity between the two mycobacterial systems (*Beste et al, 2011*). Therefore, our BMA-based C and N flux distributions in BCG are relevant to studying the pathogenic Mtb.

Reviewer 2 comment: Methods - what temperature was chemostat maintained at?

Response 2.5: The chemostat or the bioreactor was maintained at 37°C. We have detailed the growth parameters in Table EV1.

Reviewer 2 comment: Fig 1 - insert with chemical structures looks low resolution and very small.

Response 2.6: We have provided an enlarged Figure 1 inset as Figure EV10 in supporting information.

Reviewer #3:

The study presents a quantitative flux analysis of Mycobacterium metabolism with concomitant 13C and 15N labelling. The focus is on technical aspects: extension of atom mapping models to account for both C and N isotopologues. Fluxes were determined with the Bayesian Model Averaging framework described by Thorsell in 2020. The advantages of the co-labelling approach are highlighted by comparing to the flux estimates published in 2011 by the same labs using exclusively 13C-tracing. The study is technically solid, but more work is needed to demonstrate the benefits of co-labelling over alternative approaches (more below).

In terms of new biology, the novelty is marginal. The reason is that authors limited themselves to highlighting differences between result sets. However, there is room for discussing biological findings and showing that the relevance of co-labelling goes beyond numerical considerations.

Response: We appreciate the comment and agree that the experimental scenario was already studied before, though with less resolution and scope. Although we do not deliberately intend to shape our work towards "technical" aspects, the need to elaborate the sheer number of differences compared to the traditional analysis may give the impression that our work has primarily a methodological character. As also detail in Responses 1.1. and 2.3, these methodological differences emerged from the need to model the data set and the biological question at hand.

However, we agree with this reviewer that beyond comparing results with previous ones in terms of differences and commonalities, discussing new biological findings is important. From our perspective the major ones are the following (see also Response 2.2):

- The scope of the analysis was extended from carbon to nitrogen metabolism, including nucleotide metabolism and an improved/refined biomass formulation. Therewith, we provide the first consistent flux map of central carbon and nitrogen metabolism, where the uncertainty in the model formulation used for flux inference is reliably represented.
- By deriving 2D posterior distributions, complex flux correlations are found, providing novel insights in, for example, the anaplerotic node. Some of the results are confirmed qualitatively by outcomes of former knockout studies, others can be used to guide experimentation in the future.

We agree with the reviewer that we should present the biological findings more prominently. With the revision, we made several additions, as explained in detail below.

Reviewer 3 comment: Major points:

- Some net fluxes obtained with BMA and 13C15N are substantially different from those obtained with 13C and best-fit MFA (Figure S3). The expectation would be that the best fit given in Beste 2011 is much closer to the credible range obtained by BMA. Why? Is it because of overfitting? Please elaborate.

Response 3.1: The reviewer brings up an important point.

With the revision, we have updated Figure EV3 to better capture similarities and dissimilarities in the flux values estimated of the two studies. We included the stoichiometrically feasible flux ranges for those fluxes that had to be fixed in the evaluations published previously (Beste et al, 2011) due to non-identifiability. Also, instead of absolute values, we now present relative flux values (to glycerol uptake) as it is community typical.

While fluxes of upper glycolysis, PPP, anaplerosis and right branch of the TCA are very similar as indicated by the largely overlapping flux ranges, indeed we found discrepancies in the lower/left part of the TCA (*sdh, kor, fum, sds*) and in the lower glycolysis (*pdh*). The latter is explained by differences in the media used for the cultivations, precisely by the absence of oleic acid from the medium in our work, compared to Beste (2011). The discrepancies in the TCA are considerably smaller, in relative, but also absolute levels. Such small deviations can be explained by (1) differences in the data, (2) the biasing domino effect of flux fixations previously introduced to tackle overfitting, or (3) discrepancies in the model formulation (biomass equation). Importantly, the overall finding – the GAS pathway – is prominently emerging from both analyses.

Besides reporting previously reported flux values, we added ranges to those fluxes that had to be fixed, representing stoichiometrically feasible upper and lower bounds. These ranges are now shown in a lighter shade in the revised Figure S3. Furthermore, instead of absolute fluxes (mmol g biomass^{-1} h^{-1}) relative fluxes (to glycerol uptake) are displayed, easing the comparison. We extended the caption accordingly to clarify the interpretation of this comparison.

Changes made in the revised manuscript:

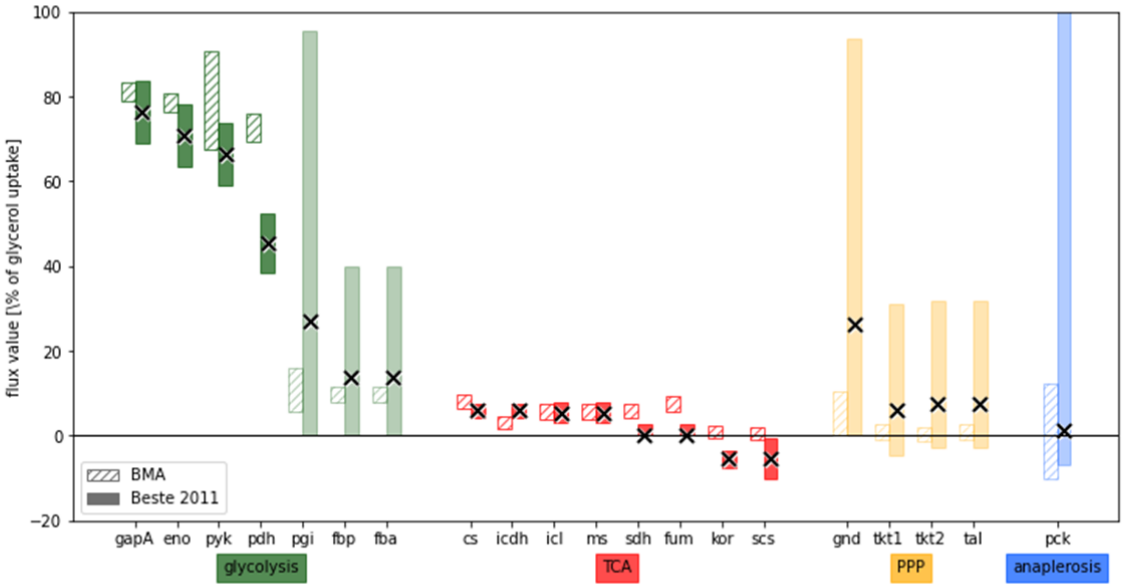

Figure S3. Comparison of net carbon fluxes derived with $^{13}C^{15}N$-MFA (BMA, this work) and ^{13}C-MFA (Beste *et al.*, 2011). 95% credible intervals (CrI) derived using $^{13}C^{15}N$-MFA of central carbon fluxes are shown in shaded bars. Best-fit carbon fluxes derived using ^{13}C-MFA (indicated by the symbol "x") and their associated 95% confidence intervals (CoI, Fisherian standard deviations) are shown in solid bars. Notice that in the study of Beste et al. (2011) several fluxes were either fixed to their best-fit value prior statistical analysis, therewith lacking CoIs, or were lumped, making a direct comparison impossible. For the previously fixed fluxes the stochiometric bounds are shown in lighter shade. Colors are chosen according to the pathway colorings in Figures 1, 3, and 4 in the main text. Differences in profiles occur in fluxes of lower glycolysis (*gapA*, *eno*, *pyk*, *pdh*). Differences in fluxes of *pdh* are explained by the uptake of oleic acid in Beste *et al*. (2011).

Reviewer 3 comment: On a similar note: when comparing the 13C analysis (Beste 2011) and with the new 13C15N analysis, it is unclear whether the differences are caused by the use of 15N, BMA, or both. Please clarify. I invite authors to estimate fluxes from the Beste 2011 data with BMA to have a clear comparison.

Response 3.2: As we understand the question, the reviewer asks for an explanation of the discrepancies in the fluxes determined in the proposed manuscript compared to the best-fit fluxes as determined by Beste et al 2011.

Accepting that the discrepancies in the lower glycolysis are likely originating from a differing carbon source in the medium (oleic acid, see also Response 3.7 below), possible explanations to the remaining minor differences in the TCA are given in Response 3.1 already (see also revised Fig. EV3). Considering the different models/biomass formulations, data sets and analysis methods, small (in absolute terms) discrepancies are rather unsurprising. Here, it should be kept in mind that in Fig. S3 two statistical frameworks resting on different inference interpretations are compared (Frequentist best-fit solutions vs. Bayesian credible intervals), using different modelling assumptions (simplifications), and, most importantly, different underlying uncertainties are considered (data uncertainty vs. data and model uncertainty), explaining why we discuss the outcomes of the two studies in a semi-quantitative fashion.

Concerning the concrete suggestion to repeat the analysis of Beste et al. (2011) with BMA: as we also explain in Response 1.1, such a repetition – with an all-in model – cannot answer the question of the reviewer, about the origin of differences. It rather answers the retrospect question whether the previously made model simplifications/reductions are indeed supported by the data. We agree that such a comparison can provide valuable insights into the previous analysis; however, we consider this question to be outside the scope of this work.

Reviewer 3 comment: A big claim is that 15N allows resolving more fluxes, i.e. biosynthetic fluxes to N-containing compounds such as amino acids and nucleotides (for example, on line 386-388). Nucleotides are even claimed to be quantified for the first time (abstract). This is a misrepresentation. All biosynthetic fluxes that diverge from central metabolism are implicitly estimated by growth rate and biomass

requirements. I am sure they were calculated also in Beste 2011, Borah 2020, and many other studies before - even though they were not reported. Please be more precise with the wording. To make any statement about the benefits of 15N, one would need to compare the estimates obtained with either 15N13C, 13C, or plain FBA.

Response 3.3: We appreciate the reviewers concern and have modified text in the abstract and discussion (Lines 35, 422, 412, 425). That the biosynthetic fluxes are mainly defined by the biomass formulation has been stated in main text of the original submission (Lines 302-304).

We would like to clarify that our CN model is the first to map dual atomic transitions to the nucleotide biosynthesis in mycobacteria. Our previous work involving ^{13}C tracers used network models, which were limited to central carbon metabolism, amino acid synthesis and biomass proportions involving only C contributions. In this work, we have calculated a considerably refined biomass equation, including additional nitrogen contributions to the precursors for amino acid, lipids, protein and nucleotides, which was not done in our previous work. Please see the biomass reaction including C and N atom contributions to the precursors in Table EV2 (see also Response 3.1).

Reviewer 3 comment: The use of BMA for flux analysis remains controversial. The argument about overfitting in the larger model is academic. Most importantly, the assumption that p(Mi) is equal for all models Mi is against what we know from biochemistry and thermodynamics. Hence, even though BMA is statistically more rigorous, here it rests on assumptions that seem biologically wrong. Thus, the BMA seems biased by neglecting evident prior knowledge. Is it any better than assuming that all reactions are reversible?

Response 3.4: We understand the critical comment about overfitting. As it is often the case and we also mention in the manuscript, our motivation is in the avoidance of risk rather than in the fact that overfitting has to be tackled. Despite, we have good reason to address this risk, which is well known in the ^{13}C-MFA field (10.1371/journal.pcbi.1009999, 10.1002/9783527823468.ch3).

Concerning the assumptions: In classical ^{13}C-MFA, the assumptions of reaction uni/bi-directionality are made based on prior knowledge, usually available from thermodynamic knowledge. If a reaction is operating far from (close to) thermodynamic equilibrium under the tested conditions, it is typically set unidirectional (bidirectional). If operating unidirectionally, the labelling exchange flux is set to zero, whereas bidirectionally is enforced by an exchange flux > 0, therewith setting the prior bidirectionality probability to 100%. Int he third case, where it is unclear whether a reaction is actually operating uni- or bidirectionally, the standard way is to opt for bidirectionality to be on the safe side (see also Response 2.3). Importantly, the BMA based approach relies on the exact same grounds.

However, BMA introduces a subtle difference for the third case: When we model a reaction with unknown bidirectionality, the concept of a probability for reaction

bidirectionality is introduced. For this, we set a 50% probability that the reaction is unidirectional and a 50% probability that it is bidirectional. Clearly, if our prior knowledge suggests a different probability ratio (e.g. 90% vs 10%), this can be easily integrated in the analysis.

We fully agree with the reviewer that all available prior knowledge should be exploited in the analysis. In our view, a 50:50 chance is the most reasonable setting in the described case. Therefore, we argue that our BMA modelling approach rests on available prior knowledge and makes biologically reasonable assumptions. We extended the text to clarified this aspect.

Changes made in the revised manuscript:

Lines 579-587: Reactions were classified to be unidirectional (labelling exchange flux = 0), bidirectional (labelling exchange flux > 0) or unknown, i.e. potentially bidirectional (labelling exchange flux ≥ 0). Here, all reactions considered potentially bidirectional, unless evidence was available that the reactions operate close or far from thermodynamic equilibrium under in vivo conditions (supplementary Table S2). In particular, transaminases are modelled potentially bidirectional. Each potentially bidirectional reaction is given a 50:50 probability to be unidirectional or bidirectional, giving rise to combinatorially many structurally different model variants. The probabilistic view enables inference of the probability for a reaction being uni- or bidirectional from the given data.

Reviewer 3 comment: How does one-shot 15N13C compare against parallel experiments with 15N and 13C? There is a long history of doing parallel labelling experiments, and they have the advantage that 13C and 15N labeling patterns can also be distinguished on low-resolution instruments. Given the increased information, flux estimates should be even better. Please elaborate.

Response 3.5: We appreciate the reviewer's comment about parallel labelling experiments and their effectiveness in higher observability of the metabolic network.

Indeed, parallel labelling experiments have become popular in ^{13}C-MFA, aiming to improve the quality of flux inferences (10.1016/j.ymben.2012.11.010). Here, multiple labelling experiments are performed under precisely the same conditions, but with different tracers. If successful, the collected data sets are jointly evaluated, under the assumption that the underlying fluxes are the same in all cases.

The reviewer is right in that performing two experiments, one with a ^{13}C and one with a ^{15}N tracer, is an elegant way to separate the labelling contributions by ^{13}C and ^{15}N, (which could not be achieved with the measurement technique used in our work). There are two reasons, why we deliberately did not consider parallel experiments:

(1) For slow growing organisms, such as BCG and particularly Mtb, stationary ^{13}C-MFA experiments are time consuming. For instance, the bioreactor experiment conducted in this study was completed in approximately 4 months. To be able to assess biological variability, such an experiment should be performed in replicates. Biosafety reasons require cultivations to be performed in a laboratory 3 containment, implying sequential

experimentation, which multiplies the time investment of such experiments tremendously.

(2) As we pointed out in the introduction (Lines 64-65; lines 85-88), performing a ^{15}N experiment with a one-nitrogen source does only provide information, when executed as isotopically non-stationary experiment. In such an experiment, a series of time-dependent labelling enrichments is generated, which, together with the metabolic pool sizes, is informative about the fluxes. Establishing INST in BCG/Mtb, and in fact in any organism, comes with many additional challenges and uncertainties, which limits the application of this technique to a few expert groups worldwide. Since, INST ^{13}C-MFA has not been attempted in BCG/Mtb before, whether parallel ^{13}C, ^{15}N experiments are providing an information surplus compared to simultaneous $^{13}C^{15}N$ labelling experiments, is a question to be addressed in the future.

If such parallel experiments are practically feasible, the resulting data sets can be analysed in our proposed framework. In our work, as phrased by Reviewer 1, we decided for a low-demand experimental input, i.e., a single experiment and standard GC-MS analytics for amino acids, and show that even when unable to separate ^{13}C and ^{15}N contributions, informative flux results are obtained. In conclusion, we believe that our approach is viable and practical also for other organisms.

Reviewer 3 comment: The discussion of biological findings should be improved. First, the conclusion that at least one of the anaplerotic reactions runs in reverse has significant consequences. I am surprised that the authors are passing on the opportunity. Is it cycling thermodynamically feasible? Would a reversed anaplerotic reaction be able to support gluconeogenesis? This seems a novel finding across microorganisms.

Response 3.6: We thank the reviewer for this supportive comment.

We would like to clarify that mycobacteria including the pathogenic Mtb uses the anaplerotic node for gluconeogenic carbon assimilation and carboxylation. This has been well-studied in mycobacteria and other organisms by means of enzyme essentiality studies (10.1371/journal.ppat.1002091; 10.1074/jbc.RA118.001839; 10.3389/fbioe.2020.602936). These studies show that the enzymes of the Mtb's anaplaerotic node, including *pck*, *pca*, *ppdk*, *mez*, can in principle operate bidirectionally; the direction of flux or reversibility has been shown to be dependent on the carbon source. For example, *pck* is an enzyme that converts oxaloacetate to phosphoenol pyruvate for gluconeogenic carbon assimilation and for TCA cycle carbon replenishment; *mez* was essential for growth on glycolytic substrates. However, such studies are unable to reveal the in vivo anaplerotic operation modes or metabolic fluxes. For this, ^{13}C-MFA is required.

In ^{13}C MFA, it is common to set thermodynamic constraints (see Responses 2.3 and 3.4), which unfortunately often proofed less effective (10.1016/j.matcom.2010.10.025). Therefore, thermodynamic cycling of the anaplerotic fluxes cannot be excluded a priori, but has to be discussed with the flux

inferences at hand. For this, we have extended our discussion about the anaplerotic node and its relevance to Mtb's metabolism (lines 419-424).

Additions made in the revised manuscript are detailed in Response 2.2. We also changed the section heading to "BMA-based $^{13}C^{15}N$-MFA validates and refines carbon fluxes in mycobacteria and reveals new insights into the anaplerotic node" (Lines 200-201).

Reviewer 3 comment: Second, the conclusion that oleic acid affects lower glycolysis is merely speculative. What would be the mechanism? Beta-oxidation to AcCoA? Allosteric? Please prove it or tone down the statement.

Response 3.7: We agree with the reviewer that our inference on oleic acid and its effect on lower glycolysis does not rest on mechanistic knowledge. The mechanism of oleic acid assimilation is complex. It involves β-oxidation of fatty acids to generate acetyl coenzyme A that enters central carbon metabolism and lipid metabolism (10.1016/j.tube.2011.06.006). In Beste et al. (2011), we modelled the oleic acid assimilation into central carbon metabolism using a simplistic model in which oleic acid carbons are channelled into the central carbon metabolism as C2 AcCoA units. This simple model was useful and effective to explain the dilution of labelling from glycerol. We are therefore confident about the low *pdh* (pyruvate dehydrogenase) flux resulting in our former study.

With the revision, a new version of Fig. EV3 is provided showing relative fluxes and we have modified the text accordingly.

Changes made in the revised manuscript:

Lines 233-240.: Accentuated differences are found for the pdh flux in lower glycolysis. We attribute this discrepancy to the differences in the experimental setup between the two studies: in this study, tyloxapol was used as dispersant in the medium as replacement for tween-80 or oleic acid, which was a medium component in our previous study, and is known to be a carbon source for mycobacteria (Pietersen et al, 2020). By comparing the two flux analyses using tween or tyloxapol in the medium, we concluded that only discrepancies in lower glycolytic fluxes, but no "global" effects on central carbon metabolism were found under the investigated conditions.

Reviewer comment 3: Third, the new analysis reveals that the estimates provided by Beste 2011 were not correct. As this also the case for TCA cycle fluxes (sdh, fum, kor) and the pyruvate node, it would be sensible to elaborate whether the original conclusion about the relevance of the isocitrate lyase for pyruvate dissimilation is still valid.

Response 3.8: As we have detailed in Response 3.2 above, we argue that given the experimental variability, change in carbon source (directly affecting the pyruvate

node), differences in model resolution and biomass formulation, different data quality and inference paradigms, the mentioned discrepancies in the three TCA fluxes should be considered minor. Most fluxes do agree and especially the isocitrate lyase flux, mentioned by the reviewer, is very similar. Also, the GAS pathway prominently emerged from both studies. We have made this clear in the revised manuscript (see Response 2.1).

Minor points:

Reviewer comment 3: I could not follow the argument on high resolution vs low resolution MS. The authors used low resolution instruments, which seems to be enough in this case. However, they state "that FT instruments operate at a tradeoff between resolving power and acquisition accuracy" (lines 146-147). I disagree: there is a tradeoff between resolution and speed, but not on the accuracy of isotopic ratios. Please clarify or provide compelling references.

Response 3.9: We agree with the reviewer and have made this statement clear in the text:

Changes made in the revised manuscript:

Lines 151-152: In practice, however, those analytical platforms operate at a trade-off between resolving power and acquisition speed.

Reviewer comment 3: The concept of "carbon-nitrogen flux" is confusing. The term is used to indicate something that differs from carbon and nitrogen fluxes (for example, in the abstract), even though it is just a combination of the two. Please avoid and rephrase.

Response 3.10: We have now rephrased carbon and nitrogen as the CN or C and N fluxes instead of carbon-nitrogen, clarified the text and modified it throughout the manuscript.

Reviewer comment 3: Line 379: Given the uncertainty shown in Figure 4 and S4, it doesn't seem correct to claim that "13C15N-MFA provides an effective tool to constrain the anaplerotic net fluxes".

Response 3.11: The (1D) CrIs anaplerotic fluxes are ranging from (roughly) -0.06 to +0.06 mmol/(g biomass h)$^{-1}$. With interpreting Fig. 4, notice that the CrIs or three anaplerotic fluxes, although having visually the largest among all fluxes (as a result of the bisymmetric log-transformed flux scale), their ranges in absolute terms in fact smaller or not much larger than some fluxes in the glycolysis or TCA (visible e.g. in Fig. S03)

.

An alternate way to assess the information about fluxes contained in the data set is to compare the stoichiometrically feasible flux ranges (blue histograms) with the 95% credibility intervals resulting from $^{13}C^{15}N$-MFA (orange histograms). The following plots help to put these distributions in perspective:

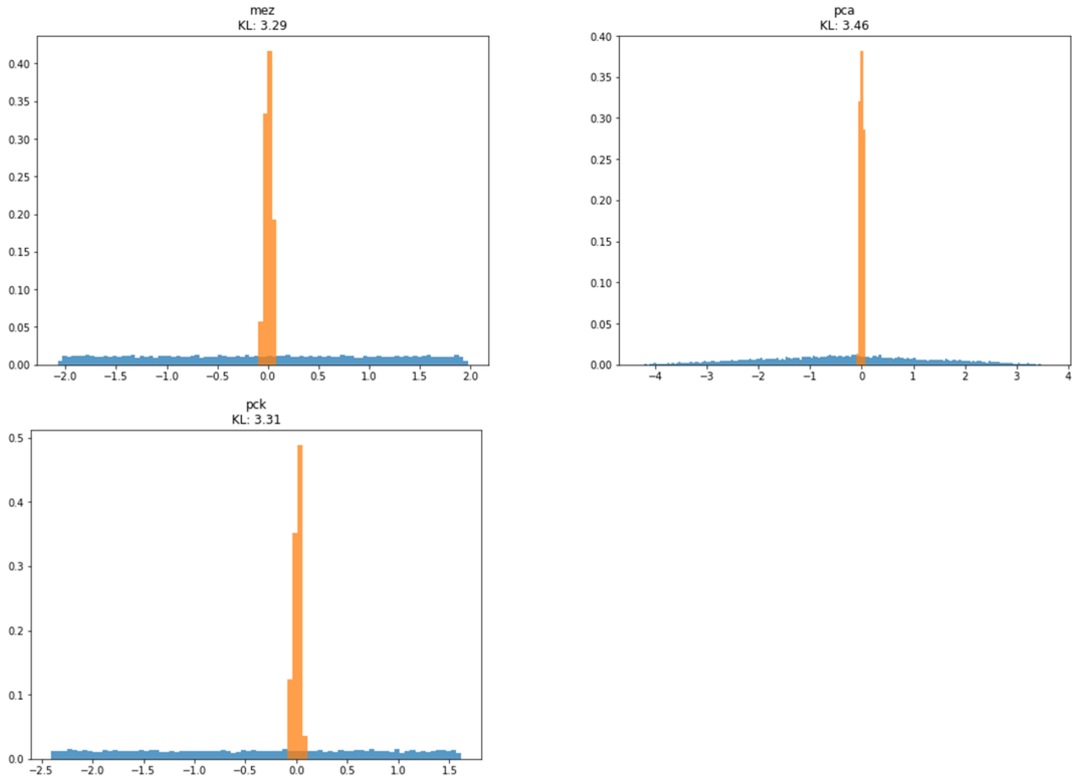

Here, the “contraction” is clearly visible (and formally measured using the Kullback-Leibler (KL) divergence. Consequently, and we conclude that the given $^{13}C^{15}N$ data set, evaluated using BMA-based MFA, effectively constrains the anaplerotic fluxes. This has also to be seen in the context that typically the anaplerotic fluxes are not well resolved.

We did not add the plots to the supplement (but are willing to do so, if the reviewers and editors should find them useful) because, when turning to the 2D credible regions in Figure 5 and Figure S4, we see that much more information about the fluxes is contained in these plots than in the marginal posterior plots above. With these 2D plots we are able to reveal active operation modes of the anaplerotic node (see Response 2.2). This highlights the huge potential of Bayesian analysis in the ^{13}C-MFA field. Indeed this is the first study exploiting this potential.

We extended the text to clarify that it is the data that effectively constrains the anaplerotic net fluxes and it is the Bayesian analysis that brings these constraints /nonlinear correlations to light (see Response 2.2).

Reviewer 3 comment: The method section on "Mass spectrometry analysis of amino acids" contains information on collecting high-resolution data with an Orbitrap, but nothing is mentioned in the main text. It also references a figure S7, which is wrongly labelled. Remove everything or embed it properly.

Response 3.12: We apologise that the use of orbitrap was not clear for the reviewer. We used it in the main text (materials and methods) to check the dual ^{13}C and ^{15}N label incorporation in the metabolites. Figure EV7 is labelled as "$^{13}C^{15}N$ labelling of alanine in M. bovis BCG chemostat cultures measured using orbitrap MS" that is embedded in the text (Lines 568-569).

Reviewer comment 3: A gltBD mutant is mentioned in the methods, but it wasn't used in the study.

Response 3.13: We have used the *gltBD* (BCG glutamate auxotroph) mutant to test the glutamate auxotroph's growth on various amino acids as nitrogen sources. We demonstrated that major predicted nitrogen sources aspartate, glutamine and ammonium chloride cannot rescue the growth of *gltBD* except for glutamate.

Lines detailing the gltBD use in the manuscript:

Lines 328-334: The centrality of this node for N assimilation was experimentally confirmed by examining substrate utilization of a glutamate auxotroph of *M. bovis* BCG with a transposon mutation in gltBD, a gene encoding glutamine oxoglutarate aminotransferase (GOGAT) that catalyzes the synthesis of GLU from OXG and GLN (Viljoen *et al*, 2013). Whereas the wild type M. bovis BCG strain could grow with GLYC as sole C and NH3, ASP, GLU and GLN as sole N sources (slope m >0), the gltBD mutant was able to grow only on glutamate as the N source (Fig. 7).

Reviewer comment 3: The biomass requirements should be reported somewhere.

Response 3.14: We have provided the following biomass equation in the main text supplementary Table EV2.

0.0067803 * ALA + 0.0040062 * ARG + 0.0011721 * ASP + 0.003003 * ASN + 0.0005139 * CYS + 0.001584 * GLU + 0.0024291 * GLN + 0.0046779 * GLY + 0.0011835 * HIS + 0.0021582 * ILE + 0.0049821 * LEU + 0.0010803 * LYS + 0.0009768 * MET + 0.0014886 * PHE + 0.0030279 * PRO + 0.0028773 * SER + 0.0030288 * THR + 0.0007701 * TRP + 0.001077 * TYR + 0.004521 * VAL + 0.0009186 * AMP + 0.0016929 * CMP + 0.00169635 * GMP + 0.0003456 * TMP + 0.0005424 * UMP + 0.0281823 * G6P + 0.0002811 * THF + 0.0093738 * R5P + 0.0011748 * PYR + 0.2955318 * ACCOA + 0.0007923 * GAP

1st Revision - Editorial Decision 7th Oct 2022

Thank you again for sending us your revised manuscript. We have now heard back from the two reviewers who were asked to evaluate your revised study. As you will see below, while reviewer #1 is satisfied with the performed revisions, reviewer #3 still raises concerns on the study. During our reviewer cross-commenting process (in which the reviewers are given the chance to comment on each other's reports), reviewer #1 mentioned: "My review for the revision was positive because I think that the BMA method is probably a milestone in the field of metabolic flux analysis. A big issue with the traditional MFA method is that the error analysis is not reliable. BMA provides a rigorous framework to address this problem. On the other hand, regarding the "one-shot" tracing strategy, I think reviewer #3 raised very good questions. Specifically regarding his/her two main questions, it is not clear to me either how the "one-shot" tracing is better than 1) 13C tracing alone or 2) separated 13C and 15N tracing. As the "one-shot" tracing is the main new result in this manuscript, in line with reviewer #3, I think it's important to have these questions addressed. Based on the authors' responses, it is possible that they are able to do so." Taken together and given that addressing the remaining concerns of reviewer #3 on the superiority of the proposed method are key for the conclusiveness of the study, we have decided to give you the chance to address them, in an exceptional second round of major revision.

These remaining concerns would need to be convincingly addressed. We recognize that this may involve substantial further experimentation and analyses with unclear outcome. Of course, we would understand if in light of these required revisions, you might choose to submit your study elsewhere.

We would also ask you to address some remaining editorial issues listed below.

Reviewer #1:

Thank the authors for explaining the BMA framework and referring to previous publications. Though now I realized that this manuscript is more of an application (than a development) of the BMA MFA, I think it demonstrates the power of this state-of-the-art flux analysis approach. Specifically, the approach is superior to the current MFA in 1) flux error analysis, 2) correlation analysis between fluxes, and 3) treatment of reversibility of reactions. Though computationally more expensive, it is a big step forward in flux analysis and I highly recommend the publication of the manuscript.
I encourage the authors to share the code/implementation of the BMA MFA. I did not find it in their earlier publications (Teorell et al. 2017, Teorell et al. 2020). It'd be a great contribution to the field if this powerful approach can be widely disseminated.

Reviewer #3:

The revised version of the manuscript highlights the technical aspects of combined 13C and 15N tracing for the analysis of microbial metabolism. The biological results remain of marginal relevance compared to any previous 13C-only traditional MFA approach. The only "new" finding is about an anaplerotic enzyme working in the gluconeogenic direction, but this was already known (see the 3 papers cited in Response 3.6). No novel insights emerge for N fluxes.

Hence, the novelty and relevance of this submission are bound to the use of one-shot 13C & 15N flux analysis. If this is the focus, there are some key questions that remain open:
(i) Is the one-shot 15N and 13C tangibly more informative than 13C alone?
I understand now that taking the 13C data from Beste 2011 isn't a viable option (Response 3.2). Nevertheless, comparing 15N13C-BMA with 13C-MFA doesn't allow to comment on the importance of the coupled labeling.

(ii) Is the one-shot 15N and 13C tangibly less informative that separated 15N and 13C experiments?
Response 3.5 brings justifications on why they didn't do it, both of which are weak. First, a BCG chemostat with D = 0.03 1/h takes 2-3 weeks (based on methods), not four months. Second, there is no need to do an INST-15N experiment. They question is whether parallel stationary 15N and 13C experiments provide equal or more information when used concomitantly to calculated fluxes. In any case, the question remains open.

Further points:
(iii) The reference to Mtb in the abstract should be removed. It signals that the study was done with Mtb, which is false. The abstract should unambiguously state that the whole analysis was done with M. bovis BCG strains.

(iv) In the last sentence of the abstract, it's hard to buy that the presented approach is a "low demand platform". As the authors mentioned in their rebuttal, the experiments took four months. Also, they seem to neglect the mathematical complexity. Such an analysis requires technical mastery and exceptional expertise. This seems the opposite of "low demand platform".

Responses to Reviewer comments': MSB-2022-11099R, One-shot $^{13}C^{15}N$-metabolic flux analysis for simultaneous quantification of carbon and nitrogen flux

Dear Maria,

Thank you for providing us with the review of our revised manuscript. We value the editorial and reviewers' comments which have helped us to further strengthen the manuscript mainly the results and validations. We have addressed reviewers' questions and the editorial comments in details for the revised submission. We have done additional analyses to validate the technical aspects of our one-shot $^{13}C^{15}N$ BMA platform. Please find below the detailed response to the two reviewers' comments. We hope that you will be able to consider our second round of revisions and publish our manuscript with Molecular Systems Biology.

Reviewer#1 comment:
Thank the authors for explaining the BMA framework and referring to previous publications. Though now I realized that this manuscript is more of an application (than a development) of the BMA MFA, I think it demonstrates the power of this state-of-the-art flux analysis approach. Specifically, the approach is superior to the current MFA in 1) flux error analysis, 2) correlation analysis between fluxes, and 3) treatment of reversibility of reactions. Though computationally more expensive, it is a big step forward in flux analysis and I highly recommend the publication of the manuscript.
I encourage the authors to share the code/implementation of the BMA MFA. I did not find it in their earlier publications (Teorell et al. 2017, Teorell et al. 2020). It'd be a great contribution to the field if this powerful approach can be widely disseminated.

Response: We thank the reviewer for the positive comments and appreciation of our work. BMA for ^{13}C-MFA currently requires a license of 13CFLUX2 (doi: 10.1093/bioinformatics/bts646), which is available from FZJ for academic and teaching purposes free of charge. For this reason, we are allowed to share the software only within verifiable settings. To disseminate our approach to the broad public, we are working with high priority on an open-source successor software of 13CFLUX2, which will contain BMA-based $^{13}C^{15}N$-MFA, as one of several isotope-based MFA variants.

Reviewer#3:
The revised version of the manuscript highlights the technical aspects of combined 13C and 15N tracing for the analysis of microbial metabolism. The biological results remain of marginal relevance compared to any previous 13C-only traditional MFA approach. The only "new" finding is about an anaplerotic enzyme working in the gluconeogenic direction, but this was already known (see the 3 papers cited in Response 3.6). No novel insights emerge for N fluxes.

Response i) We thank the reviewer for reviewing our revised manuscript and for the constructive feedback. We would like to point out that the measurement of nitrogen metabolic fluxes in mycobacteria is entirely novel and its combination with carbon flux analysis is the main focus of this work. This extends the traditional ^{13}C- or ^{15}N-MFA approaches to new areas; previously the application of mono-isotopic tracers in traditional ^{13}C-MFA or ^{15}N-MFA were not able to resolve nucleotide metabolism, but our BMA approach with dual-isotopic tracers resolves for the first time, fluxes for nucleotide metabolism in *Mycobacterium bovis* BCG.

As the reviewer pointed out earlier, a simple flux balance analysis (FBA) allows predicting these fluxes under some evolutionary argument. However, using FBA, the resulting flux predictions are fixed values dictated by their contributions to biomass. In our isotope labelling approach, biomass contributions are specified as a measurement, respecting their uncertainty (see e.g. doi: 10.1186/s13059-021-02289-z explaining why this is relevant) which is in contrast to the FBA approach. The uncertainty in the biomass contribution, together with the uncertainty in the remaining data and the model (i.e., reaction

bidirectionalities), is then propagated for flux estimation. The emerging flux posterior distribution allows for the statistical assessment of how likely the fluxes are in the context of the biochemical CN network. Despite considering these unknowns, we were able to shed light on the anaplerosis, being a unique feature of the BMA-based ^{13}C^{15}N-MFA. For example, our previous ^{13}C-MFA was unable to predict flux correlations; using BMA approach we are now able to confirm the operation modes of pck/mez which are operating gluconeogenically during glycerol catabolism.

Obviously, the question arises how informative a simultaneous ^{13}C^{15}N labelling experiment can be in the best of all cases. There are more informative nitrogen-substrates that exist for mycobacteria than the ones applied in our study. For example, rather than the N_1-source ammonium chloride, which shows no information gain in the isotopically stationary labelling regime (see also response (iii) to Reviewer #3), a N_2-source such as glutamine (doi: 10.1016/j.celrep.2019.11.037) might provide more information about the nitrogen fluxes. Also, with regards to ^{13}C-labelled substrates, more informative choices are possible, i.e., positionally labelled glycerol. Ultimately, the same procedures being established for optimizing the ^{13}C tracer choice can be adopted to simultaneous ^{13}C-^{15}N tracer design (e.g., doi: 10.1002/(SICI)1097-0290(1999)66:2<86::AID-BIT2>3.0.CO;2-A; doi: 10.1371/journal.pcbi.1006533; doi: 10.3389/fbioe.2021.685323). But using other carbon/nitrogen sources than the glycerol/ammonium chloride would have firstly, complicated the comparison of ^{13}C^{15}N-BMA with the former ^{13}C-MFA work conducted with glycerol/ammonium chloride and, secondly, the question of experimental tracer design was outside the scope of this study (but the results are, of course, an excellent starting point for planning future labelling experiments. (e.g. https://doi.org/10.3389%2Ffbioe.2021.685323)).

We have highlighted the focus of our work in the revised manuscript (Lines 34-39). Our approach also provides novel insights into the nitrogen metabolism that has been very limitedly explored with standard ^{13}C-MFA. For example, in this work we identify glutamate as the central hub for nitrogen metabolism and not aspartate or glutamine, which had been previously proposed to be the principal nitrogen donors for intracellular replication of Mtb (doi: 10.3389/fcimb.2013.00068; doi: 10.1128/IAI.71.7.3927-3936.2003; doi:10.1016/j.celrep.2019.11.037). Our earlier work (doi:10.1016/j.celrep.2019.11.037) using ^{15}N-flux spectral ratio analysis identified the likely nitrogen sources including glutamine, glutamate, aspartate, alanine, valine acquired from the host cells. However, we were not able to measure the nitrogen fluxes, as this was not feasible with the experimental model and the flux modelling approach. So, we were unable to establish which of the five tested nitrogen donors was the source of the central nitrogen flux in *Mycobacterium tuberculosis* (Mtb).

In this new work, we developed BMA-based ^{13}C^{15}N-MFA to quantify both carbon and nitrogen fluxes simultaneously *in vitro*, delivering the first intracellular nitrogen flux measurements in mycobacteria. We demonstrate that glutamate, rather than glutamine, aspartate, etc., is the central hub (donor) for nitrogen metabolism in *in vitro*-grown Mtb and thereby identify the glutamate node as a primary target for drug development. In addition, and for the first time, we were also able to measure nitrogen fluxes towards nucleotide metabolism, another key pathway that requires both carbon and nitrogen fluxes and potential drug target. All of these are novel findings.

We therefore do not agree with the reviewer that the work is of only 'marginal significance' compared to ^{13}C-only approaches. The reviewer seems to focus only on the study's contribution to understanding carbon metabolism in Mtb, but that is only one part of our study. Nitrogen is just as essential as carbon for life but has largely been ignored in flux studies because of the difficulty of measuring nitrogen fluxes. For the first time, we measure nitrogen fluxes in the pathogen model of tuberculosis that kills more than a million people each year.

Reviewer#3:
Hence, the novelty and relevance of this submission are bound to the use of one-shot ^{13}C & ^{15}N flux analysis. If this is the focus, there are some key questions that remain open:
(i) Is the one-shot ^{15}N and ^{13}C tangibly more informative than 13C alone?
I understand now that taking the ^{13}C data from Beste 2011 isn't a viable option (Response 3.2).
Nevertheless, comparing ^{15}N^{13}C-BMA with ^{13}C-MFA doesn't allow to comment on the importance of the coupled labeling.

Response ii) We thank the reviewer for commenting on the importance of $^{13}C^{15}N$ labelling rather than ^{13}C alone.

To answer reviewer's question "Is the one-shot ^{15}N and ^{13}C tangibly more informative than ^{13}C alone? The first point is that ^{13}C labelling provides zero information about nitrogen fluxes; our method that provides both carbon and nitrogen fluxes in a one-shot experiment is clearly innovative.

To illustrate differential incorporation of isotopes in amino acids, we show data from three independent labelling experiments with *Mycobacterium bovis* BCG using (i) 12.5% ^{13}C-glycerol and naturally ^{14}N-ammonium chloride (condition (a)), (ii) naturally ^{12}C-glycerol and 20% ^{15}N-ammonium chloride (condition (b)), and (iii) 12.5% ^{13}C-glycerol + 20% ^{15}N-ammonium chloride (condition (c)). The experiments were performed in exponentially growing batch cultures. Cultures were sampled at late exponential growth stage consistent across the three labelling conditions so that the data can be compared across the three conditions. However, the batch labelling experiments were not performed at metabolic steady state conditions, implying that the labelling enrichments do not reflect an isotopically steady state. The data is therefore neither suitable for isotope-based MFA, nor comparable to the labelling data from the main experiment in the manuscript.

Figure 1 below shows a comparison of the batch labelling data for conditions (a) and (c). Using one-shot ^{13}C glycerol + ^{15}N ammonium chloride labelling, we demonstrate a significantly higher labelling enrichment (for carbon plus nitrogen) for the amino acids analyzed: alanine, serine, glutamate, aspartate, arginine, and histidine, compared to ^{13}C-only labelling. With one-shot ^{13}C + ^{15}N labelling, we obtain both carbon and nitrogen mass isotopomer data (C1, C1N1, C2, C2N1, C3, C3N1 and N1), which gives us access to the nitrogen fluxes beyond the carbon fluxes that can be derived from a ^{13}C-labeling experiment.

Note that we are not claiming in the paper that the $^{13}C^{15}N$ labelling method is superior to ^{13}C labelling alone for measuring carbon fluxes. Our point is the demonstration of a single-shot method for measuring both carbon and nitrogen fluxes in a single experiment, which is neither possible with ^{13}C alone, nor with two separated ^{13}C and ^{15}N experiments under isotopically stationary conditions (see below).

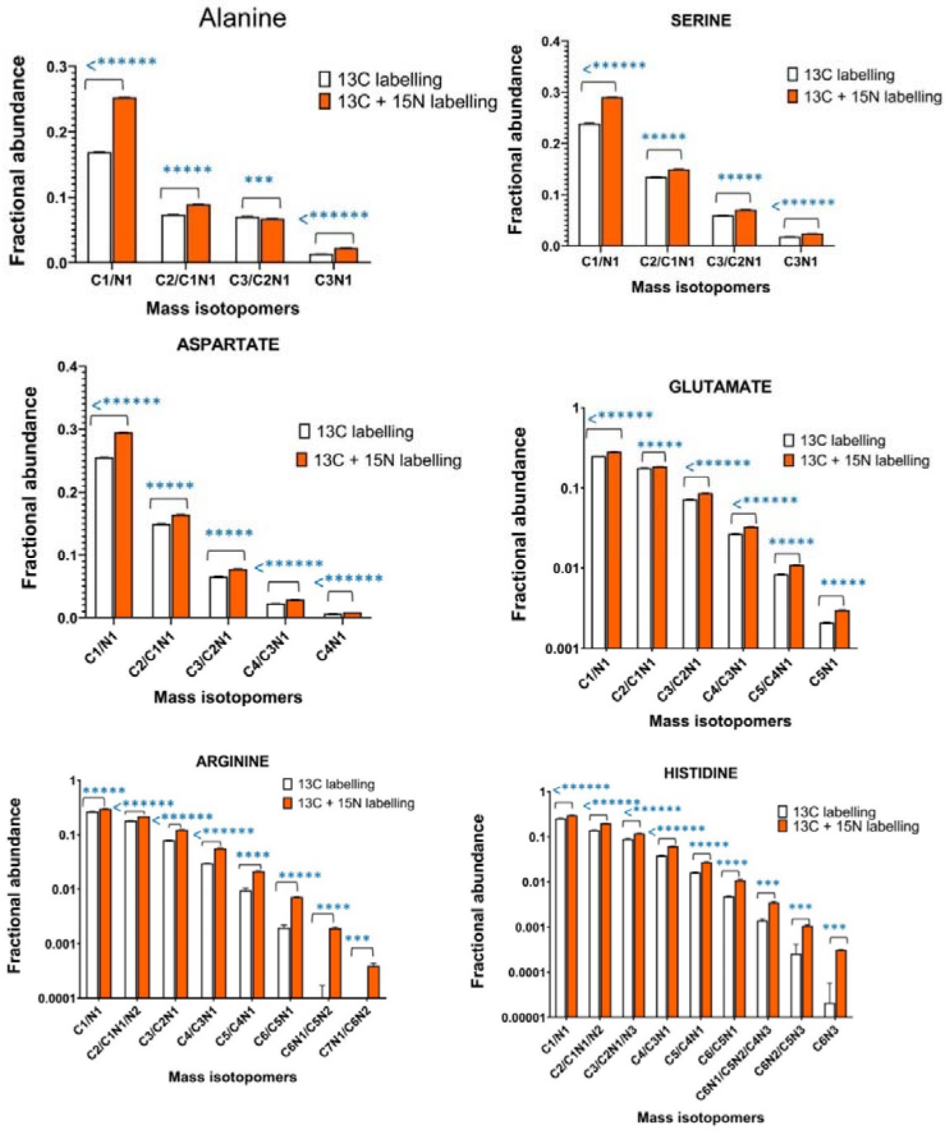

Figure 1. ^{13}C vs. $^{13}C^{15}N$ labelling incorporation in *Mycobacterium bovis* BCG. Cultures were grown as batch up to late exponential phase with either 12.5% ^{13}C-glycerol (condition a) or 12.5%^{13}C glycerol + 20% ^{15}N ammonium chloride in minimal media (condition c). Cultures were harvested for metabolomics and isotopic label analysis at late exponential growth stages. Data are shown for alanine, serine, aspartate, glutamate, arginine and histidine. Mass isotopomer distributions (MIDs) are shown for carbon (C) and nitrogen (N) atoms. Values are mean ± S.D. (n=3). Statistical analyses was done with t-tests (Graph pad prism);* indicates statistically significant differences; <******,p<0.0000005;***,p<0.0005.

Reviewer#3:

(ii) Is the one-shot ^{15}N and ^{13}C tangibly less informative that separated ^{15}N and ^{13}C experiments? Response 3.5 brings justifications on why they didn't do it, both of which are weak. First, a BCG chemostat with D = 0.03 1/h takes 2-3 weeks (based on methods), not four months. Second, there is no need to do an INST-^{15}N experiment. They question is whether parallel stationary ^{15}N and ^{13}C experiments provide equal or more information when used concomitantly to calculated fluxes. In any case, the question remains open.

Response iii) Our response to the reviewer's question "Is the one-shot 15N and 13C tangibly less informative that separated 15N and 13C experiments"? is below:

The data from the batch BCG labelling experiments described above are used to compare ^{13}C-glycerol alone (condition (a)), ^{15}N-ammonium chloride alone (condition (b)) and ^{13}C-glycerol + ^{15}N-ammonium chloride labelled substrates (condition (c)). Figure 2 below shows the isotopologue information provided for each of the labelling conditions.

For example, alanine has 3 carbons and 1 nitrogen; so in the best case 4 multivariate (CN) isotopologues contribute information about carbon and nitrogen fluxes. With ^{13}C-glycerol as the only labelled species (condition (a)), we derive no nitrogen labelling data. For a labelling strategy with only ^{15}N-ammonium chloride (condition (b)) we derive no information on the carbon labelling data. Our one-shot method with ^{13}C-glycerol + ^{15}N-ammonium chloride dual labelling experiment, we have information on both carbon and nitrogen labelling (for the alanine example, M1 (1 labelled carbon/ 1 labelled nitrogen), M2 (two labelled carbons/ 1 labelled carbon + 1 labelled nitrogen), M3 (3 labelled carbons/ 2 labelled carbons + 1 labelled nitrogen) and M4 (3 labelled carbons + 1 labelled nitrogen)).

To complement the lab experiments with a simulation experiment, we used representative samples from the posterior flux information, obtained with BMA-based $^{13}C^{15}N$-MFA, and predicted the isotopic steady-state labelling patterns for three conditions (a) 20% [$^{13}C_3$] glycerol + 0% [$^{15}N_1$] ammonium chloride, (b) 0% [$^{13}C_3$] glycerol + 12.5% [$^{15}N_1$] ammonium chloride, and (c). 20% [$^{13}C_3$] glycerol + 20% [$^{15}N_1$] ammonium chloride.

Figure 3 below shows the predicted isotopologue values with their associated 95% credible intervals. We do not aim to discuss the discrepancy between Figure 2 and 3 here, because the data sets were obtained by incompatible settings (described before). However, one fact should be discussed: With regard to nitrogen, the simulated data is lacking an error bar. The reason is that all simulated values are identical. Indeed the nitrogen fractional enrichment is computable by a simple binomial formula. For instance, for arginine (N4) an ammonium chloride label of 87.5% ^{14}N and 12.5% ^{15}N, gives for the N0 measurement $\binom{4}{0}\, 0.125^0\, (1-0.125)^4 \approx 0.586$ [-], for N1 $\binom{4}{1}\, 0.125^1\, (1-0.125)^3 \approx 0.335$ [-], for N2 $\binom{4}{2}\, 0.125^2\, (1-0.125)^2 \approx 0.072$ [-], for N3 $\binom{4}{3}\, 0.125^3\, (1-0.125)^1 \approx 0.007$ [-], and for N4 $\binom{4}{4}\, 0.125^4\, (1-0.125)^0 \approx 0.0002$ [-]. With other words, isotopically stationary ^{15}N-labelling data do not contain any information about C, N or CN fluxes.

Together, this explains that our one-shot $^{13}C^{15}N$ analysis is more than the sum of its parts. It is more informative than separated ^{15}N and ^{13}C experiments because it reports on the connectivity between carbon and nitrogen metabolism, something that independent (isotopically stationary) experiments cannot provide.

With the revision, we added Figures S8 (Figure 2 of the revision) and S9 (Figure 3 of the revision) in appendix to show the principal isotopologue information provided by each of the labelling conditions (pre-experiments), and the labelling enrichments that could be expected from experiments under metabolic and isotopic steady state conditions, under the labelling strategies (a), (b), (c). In addition, we extended the materials and methods section accordingly (Lines 533-537).

Also from a practical standpoint our on-shot $^{13}C^{15}N$ labelling strategy is superior to the separate ^{13}C or/and ^{15}N labelling experiments, because 1) reduces the labour intensive chemostat set up and analysis. For instance, with one-shot dual labelling set up, we completed our experimental analysis within four months; this includes two independent chemostats. Separate ^{13}C and ^{15}N labelling strategies with two independent replicates doubles the experimental time duration to 8 months; 2) the one-shot strategy reduces contamination issues that maybe associated with multiple chemostats for separate labelling experiments.

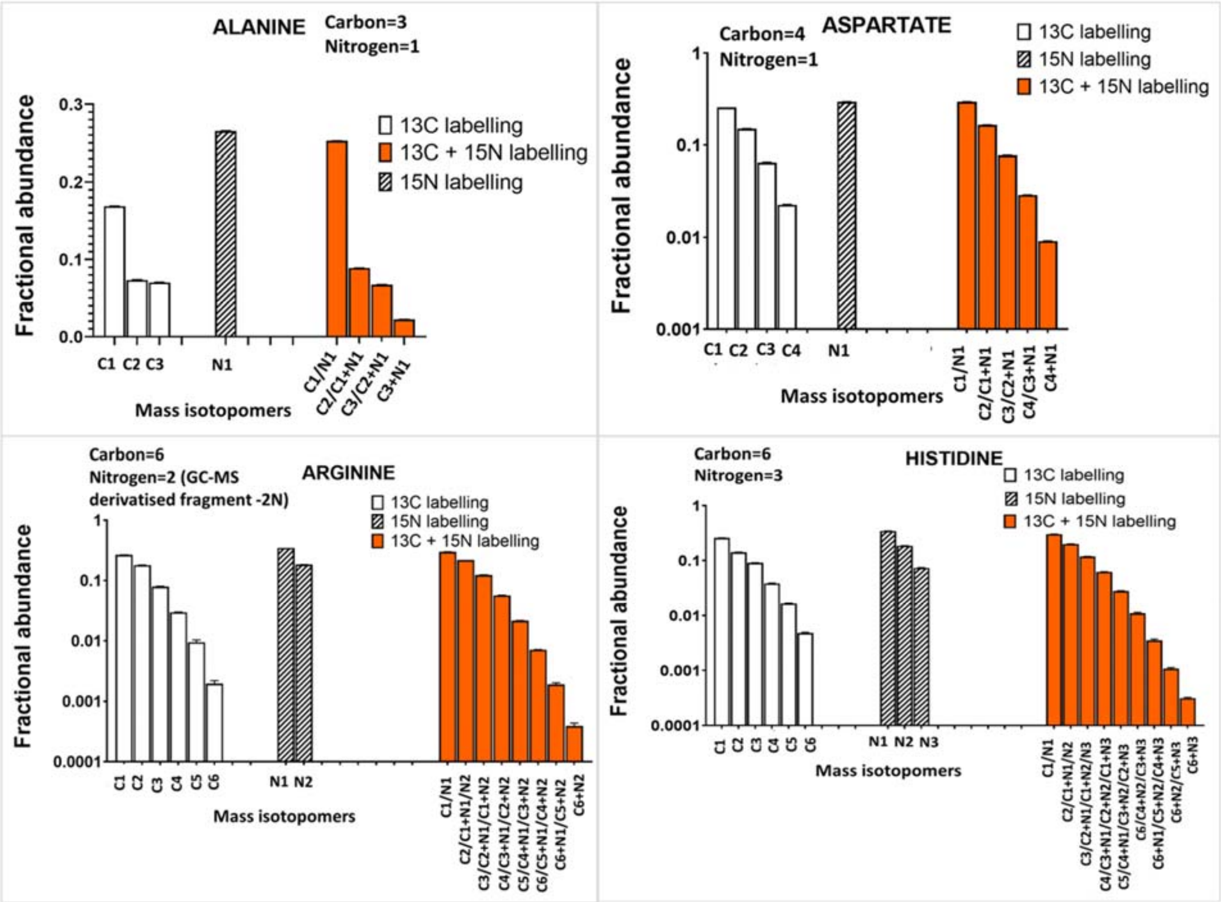

Figure 2. Mass isotopomer distributions for amino acids alanine, serine, arginine and histidine obtained from ^{13}C-glycerol (condition (a)), ^{15}N-ammonium chloride (condition (b)) and ^{13}C-glycerol + ^{15}N-ammonium chloride (condition (c)) labelling. Datasets show the comparisons between the three labelling conditions. ^{13}C-glycerol (condition (a)) labelling experiment provides only carbon (C) data. ^{15}N-ammonium chloride (condition (b)) labelling experiment provides only nitrogen (N) data. ^{13}C-glycerol + ^{15}N-ammonium chloride (condition (c)) labelling experiment provides both C and N (C + N) data. C1, C2, C3…C6 are carbon mass isotopomers; N1, N2, N3 are nitrogen mass isotopomers and C1+N1…..C6+N1, C6+N2, C6+N3 are carbon + nitrogen isotopologues. Values are mean ± S.D. (n=3).

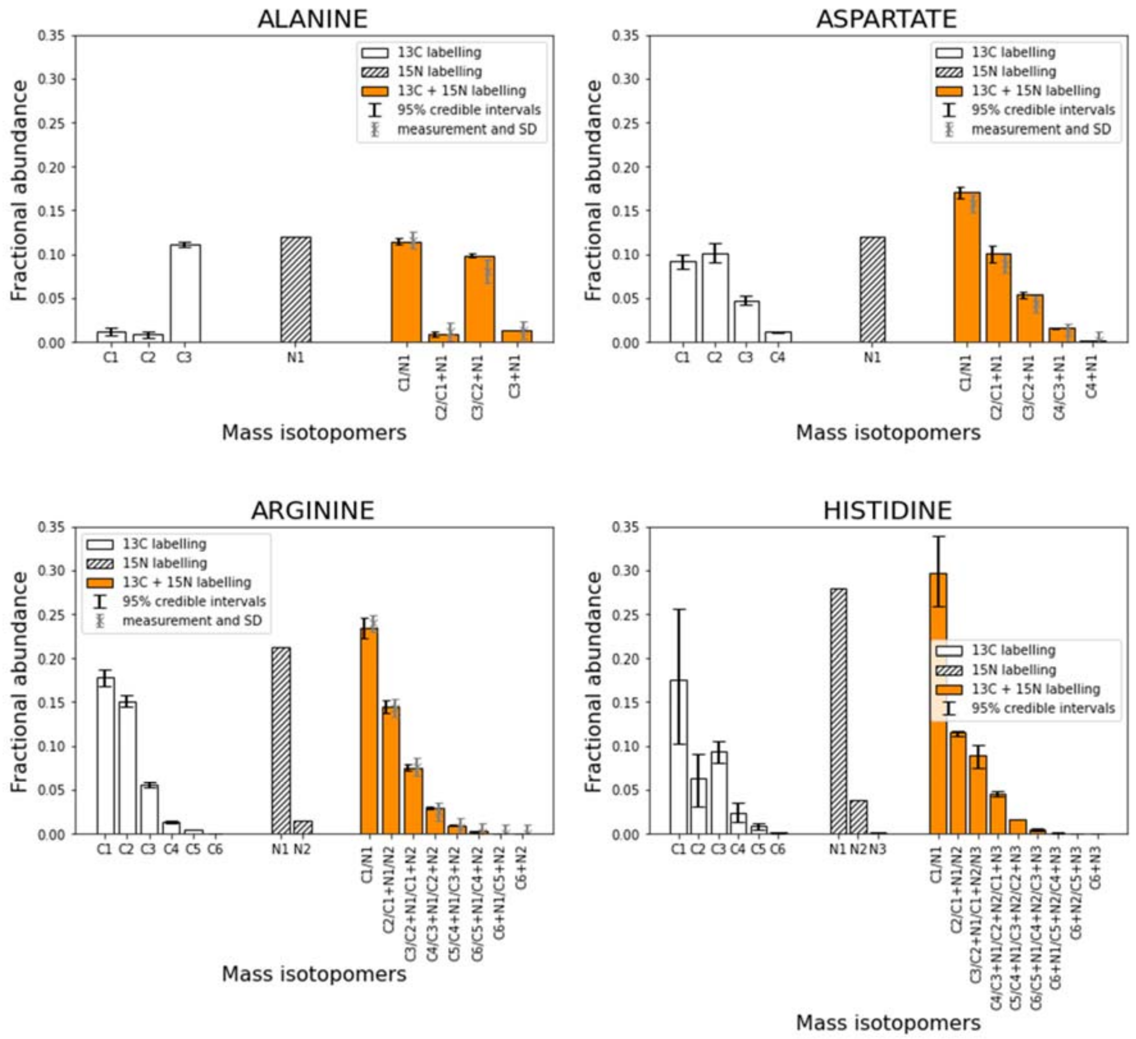

Figure 3. Simulated mass isotopomer distributions for amino acids alanine, serine, arginine and histidine obtained by sampling posterior distributions obtained from BMA-based $^{13}C^{15}N$-MFA at metabolic and isotopic stationarity for conditions (a) 12.5% [$^{13}C_3$] glycerol + 0% [$^{15}N_1$] ammonium chloride, (b) 0% [$^{13}C_3$] glycerol + 20% [$^{15}N_1$] ammonium chloride, and (c) 12.5% [$^{13}C_3$] glycerol + 20% [$^{15}N_1$] ammonium chloride. The value given represents the expected fractional enrichment, the error bars represent the 95% credible intervals. For the co-labelling strategy, observed data are given in addition.

Further points:
(iii) The reference to Mtb in the abstract should be removed. It signals that the study was done with Mtb, which is false. The abstract should unambiguously state that the whole analysis was done with M. bovis BCG strains.

Response: We agree with the reviewer and have clarified the above point (Lines 26, 28).

(iv) In the last sentence of the abstract, it's hard to buy that the presented approach is a "low demand platform". As the authors mentioned in their rebuttal, the experiments took four months. Also, they seem to neglect the mathematical complexity. Such an analysis requires technical mastery and exceptional expertise. This seems the opposite of "low demand platform".

Response:

We thank the reviewer for appreciating the technical and experimental expertise required to accomplish this new platform. Indeed BMA-based $^{13}C^{15}N$-MFA is not low-demand when compared to other bioassays. But, among the ^{13}C-MFA variants, a combination of GC-MS with a single isotopic labelling experiment is indeed one of the experimentally cheapest approaches.

The dual labelling strategy we demonstrated here significantly reduces the amount of labour-intensive work that would have been required for separate labelling experiments. For example, if two chemostats require two months each for dual species labelling, separate labelling strategy would require 8 months. So, by devising this platform of one-shot BMA-based $^{13}C^{15}N$-MFA we developed a versatile, less labour demanding experimental set up, standard analytical GC-MS data analysis and a statistically robust platform for simultaneous carbon-nitrogen flux measurements in any system.

As per this reviewer's suggestion, we have modified the abstract as below: Lines 37-38.

Editorial issues:

- In line with the comment of reviewer #1, we would ask you to share the code of BMA MFA on GitHub.

Response: BMA-based ^{13}C-MFA currently requires a license of 13CFLUX2 (doi: 10.1093/bioinformatics/bts646), which is available from Forschungszentrum Jülich for academic and teaching purposes free of charge. For this reason, we are allowed to share the software only within verifiable settings. To disseminate our approach to the broad public, we are currently working with high priority at an open-source successor software of 13CFLUX2, which will contain BMA-based $^{13}C^{15}N$-MFA, as one of several isotope-based MFA variants.

- In the Data Availability section, please indicate how the data and code has been made available (e.g. EV Table, EV Dataset etc.)

Response: we have now added this information. Lines 514-515.

- Please format the References according to the journal reference style i.e. ordered alphabetically and not in numbered order

Response: We have updated the format of reference list

- Fig 5C should be called out before 5D.

Response: In the revised version, Fig 5C is called before Fig 5D. Lines 277-279.

- Table S1 is called out, but there is no Table S1, please correct this.

Response: We have corrected Table S1 to Table EV1.

- Currently there are 10 EV figures, and we typically do not allow more than 6. We would ask you to include all these figures can be included in a PDF called Appendix. Appendix figures should be labeled and called out as: "Appendix Figure S1, Appendix Figure S2... Appendix Table S1..." etc. Each legend should be below the corresponding Figure/Table in the Appendix. Please include a Table of Contents in the beginning of the Appendix. For detailed instructions regarding expanded view please refer to our Author Guidelines: http://msb.embopress.org/authorguide#expandedview.

Response: We have included all figures into a single pdf.

- The legend for Table 1 needs to be added to the file for Table 1.

Response: We have included Table 1 in the main text and submitted as a separate word file.

- Our data editors and I have made some edits/comments in the text, including the figure legends, please see the attached .doc file. Please make all requested text changes using the attached file and *keeping the "track changes" mode* so that we can easily access the edits made.

Response: We have made edits and revisions to the file you were editing, and track changes mode is on.

- The provided synopsis image is rather detailed and the labelling does not read very well at the required final size (width = 550 px). Please provide an updated synopsis exactly at 550 px width (the height does not matter) and make sure that all labelling is easily readable.

Response: We have provided a new synopsis figure

- At EMBO Press we ask authors to provide source data for the main and EV figures. Our source data coordinator will contact you to discuss which figure panels we would need source data for and will also provide you with helpful tips on how to upload and organize the files.

Response: We look forward to receiving these instructions from the journal.

2nd Revision - Editorial Decision 13th Dec 2022

Thank you again for sending us your revised manuscript. We have now evaluated the revised manuscript and your responses to the reviewers' comments. We think that most of the reviewers' concerns have been satisfactorily addressed. However, we noted that the comment of reviewer #1 regarding the absence of code/implementation of BMA for 13C-MFA has not been addressed. We do acknowledge that the fact that 13CFLUX2 is published under a commercial license (and a non-commercial license for academic use) does not allow sharing the software freely. However, we also noted that 13CFLUX2 is mainly used for likelihood computation. Perhaps these results could be presented as a table? We feel that overall, the description of the 13C15N-Metabolic flux analysis as it stands does not seem sufficiently detailed for reproducing the analyses performed in the paper and/or applying the approach to another biological context. This is not ideal, as the BMA for 13C-MFA workflow is integral to the study and, as reviewer #1 pointed out, it is important to make it accessible to the readers. For transparency and for ensuring long term archival, we would ask you to include code and/or scripts as much as possible. They can be provided as Computer Code EV1, including a README.txt file with further instructions and information if required. This hopefully can be done for several parts of the computational analyses, while respecting the restrictions related to 13CFLUX2. Moreover, we would ask you to provide the metabolic model of M. bovis BCG that was used, ideally in SBML. Please include the relevant information in the Data Availability section. I have included a comment in the attached manuscript text file.

Responses to Reviewer comments': MSB-2022-11099R, One-shot $^{13}C^{15}N$-metabolic flux analysis for simultaneous quantification of carbon and nitrogen flux

Dear Maria,

Thank you for providing us with the review of our revised manuscript. We value the editorial and reviewers' comments which have helped us to further strengthen the manuscript mainly the results and validations. We have addressed the remaining reviewer 1's query and the editorial comments in details for this new revised submission. We have now provided the codes and scripts required to reproduce the BMA MFA analysis for anyone who has access to 13CFLUX2. Please find below the detailed response to the reviewer 1. We hope that you will be able to consider our third round of revisions and publish our manuscript with Molecular Systems Biology.

Reviewer#1 comment:
Thank the authors for explaining the BMA framework and referring to previous publications. Though now I realized that this manuscript is more of an application (than a development) of the BMA MFA, I think it demonstrates the power of this state-of-the-art flux analysis approach. Specifically, the approach is superior to the current MFA in 1) flux error analysis, 2) correlation analysis between fluxes, and 3) treatment of reversibility of reactions. Though computationally more expensive, it is a big step forward in flux analysis and I highly recommend the publication of the manuscript.

I encourage the authors to share the code/implementation of the BMA MFA. I did not find it in their earlier publications (Teorell et al. 2017, Teorell et al. 2020). It'd be a great contribution to the field if this powerful approach can be widely disseminated.

Response: We thank the reviewer for the positive comments and appreciation of our work. BMA for ^{13}C-MFA currently requires a license of 13CFLUX2 (doi: 10.1093/bioinformatics/bts646), which is available from FZJ for academic and teaching purposes free of charge. For this reason, we are allowed to share the software only within verifiable settings. To disseminate our approach to the broad public, we are working with high priority on an open-source successor software of 13CFLUX2, which will contain BMA-based $^{13}C^{15}N$-MFA, as one of several isotope-based MFA variants.

In our revised manuscript, we have included the following repositories that provides access to the research community to our codes and scripts.

(i) We have provided a compiled binary that runs the type of analyses reported in the manuscript as an extension to a (functional) 13CFLUX2 installation. Since the samples from which the flux map is derived has a size of 5GB, these cannot be presented as a table. We have therefore provided step-by-step for reproduction of our BMA analysis instead.
We have created a github repository that contains all the scripts to execute the BMA pipeline with the model, which should allow anyone who has access to 13CFLUX2 to reproduce the computations. The public repository is provided at the following URL. https://github.com/JuBiotech/Supplement-to_Borah-et-al.-MSB-2023.

This repository contains:

- compiled BMA extension to 13CFLUX2 (ver >=2.3)
- script with the used parameters for x3cflux-bma (as used in the study)
- random seeds for the MCMC chains (for reproduction of results)
- FluxML model file of M. bovis BCG containing reactions, CN transitions, constraints and data

(ii) In addition, the scripts and the model file are also archived on Zenodo (https://doi.org/10.5281/zenodo.7506282).

Please see the updated data availability section in the revised manuscript (lines 699 to 701) that provides link to the GitHub repository and zenodo. We have also added the Github and Zenodo links in the Materials and methods section (lines 651 to 653) for reproducing the BMA analyses.

Editorial comments: Moreover, we would ask you to provide the metabolic model of M. bovis BCG that was used, ideally in SBML. Please include the relevant information in the Data Availability section. I have included a comment in the attached manuscript text file.

Response: FluxML is the de-facto standard to formulate models for isotope-based MFA and a format that is designed for transparency and reproducibility (10.3389/fmicb.2019.01022). SBML is not suitable here, since there is no standardized way to store atom transitions or labelling data. We have provided the FluxML file (data set EV1) and an excel file for reactions (Table EV2) for the M. bovis BCG model. Please see the data availability section.

3rd Revision - Editorial Decision 13th Jan 2023

Thank you again for sending us your revised manuscript and for fixing the last remaining issues regarding the code availability. We are now satisfied with the modifications made and I am pleased to inform you that your paper has been accepted for publication.

EMBO Press Author Checklist

Corresponding Author Name: Johnjoe McFadden	
Journal Submitted to: Molecular Systems Biology	
Manuscript Number: MSB-2022-11099R	

USEFUL LINKS FOR COMPLETING THIS FORM
The EMBO Journal - Author Guidelines
EMBO Reports - Author Guidelines
Molecular Systems Biology - Author Guidelines
EMBO Molecular Medicine - Author Guidelines

Reporting Checklist for Life Science Articles (updated January
This checklist is adapted from Materials Design Analysis Reporting (MDAR) Checklist for Authors. MDAR establishes a minimum set of requirements in transparent reporting in the life sciences (see Statement of Task: 10.31222/osf.io/9sm4x). Please follow the journal's guidelines in preparing your manuscript.
Please note that a copy of this checklist will be published alongside your article.

Abridged guidelines for figures
1. Data
The data shown in figures should satisfy the following conditions:
- the data were obtained and processed according to the field's best practice and are presented to reflect the results of the experiments in an accurate and unbiased manner.
- ideally, figure panels should include only measurements that are directly comparable to each other and obtained with the same assay.
- plots include clearly labeled error bars for independent experiments and sample sizes. Unless justified, error bars should not be shown for technical
- if n<5, the individual data points from each experiment should be plotted. Any statistical test employed should be justified.
- Source Data should be included to report the data underlying figures according to the guidelines set out in the authorship guidelines on Data

2. Captions
Each figure caption should contain the following information, for each panel where they are relevant:
- a specification of the experimental system investigated (eg cell line, species name).
- the assay(s) and method(s) used to carry out the reported observations and measurements.
- an explicit mention of the biological and chemical entity(ies) that are being measured.
- an explicit mention of the biological and chemical entity(ies) that are altered/varied/perturbed in a controlled manner.
- the exact sample size (n) for each experimental group/condition, given as a number, not a range;
- a description of the sample collection allowing the reader to understand whether the samples represent technical or biological replicates (including how many animals, litters, cultures, etc.).
- a statement of how many times the experiment shown was independently replicated in the laboratory.
- definitions of statistical methods and measures:
  - common tests, such as t-test (please specify whether paired vs. unpaired), simple χ2 tests, Wilcoxon and Mann-Whitney tests, can be unambiguously identified by name only, but more complex techniques should be described in the methods section;
  - are tests one-sided or two-sided?
  - are there adjustments for multiple comparisons?
  - exact statistical test results, e.g., P values = x but not P values < x;
  - definition of 'center values' as median or average;
  - definition of error bars as s.d. or s.e.m.

Please complete ALL of the questions below.
Select "Not Applicable" only when the requested information is not relevant for your study.

Materials

**Newly Created Materials**	**Information included in the manuscript?**	**In which section is the information available?** (Reagents and Tools Table, Materials and Methods, Figures, Data Availability Section)
New materials and reagents need to be available; do any restrictions apply?	Not Applicable	

**Antibodies**	**Information included in the manuscript?**	**In which section is the information available?** (Reagents and Tools Table, Materials and Methods, Figures, Data Availability Section)
For **antibodies** provide the following information:<br>- Commercial antibodies: RRID (if possible) or supplier name, catalogue number and or/clone number<br>- Non-commercial: RRID or citation	Not Applicable	

**DNA and RNA sequences**	**Information included in the manuscript?**	**In which section is the information available?** (Reagents and Tools Table, Materials and Methods, Figures, Data Availability Section)
**Short novel DNA or RNA including primers, probes**: provide the sequences.	Not Applicable	

**Cell materials**	**Information included in the manuscript?**	**In which section is the information available?** (Reagents and Tools Table, Materials and Methods, Figures, Data Availability Section)
**Cell lines:** Provide species information, strain. Provide accession number in repository **OR** supplier name, catalog number, clone number, and/**OR** RRID.	Not Applicable	
**Primary cultures:** Provide species, strain, sex of origin, genetic modification status.	Not Applicable	
Report if the cell lines were recently **authenticated** (e.g., by STR profiling) and tested for mycoplasma contamination.	Not Applicable	

**Experimental animals**	**Information included in the manuscript?**	**In which section is the information available?** (Reagents and Tools Table, Materials and Methods, Figures, Data Availability Section)
**Laboratory animals or Model organisms:** Provide species, strain, sex, age, genetic modification status. Provide accession number in repository **OR** supplier name, catalog number, clone number, **OR** RRID.	Not Applicable	
**Animal observed in or captured from the field:** Provide species, sex, and age where possible**.**	Not Applicable	
Please detail **housing and husbandry conditions**.	Not Applicable	

**Plants and microbes**	**Information included in the manuscript?**	**In which section is the information available?** (Reagents and Tools Table, Materials and Methods, Figures, Data Availability Section)
**Plants:** provide species and strain, ecotype and cultivar where relevant, unique accession number if available, and source (including location for collected wild specimens).	Not Applicable	
**Microbes:** provide species and strain, unique accession number if available, and source.	Yes	Materials and Methods; Reagent section

**Human research participants**	**Information included in the manuscript?**	**In which section is the information available?** (Reagents and Tools Table, Materials and Methods, Figures, Data Availability Section)
If collected and within the bounds of privacy constraints report on age, sex and gender or ethnicity for all study participants.	Not Applicable	

**Core facilities**	**Information included in the manuscript?**	**In which section is the information available?** (Reagents and Tools Table, Materials and Methods, Figures, Data Availability Section)
If your work benefited from core facilities, was their service mentioned in the acknowledgments section?	Not Applicable	

Design

Study protocol	Information included in the manuscript?	In which section is the information available? (Reagents and Tools Table, Materials and Methods, Figures, Data Availability Section)
If study protocol has been **pre-registered, provide DOI in the manuscript**. For clinical trials, provide the trial registration number **OR** cite DOI.	Not Applicable	
Report the **clinical trial registration number** (at ClinicalTrials.gov or equivalent), where applicable.	Not Applicable	

Laboratory protocol	Information included in the manuscript?	In which section is the information available? (Reagents and Tools Table, Materials and Methods, Figures, Data Availability Section)
Provide DOI OR other citation details if **external detailed step-by-step protocols** are available.	Yes	Materials and Methods

Experimental study design and statistics	Information included in the manuscript?	In which section is the information available? (Reagents and Tools Table, Materials and Methods, Figures, Data Availability Section)
Include a statement about **sample size** estimate even if no statistical methods were used.	Not Applicable	
Were any steps taken to minimize the effects of subjective bias when allocating animals/samples to treatment (e.g. **randomization procedure**)? If yes, have they been described?	Not Applicable	
Include a statement about **blinding** even if no blinding was done.	Not Applicable	
Describe **inclusion/exclusion criteria** if samples or animals were excluded from the analysis. Were the criteria pre-established?<br><br>If sample or data points were omitted from analysis, report if this was due to attrition or intentional exclusion and provide justification.	Not Applicable	
For every figure, are **statistical tests** justified as appropriate? Do the data meet the assumptions of the tests (e.g., normal distribution)? Describe any methods used to assess it. Is there an estimate of variation within each group of data? Is the variance similar between the groups that are being statistically compared?	Not Applicable	

Sample definition and in-laboratory replication	Information included in the manuscript?	In which section is the information available? (Reagents and Tools Table, Materials and Methods, Figures, Data Availability Section)
In the figure legends: state number of times the experiment was **replicated** in laboratory.	Yes	
In the figure legends: define whether data describe **technical or biological replicates**.	Yes	

Ethics

Ethics	Information included in the manuscript?	In which section is the information available? (Reagents and Tools Table, Materials and Methods, Figures, Data Availability Section)
Studies involving **human participants**: State details of **authority granting ethics approval** (IRB or equivalent committee(s), provide reference number for approval.	Not Applicable	
Studies involving **human participants**: Include a statement confirming that **informed consent** was obtained from all subjects and that the experiments conformed to the principles set out in the WMA Declaration of Helsinki and the Department of Health and Human Services Belmont Report.	Not Applicable	
Studies involving **human participants:** For publication of **patient photos**, include a statement confirming that consent to publish was obtained.	Not Applicable	
Studies involving experimental **animals**: State details of **authority granting ethics approva**l (IRB or equivalent committee(s), provide reference number for approval. Include a statement of compliance with ethical regulations.	Not Applicable	
Studies involving **specimen and field samples:** State if relevant **permits** obtained, provide details of authority approving study; if none were required, explain why.	Not Applicable	

Dual Use Research of Concern (DURC)	Information included in the manuscript?	In which section is the information available? (Reagents and Tools Table, Materials and Methods, Figures, Data Availability Section)
Could your study fall under dual use research restrictions? Please check biosecurity documents and list of **select agents and toxins** (CDC): https://www.selectagents.gov/sat/list.htm	Not Applicable	
If you used a select agent, is the security level of the lab appropriate and reported in the manuscript?	Not Applicable	
If a study is subject to dual use research of concern regulations, is the name of the **authority granting approval and reference number** for the regulatory approval provided in the manuscript?	Not Applicable	

Reporting

The MDAR framework recommends adoption of discipline-specific guidelines, established and endorsed through community initiatives. Journals have their own policy about requiring specific guidelines and recommendations to complement MDAR.

Adherence to community standards	Information included in the manuscript?	In which section is the information available? (Reagents and Tools Table, Materials and Methods, Figures, Data Availability Section)
State if relevant guidelines or checklists (e.g., **ICMJE, MIBBI, ARRIVE, PRISMA**) have been followed or provided.	Not Applicable	
For **tumor marker prognostic studies,** we recommend that you follow the **REMARK** reporting guidelines (see link list at top right). See author guidelines, under 'Reporting Guidelines'. Please confirm you have followed these guidelines.	Not Applicable	
For **phase II and III randomized controlled trials**, please refer to the **CONSORT** flow diagram (see link list at top right) and submit the CONSORT checklist (see link list at top right) with your submission. See author guidelines, under 'Reporting Guidelines'. Please confirm you have submitted this list.	Not Applicable	

Data Availability

Data availability	Information included in the manuscript?	In which section is the information available? (Reagents and Tools Table, Materials and Methods, Figures, Data Availability Section)
Have **primary datasets** been deposited according to the journal's guidelines (see 'Data Deposition' section) and the respective accession numbers provided in the Data Availability Section?	Not Applicable	
Were **human clinical and genomic datasets** deposited in a public access-controlled repository in accordance to ethical obligations to the patients and to the applicable consent agreement?	Not Applicable	
Are **computational models** that are central and integral to a study available without restrictions in a machine-readable form? Were the relevant accession numbers or links provided?	Yes	Supporting information
If publicly available data were reused, provide the respective **data citations in the reference list**.	Not Applicable	
